# Mogrol Attenuates Osteoclast Formation and Bone Resorption by Inhibiting the TRAF6/MAPK/NF-κB Signaling Pathway *In vitro* and Protects Against Osteoporosis in Postmenopausal Mice

**DOI:** 10.3389/fphar.2022.803880

**Published:** 2022-03-09

**Authors:** Yongjie Chen, Linlin Zhang, Zongguang Li, Zuoxing Wu, Xixi Lin, Na Li, Rong Shen, Guojun Wei, Naichun Yu, Fengqing Gong, Gang Rui, Ren Xu, Guangrong Ji

**Affiliations:** ^1^ Department of Orthopedics Surgery, Xiang’an Hospital of Xiamen University, School of Medicine, Xiamen University, Xiamen, China; ^2^ Fujian Provincial Key Laboratory of Organ and Tissue Regeneration, School of Medicine, Xiamen University, Xiamen, China; ^3^ State Key Laboratory of Cellular Stress Biology, School of Medicine, Xiamen University, Xiamen, China; ^4^ Department of Orthopedic Surgery, The First Affiliated Hospital of Xiamen University, Xiamen, China; ^5^ Guangxi Key Laboratory of Regenerative Medicine, Research Centre for Regenerative Medicine, Guangxi Medical University, Nanning, China

**Keywords:** osteoporosis, osteoclast, mogrol, TRAF6, RANKL, MAPK, NF-κB

## Abstract

Osteoporosis is a serious public health problem that results in fragility fractures, especially in postmenopausal women. Because the current therapeutic strategy for osteoporosis has various side effects, a safer and more effective treatment is worth exploring. It is important to examine natural plant extracts during new drug design due to low toxicity. Mogrol is an aglycon of mogroside, which is the active component of *Siraitia grosvenorii* (Swingle) and exhibits anti-inflammatory, anticancer and neuroprotective effects. Here, we demonstrated that mogrol dose-dependently inhibited osteoclast formation and function. To confirm the mechanism, RNA sequencing (RNA-seq), real-time PCR (RT–PCR), immunofluorescence and Western blotting were performed. The RNA-seq data revealed that mogrol had an effect on genes involved in osteoclastogenesis. Furthermore, RT–PCR indicated that mogrol suppressed osteoclastogenesis-related gene expression, including CTSK, ACP5, MMP9 and DC-STAMP, in RANKL-induced bone marrow macrophages Western blotting demonstrated that mogrol suppressed osteoclast formation by blocking TNF receptor-associated factor 6 (TRAF6)-dependent activation of the mitogen-activated protein kinase nuclear factor-B (NF-κB) signaling pathway, which decreased two vital downstream transcription factors, the nuclear factor of activated T cells calcineurin-dependent 1 (NFATc1) and c-Fos proteins expression. Furthermore, mogrol dramatically reduced bone mass loss in postmenopausal mice. In conclusion, these data showed that mogrol may be a promising procedure for osteoporosis prevention or therapy.

## Introduction

Osteoporosis is a condition that affects bone density and strength, as well as microarchitecture, resulting in fragility fractures of the hip, vertebrae, and wrist (NIH Consensus Development Panel on Osteoporosis Prevention Diagnosis and Theraphy, 2001). Approximately 25% of all women aged 65 or older are affected by osteoporosis, and rapid rates of bone loss occur postmenopausal (Ensrud and Crandall, 2017). Fractures of the hip and spine caused by osteoporosis increase the risk of fatal consequences such pneumonia and thromboembolic illness (Center et al., 1999). Therefore, osteoporosis has been one of the main health problems of postmenopausal women.

Inhibiting resorption or increasing bone formation has been selected as the main therapeutic strategy for osteoporosis. In bone-forming treatments, teriparatide and abaloparatide are the main drugs. However, nausea, headache and dizziness are common side effects of teriparatide or abaloparatide (Miller et al., 2016). Furthermore, several preclinical investigations have demonstrated that these medications enhance the incidence of osteosarcoma by concentration-dependently in rats (Vahle et al., 2008; Jolette et al., 2017). Antiresorptive medicines, such as asisphosphonates, RANKL antibodies, and selective estrogen receptor modulators (SERMs), target osteoclasts, inducing their apoptosis or inhibiting their formation or recruitment (Langdahl, 2021).

Osteoclasts are the primary agents of bone resorption. A sealing zone would be created after osteoclasts bind to the bone surface via αvβ_3_ integrin. A highly acidic microenvironment is maintained by the proton pumps and chloride channels of osteoclasts, which facilitates the catalytic activity of cathepsin K, which is a sort of lysosomal protease that breaks down collagens. Osteoclasts originate from monocytes and macrophage-derived cells that are maintained by macrophage colony-stimulating factor (M-CSF). The formation of osteoclast precursor cells to mature osteoclasts highly dependent on RANKL. Following RANKL stimulation of RANK, the key regulatory transcription factors and enzymes were activated, resulting in the promotion of osteoclast differentiation, fusion, and proliferation. TRAF6, for example, is activated when RANKL binds to RANK, which has been found to activate the protein kinase TGF-β-activated kinase (TAK1) (Xia et al., 2009). TAK1 then activated the canonical IκB kinase (IKK) complex. NF-κB, protein kinase Tp12 and mitogen-activated protein (MAP) kinase kinases (MKKs) are activated in response to IKK activation (Beinke et al., 2004; Lopez-Pelaez et al., 2014). When activated, MKKs activate c-Jun N-terminal kinases and p38 MAP kinases. MEK1 and MEK2 are activated by activated Tp12, which stimulates extracellular signal-regulated kinase 1 (ERK1) and ERK2 (Strickson et al., 2017). After activating the MAPK and NF-κB signaling pathways, NFATc1, a critical regulator of osteoclastogenesis, is eventually increased (Asagiri et al., 2005). These antiresorptive drugs treat osteoporosis by inhibiting different osteoclast physiologies. Although these drugs are effective, long-term administration is limited due to their side effects (Siris et al., 2009).

It is meaningful to find derived chemical agents or compounds that possess antiresorptive properties. Mogrol is an aglycon of mogroside, which is the active ingredient of *Siraitia grosvenorii* (Swingle). As an herbaceous native plant, *Siraitia grosvenorii* (Swingle) is a sugar substitute or traditional Chinese medicine for alleviating lung congestion, dry coughs and colds in China (Li et al., 2014). Mogrol has several pharmacological characteristics, including anti-inflammatory effects by activating AMPK signaling (Liang et al., 2021), antileukemic effects via ERK and STAT3 inhibition (Liu et al., 2015), and neuroprotective effects by inhibiting NF-κB signaling (Chen et al., 2019). Despite its great array of biological advantages, it is unknown whether mogrol affects osteoporosis and osteoclasts. This research found that mogrol suppressed the production and activity of osteoclasts derived from BMMs *in vitro*. Additionally, we also demonstrated that mogrol blocked TRAF6 recruitment, reduced MAPK and NF-B signaling activation, and ultimately restricted NFATC1 and c-FOS expression. Furthermore, we further observed that mogrol treatment reduced bone loss in mice after ovariectomy (ovaries were removed) (OVX). In summary, the outcomes of this research indicate that mogrol may promise an application for preventing or treating osteoporosis.

## Materials and Methods

### Mice and Reagents

C57BL/6J mice were acquired from Xiamen University’s Study Animal Center, and all animal investigations were authorized by Xiamen University’s Animal Care and Use Committee (XMULAC20210037). Chengdu Must Biotechnology (Chengdu, China) have supplied mogrol with a purity >98%. Dimethylsulfoxide (DMSO) was used to dissolve mogrol. Biological Industries (BI, Beit Haemek, Israel) supplied alpha-modified Eagle’s medium (α-MEM) and penicillin–streptomycin solution. Gibco (Thermo Fisher Scientific, Waltham, United States) supplemented with fetal bovine serum (FBS). Recombinant M-CSF and RANKL were acquired from R&D Systems (Minneapolis, MN, United States). Cell Signaling Technology (Danvers, United States) provided antibodies (p-ERK, ERK, p-P38, P38, p-JNK, JNK, p-P65, P65, and IκBα). Antibodies against c-FOS and TRAF6 were obtained from Abcam (Cambridge, United Kingdom). Anti-NFATc1 antibody was acquired from Santa Cruz Biotechnology (CA, United States). Anti-GAPDH antibody was purchased from Proteintech (Wuhan, China). Dojindo (Kyushu Island, Japan) provided Cell Counting Kit-8 (CCK-8). Beyotime Biotechnology, Ltd. (Shanghai, China) provided Annexin V-FITC apoptosis kits.

### Cell Culture and Differentiation Assay

BMMs were flushed from the hind leg of C57BL/6J mice aged 6 weeks and then cultivated with complete α-MEM and 25 ng/ml M-CSF. After 3 days, the media was replenished. BMMs were then digested and planted at a density of 6×10^3^ cells/well onto 96-well plates and cultivated overnight. After adherence, 30 ng/ml RANKL was used to initiate BMMs differentiation and various doses of mogrol was used to simultaneously treat these cells. Every 2 days, the cultural media were changed. Tartrate-resistant acid phosphatase (TRAP) was utilized to stain the cells fixed with 4% paraformaldehyde (PFA) on the fifth day. For the purpose of determining which stage of osteoclastogenesis was suppressed, BMMs were cultivated with mogrol and RANKL for 1, 3, or 5 days. At 6th day, the TRAP staining was conducted. Under a microscope (Olympus IX51, Japan), TRAP-positive cells with three or more nuclei were regarded as mature osteoclasts.

### Cytotoxicity Assay

CCK-8 and flow cytometry were conducted to oberve the cytotoxic impact of mogrol on BMMs. For cell viability analysis, BMMs were treated with or without varying doses of mogrol (2.5, 5, 10, 20, 40, 80, or 160 μM). After 48 or 96 h of culture, cells were treated with CCK-8 solution for 2 h before being scanned with a multimode scanner at 450 nm (Biotek, United States). Apoptotic cell death caused by mogrol was quantified utilizing an Annexin-V-FITC apoptosis detection kit. BMMs were seeded onto 10 cm disks incubated with mogrol (or 0.1% DMSO) after being allowed to adhere for 48 h. FITC-labeled Annexin V and PI were utilized to incubate the cells for 15 min after being digested, washed and resuspended, and then examined using flow cytometry (Beckman, United States).

### Immunofluorescence Staining

F-actin staining was used to explore whether mogrol affected the actin cytoskeleton. BMMs stimulated with RANKL were cultured with 20 μM mogrol on 35 mm confocal plates for 5 days, and the control group was treated with 0.1% DMSO. Next, 4% PFA was used to fix these cells, which were then permeabilized with 0.1% Triton X-100 and incubated with 3% bovine serum albumin (BSA). Following these preceding processes, rhodamine-conjugated phalloidin was tuilzed to stain the cells, and their nuclei were counterstain by 4′,6-diamidino-2-phenylindole (DAPI). BMMs were incubated overnight without or with RANKL and/or mogrol to detect the intracellular localization of P65. The cells were treated with a P65 antibody (1:200) and a secondary antibody (1:1,000) following fixation and permeabilization. DAPI was used to counterstain the nuclei. On a confocal fluorescence microscope (Leica, Germany), we analyzed the fluorescence of podosomal belts and the intracellular location of P65. Quantitative analysis of F-actin length was performed using ImageJ software.

### Bone Resorption Analysis

BMMs were planted on bovine bone slices with stimulation of 30 ng/ml RANKL for 5 days. After forming mature osteoclasts, cells were then incubated with varying doses of mogrol (0, 5, 10, 20 μM) for 3–4 days. Scanning electron microscopy was used to examine and capture resorption on bone slices. The resorption pit was calculated using ImageJ software.

### RNA-Seq and RT–PCR

Total RNA was extracted from BMMs that had been stimulated with 30 ng/ml RANKL and treated without or with 20 μM mogrol for 5 days using an RNA isolation kit (Vazyme, Nanjing, China) based on the manufacturer’s protocol. GENEWIZ, Inc. (Suzhou, Jiangsu, China) prepared and sequenced RNA libraries on an Illumina HiSeq platform. We obtained reference genome sequences indexed by Hisat2 (v2.0.1) and gene model annotation files for related species from genome websites such as UCSC, NCBI, and ENSEMBL. Hisat2 software was used to align clean data to the reference genome. Differential expression analysis was performed using DESeq2 Bioconductor software. Dispersion and logarithmic fold change estimates were computed using data-driven prior distributions, and the Padj of genes was adjusted to 0.05 to identify genes that were differentially expressed between the two groups of subjects. GOSeq (v1.34.1) was used to categorize Gene Ontology (GO) items and generate a list of enriched genes with Padj values< 0.05. Using in-house scripts, we identified significantly differentially expressed genes in KEGG pathway annotations.

RT–PCR was utilized to examine osteoclastogenesis marker gene expression. BMMs were cultured with complete α-MEM containing 25 ng/ml M-CSF, 30 ng/ml RANKL and various dose of mogrol (0, 5, 10, 20 μM) in 6-well plates. At the fifth day, total RNA was isolated utilizing an RNA isolation kit. Five hundred nanograms of RNA was utilized to reverse transcribe complementary DNA (cDNA) by Vazyme’s HiScript II Q RT SuperMix (Nanjing, China) for qPCR. The cDNA was then used as a template for qPCR, and ChamQ Universal SYBR qPCR Master Mix (Vazyme, Nanjing, China) was used to examine gene expression. The 2^-△△CT^ method was used to normalize the expression of the identified genes to that of β-actin. Table 1 contains the primer sequences.

**TABLE 1:** The primer sets used are as follows.

ACP5	Forward: 5′-TGT​GGC​CAT​CTT​TAT​GCT-3′
	Reverse: 5′-GTC​ATT​TCT​TTG​GGG​CTT-3′
DC-STAMP	Forward: 5′-CTT​GCA​ACC​TAA​GGG​CAA AG-3′
	Reverse: 5′-TCA ACAGCTCTGTCGTGA CC-3′
ATP6V0d2	Forward: 5′-GTG​AGA​CCT​TGG​AAG​ACC​TGA​A-3′
	Reverse: 5′-GAG​AAA​TGT​GCT​CAG​GGG​CT-3′
MMP-9	Forward: 5′-CGT​GTC​TGG​AGA​TTC​GAC​TTG​A-3′
	Reverse: 5′-TTG​GAA​ACT​CAC​ACG​CCA​GA-3′
CTSK	Forward: 5′-GGC​CAA​CTC​AAG​AAG​AAA​AC3′
	Reverse: 5′GTG​CTT​GCT​TCC​CTT​CTG​G-3′
β-Actin	Forward: 5′-TCT​GCT​GGA​AGG​TGG​ACA​GT-3′
	Reverse: 5′-CCT​CTA​TGC​CAA​CAC​AGT​GC-3′

Abbreviations: ACP5, acid phosphatase 5; ATP6V0d2, ATPase H+ transporting V0 subunit D2; CTSK, cathepsin K; DC-STAMP, dendritic cell-specific transmembrane protein; MMP-9, matrix metallopeptidase 9.

### Western Blotting

To observe the impact of mogrol on early RANKL-stimulated signaling events over a short time course, BMMs were fasted for 4 h, pretreated with or without 20 μM mogrol, and then activated by 50 ng/ml RANKL at 0, 5, 10, 20, 30, or 60 min in 6-well plates. To study late RANKL-stimulated signaling events, BMMs were cultured with or without 20 μM mogrol for 0, 1, 3, or 5 days with stimulation of RANKL. Total cellular proteins were extracted, separated and transferred to nitrocellulose membranes, which were then treated overnight at 4°C with moderate shaking with primary antibodies after being blocked for 1 h with 5% BSA. After rinsing the membranes, secondary antibodies conjugated to horseradish peroxidase (HRP) (CST, Danvers, MA, United States) were added to the blots. After incubation for 1 h, the membranes were submitted to chemiluminescence (Beijing Sage Creation, China).

### OVX Mouse Model

Eighteen female C5BL/6J mice aged 12 weeks were prepped to establish an OVX mouse model and then randomly split into 3 groups (*n* = 6): sham (without surgery), OVX and mogrol group (with surgery plus 10 mg/kg mogrol). After the mice were anesthetized with isoflurane, all mice underwent OVX operations or sham. A 0.5–0.8 cm incision was made in the midline of the back to remove the ovaries. One week after surgery, the mice of the sham or OVX group were administered intraperitoneally normal saline, and the mice of the mogrol group were injected 10 mg/kg mogrol intraperitoneally every second day. At 7th week, all of the mice were sacrificed. For future experiments, the femurs were collected and preserved in 70% ethyl alcohol.

### Micro-Computed Tomography (CT) Analysis

After scanning each femur using micro-CT (SkyScan, United States), the images were utilized to generate three-dimensional (3D) reconstructions of the femur. Testing parameters: 60 kV (source voltage), 160 μA (source current), rotation step of 0.4°, AI 0.25 mm filter and a pixel size of 10 μm. The NRecon program was utilized to reconstruct the images from micro-CT scans. The region of interest (ROI) of trabecular bone was set at 0.5 mm above the distal femur growth plate and 1 mm in length, while the cortical bone ROI was defined as 1 mm in height in the midpiece, and binarization was performed using a constant threshold (80–255). With the use of the cTAn program, some parameters were analyzed, including bone volume/tissue volume (BV/TV), trabecular thickness (Tb. Th), trabecular separation (Tb. Sp), trabecular number (Tb. N), bone surface/bone volume (BS/BV), and cross-sectional thickness (Cs.th).

### Histological Assessment

The femurs were then dehydrated with gradient alcohol, infiltrated with a solution containing benzoylperoside, nonylphenolpolyglykoletheracetate, and methyl methacrylate, and finally embedded in methyl methacrylate (Hahn et al., 1991). Then, the blocks were sliced into 7 μm sections for von Kossa staining or 5 μm sections for TRAP staining. Images were acquired by light microscopy (Olympus, Japan) and analyzed by Osteomeasure (OsteoMetrics, Inc., United States). In von Kossa staining, the bone parameter BV/TV was used for analysis. While The osteoclast surface/bone surface area (Oc. S/BS) and the number of osteoclasts/bone perimeter (N. Oc/B) were analyzed by TRAP staining.

### Statistical Analysis

All data in this research are shown as the mean ± standard deviation (SD). Student’s t test was used to compare two groups, and one-way analysis of variance (ANOVA) with Dunnett’s (each group compared with the control group) or Tukey’s post hoc tests (each group compared with every other group) were employed for multiple comparisons. All data were acquired from at least three independent experiments. The results with *p<0.05, **p<0.01 or ***p<0.001 were regarded as statistically significant.

## Results

### Mogrol Suppressed RANKL-Stimulated Osteoclastogenesis Without Cytotoxicity

**FIGURE 1:**
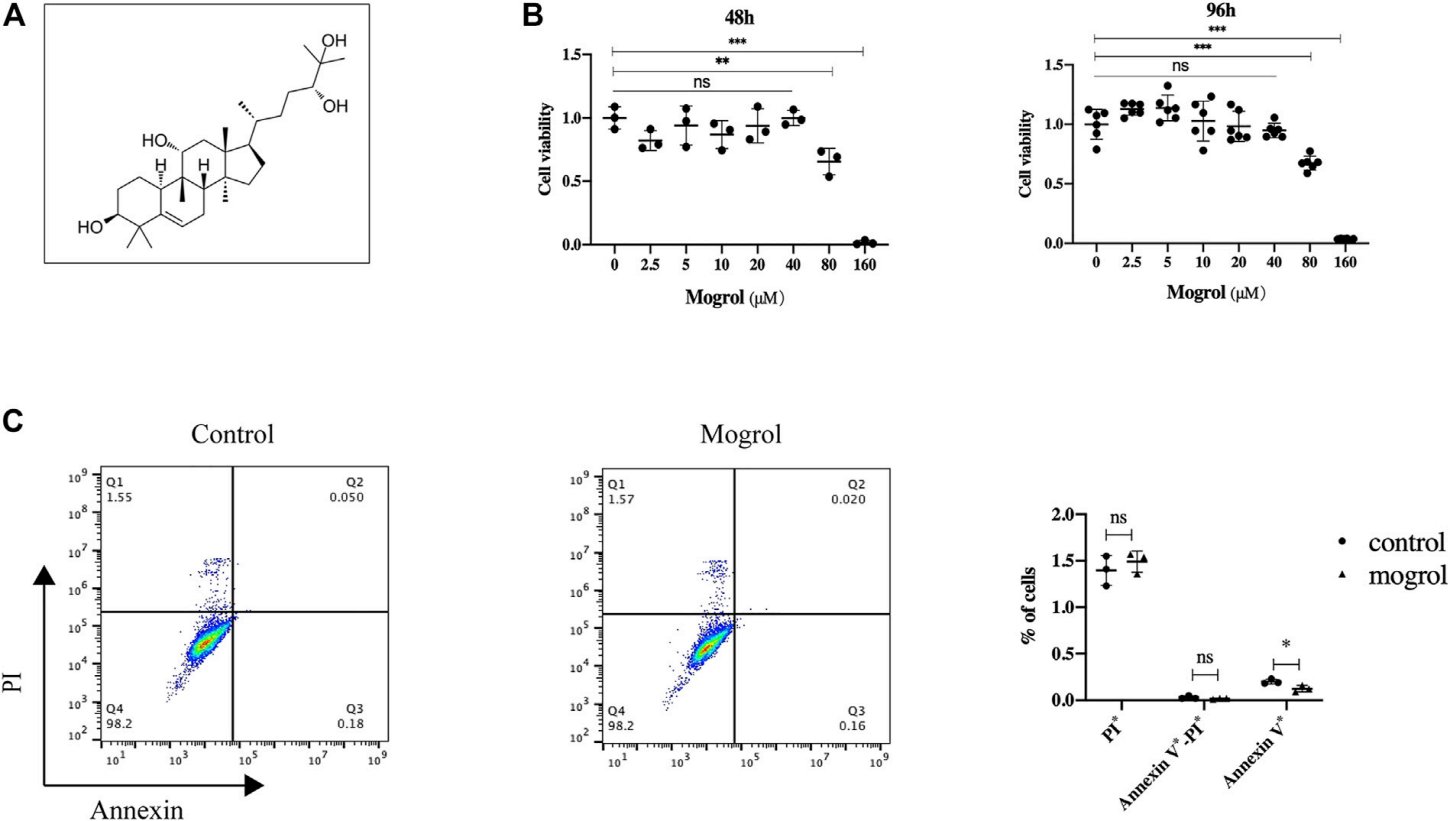
Mogrol has no toxic effect on BMMs. **(A)** Mogrol’s chemical structure. **(B)** After culturing for 48 or 96 h, a CCK-8 assay was conducted to study the impact of mogrol on BMM proliferation (*n* = 6). **(C)** Flow cytometry was utilized to analyze apoptosis of BMMs treated with 20 μM mogrol for 48 h. The histograms show the percentages of apoptotic and dead cells.

**FIGURE 2:**
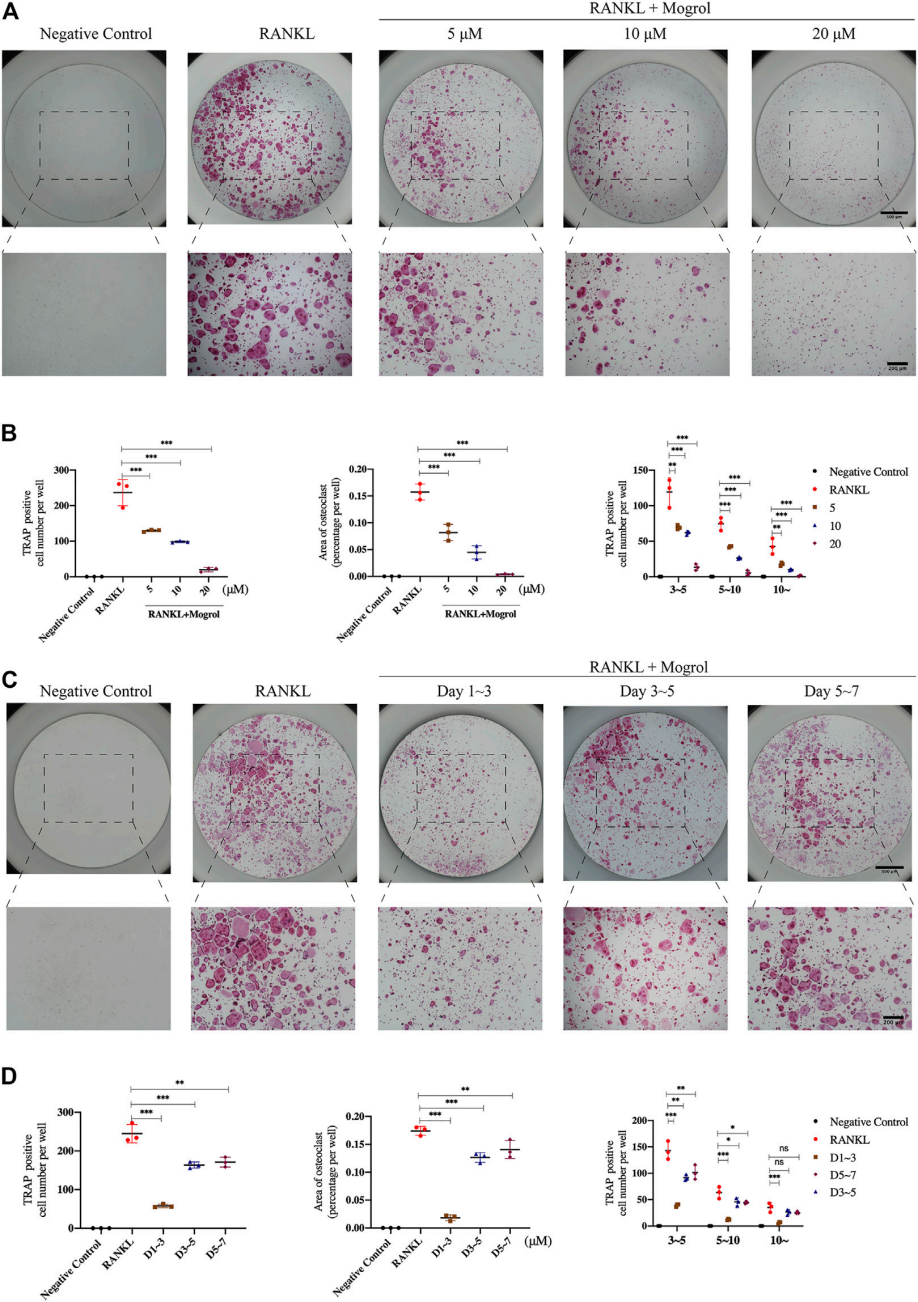
Mogrol attenuated RANKL-activated osteoclastogenesis by time- and dose-dependently. **(A)** BMMs cultured with 0, 5, 10, or 20 μM mogrol with the stimulation of 30 ng/ml RANKL (or not). After 5 days, these cells were fixed and stained with TRAP (*n* = 3). **(B)** TRAP-positive multinucleated (there or more) cells were regarded as mature osteoclasts. Quantity, area and size of mature osteoclasts were quantified and analyzed. **(C)** Images of TRAP staining of BMMs stimulated without (negative control group) or with RANKL and treated without (positive control group) or with 20 μM for 1, 3, and 5 days (*n* = 3). **(D)** Osteoclast number, osteoclast area, and osteoclast size were all quantified.

The toxicity of mogrol on BMMs was first investigated at 48 and 96 h. The CCK-8 assay revealed that mogrol (Figure 1A) had no cytotoxic effects at concentrations below 40 μM (Figure 1B). However, mogrol suppressed the proliferation of BMMs at a concentration of 80 μM and had a significant cytotoxic impact on BMMs at a concentration of 160 μM. Furthermore, there was no impact on BMM apoptosis in response to 20 μM mogrol for 48 h (Figure 1C). To demonstrate whether mogrol inhibits osteoclastogenesis, BMMs were cultivated with various dose of mogrol (0, 5, 10, 20 μM) with stimulation of RANKL. Multinucleated cells (three or more nuclei) stained for TRAP activity were regarded as mature osteoclasts. Mogrol inhibited osteoclast formation by dose-dependently. TRAP-positive multinucleated osteoclasts decreased from 236.67 ± 37.07/well in the absence of mogrol to 20.0 ± 6.08/well in the presence of 20 μM mogrol. Meanwhile, mogrol treatment significantly decreased the osteoclast area and quantity of multinucleated osteoclasts (5–10 or 10∼) by dose-dependently (Figures 2A,B). Therefore, 20 μM mogrol could inhibit osteoclast formation without cytotoxic effects. Next, BMMs activated with RANKL were cultured with or without 20 μM mogrol for 1, 3, or 5 days to demonstrate which stage of osteoclast formation was impacted. The findings indicated that mogrol’s inhibitory effect on osteoclast formation was particularly effective in the early stages (Days 1–3) (Figures 2C,D).

### Mogrol Inhibited Osteoclast Bone Resorption

**FIGURE 3:**
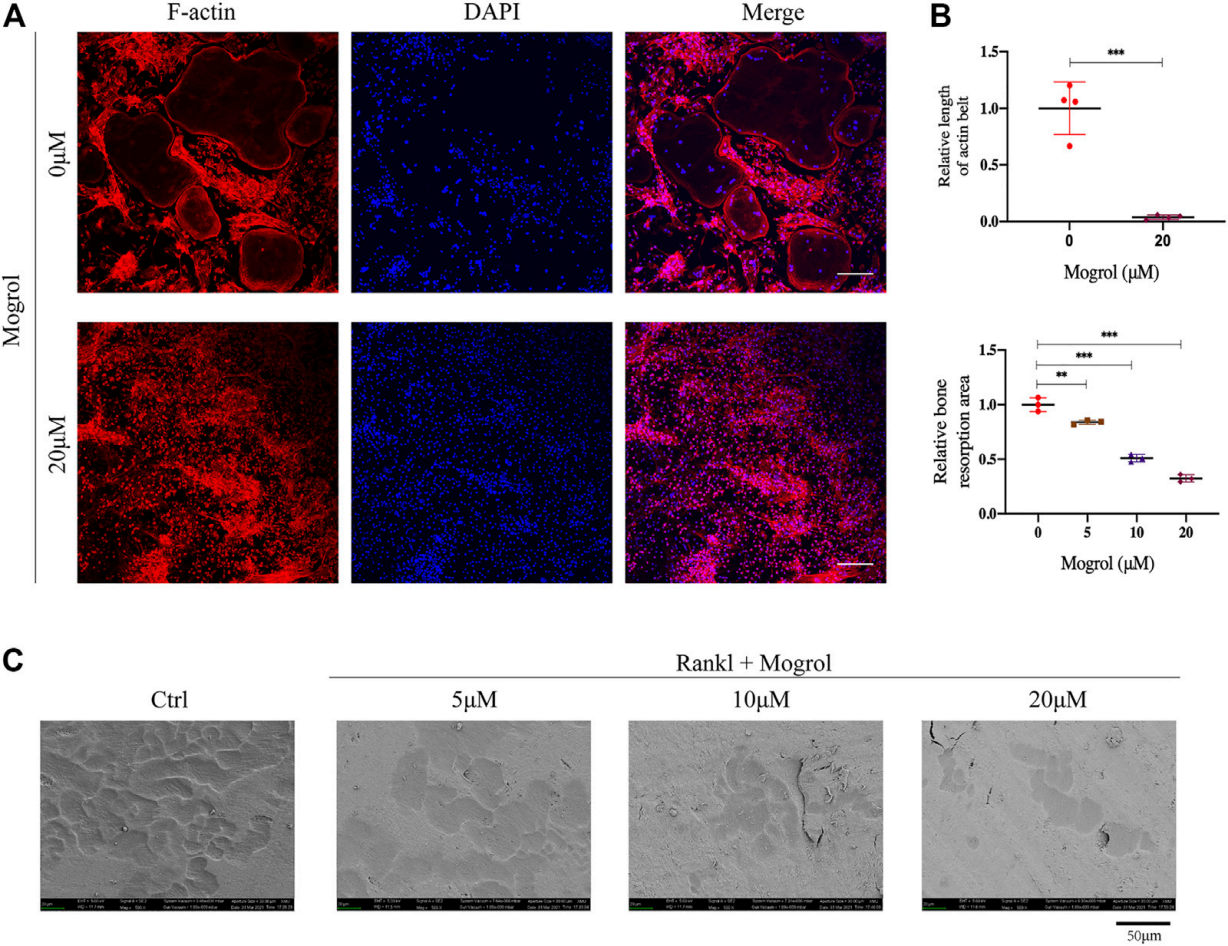
Mogrol inhibited the formation of the podosomal belt and bone resorption. **(A)** BMMs were cultivated with (or without) 20 μM mogrol with stimulation of 30 ng/ml RANKL. After 5 days, rhodamine-conjugated phalloidin was utilized to stain the podosomal belts of osteoclasts, and DAPI were used to label their nuclei (*n* = 3), scale **(B)** Images of the bone resorptive area captured by scanning electron microscopy. (*n* = 3). **(C)** ImageJ software was utilized to measure the resorption pit area and F-actin belt length. bar = 100 μm.

The impact of mogrol on the function of osteoclast was next examined by staining F-actin rings with rhodamine-phalloidin to look for morphological alterations in the osteoclast cytoskeleton linked to bone resorption. BMMs were cultivated with or without of 20 μM mogrol for 5 days with stimulation of RANKL, and immunofluorescence staining was performed. (Figure 3A). The F-actin data indicated that 20 μM mogrol considerably minified the podosomal belt, which was compatible with the retardation of osteoclast differentiation. Then, to investigate whether mogrol suppresses osteoclast bone resorption in bovine bone slices, mature osteoclasts were generated from BMMs under RANKL stimulation and cultured with 0, 5, 10, or 20 μM mogrol. Scanning electron microscopy has been utilized to observe the slices (Figure 3C), and the bone resorption areas were quantified using ImageJ software. As shown in Figure 3B, mogrol significantly decreased the resorption area by dose-dependently, indicating that mogrol inhibits osteoclast resorption function.

### RNA-Seq Results

**FIGURE 4:**
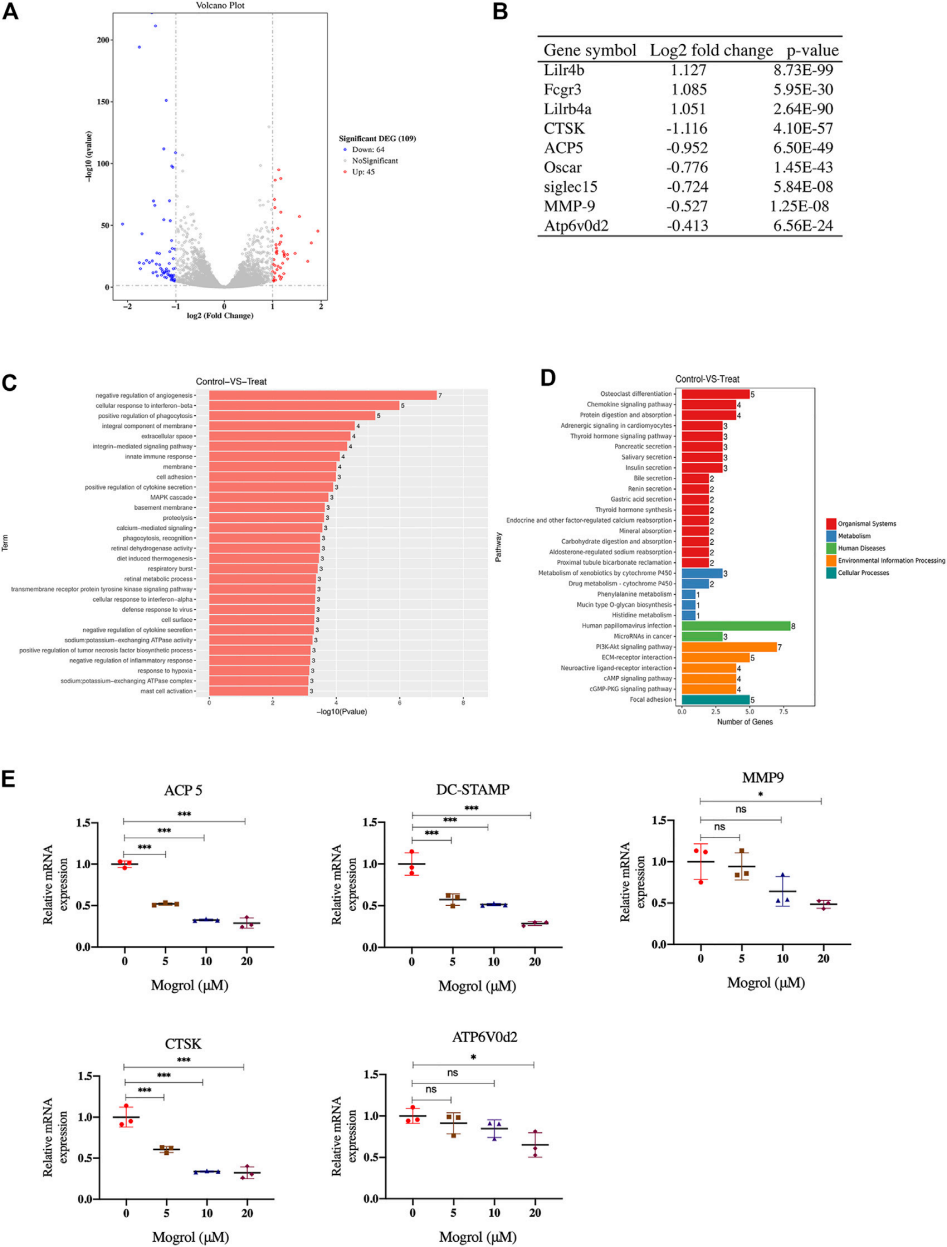
RNA-seq analysis and osteoclast marker gene expression examined by RT–PCR. Identification of genes that are regulated by mogrol. RNA was extracted from BMMs cultured with or without mogrol treatment for 5 days under RANKL stimulation. **(A)** Volcano plots of RNA-seq data in mogrol-treated BMMs compared to control BMMs. **(B)** The log2-fold change and *p* value of some osteoclastogenesis-related genes. **(C,D)** Gene Ontology. **(C)** GO terms are displayed by gene *p* values. **(D)** KEGG pathways in the differentially expressed genes are illustrated by gene numbers. **(E)** Mogrol inhibits osteoclastogenesis marker gene expression. The expression levels of *ACP5, DC-STAMP, MMP9, CTSK and ATP6V0d2* were measured using RT–PCR.

To examine how gene expression in RANKL-induced BMMs was altered by mogrol, RNA-seq was used to perform comprehensive gene expression analysis on BMMs induced by RANKL and cultured without or with mogrol for 5 days. The RNA-seq data revealed that in mogrol-treated cells, 64 genes were dramatically reduced <50%, while another 45 genes were considerably upregulated by > 2-fold compared to control cells (Figure 4A). Osteoclastogenesis suppressive genes such as lilrb4a and Fcgr3 (Onuora, 2015) were among the 45 upregulated genes, while CTSK, an osteoclast-specific gene, was among the 64 downregulated genes. Additionally, the expression of other osteoclastogenesis genes, including MMP9, OSCAR and ACP5, were lower in mogrol-treated cells. (Figure 4B). The GO analyses indicated that the biological processes innate immune response or the negative regulation of inflammation were enriched among the differentially expressed genes in mogrol-treated cells (Figure 4C). KEGG analyses also showed that the mogrol affected osteoclast differentiation (Figure 4D).

### Mogrol Inhibited Osteoclast-Specific Gene Expression

RT–PCR was utilized to confirm osteoclastogenesis marker genes expression, such as MMP9, CTSK, ATP6v0d2, ACP5, and DC-STAMP. The findings indicated that 20 μM mogrol dramatically decreased these gene expression during osteoclast development. (Figure 4E).

### Mogrol Suppressed the MAPK/NF-κB Signaling Pathway

**FIGURE 5:**
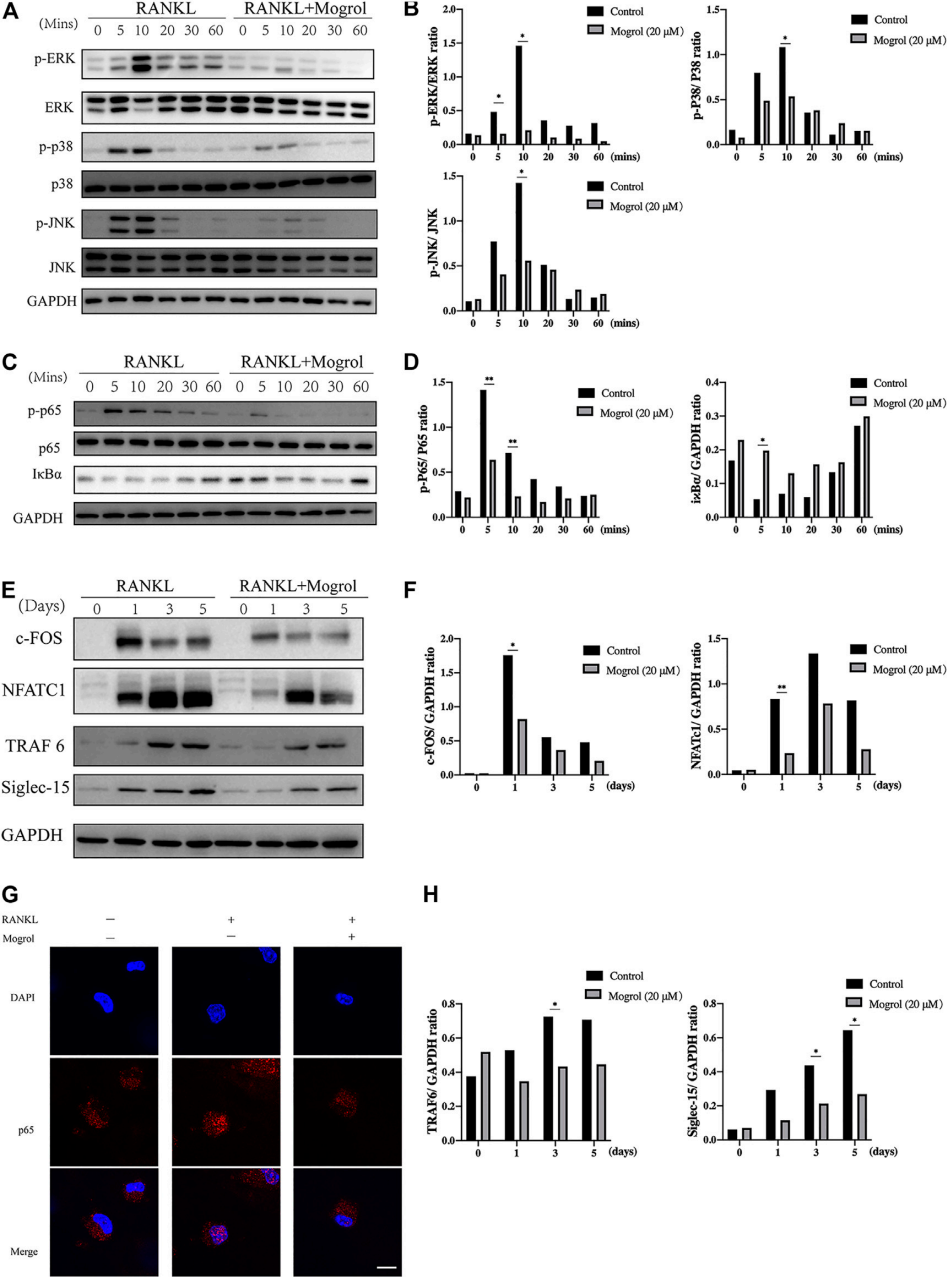
Mogrol inhibited the RANKL-dependent TRAF6/NF-κB/MAPK signaling pathways. **(A)** The impact of mogrol on RANKL-induced activation of ERK, JNK, and p38. BMMs were starved for 4 h with or without 20 μM mogrol following activation with 50 ng/ml RANKL at various time points (0, 5, 10, 20, 30, or 60 min). Specific antibodies were employed to identify the total and phosphorylated forms of ERK, JNK, and P38. **(B)** Phosphorylated ERK, JNK, and P38 Gy levels were quantified and normalized to total proteins. **(C)** RANKL-induced NF-κB p65 phosphorylation and IκBα degradation after mogrol treatment. **(D)** Using ImageJ, the gray levels of p65 or IκBα were measured and normalized to GAPDH (*n* = 3). **(E)** BMMs were cultivated with or without mogrol for 0, 1, 3, and 5 days with the stimulation of RANKL. The proteins were identified with antibodies against NFATc1, c-Fos, TRAF6, Siglec-15, or GAPDH. **(F,H)** The relative expression of NFATc1, c-Fos, TRAF6 and Siglec-15 normalized to GAPDH was quantified by the gray level using ImageJ. **(G)** Nuclear translocation of P65 was determined by immunofluorescence staining using a p65 antibody and secondary antibody (red) and observed using a confocal fluorescence microscope. Scale bar = 50 μm.

The impact of mogrol on the MAPK and NF-κB signaling pathways was investigated using western blotting to determine the mechanism by which mogrol suppresses osteoclastogenesis. Western blotting indicated that the phosphorylation of JNK and p38 were dramatically suppressed by mogrol at 10 min, while ERK phosphorylation was inhibited at 5 or 10 min (Figures 5A,B). Meanwhile, mogrol inhibited the phosphorylation of P65 at 5 or 10 min and suppressed the degradation of IκBα at 5 min (Figures 5C,D). Immunofluorescence staining confirmed this discovery, showing that mogrol efficiently blocked P65 nuclear translocation, which is required for NF-κB activation (Figure 5G).

### Mogrol Inhibited Osteoclastogenesis Proteins Expression

Subsequently, c-FOS and NFATc1 were selected to demonstrate the effect of mogrol on downstream transcription factors. BMMs activated with RANKL were cultivated with or without 20 μM mogrol for 0, 1, 3, and 5 days. The results suggested that RANKL activation increased c-FOS and NFATc1 protein expression, but mogrol substantially reduced their expression at 1 day (Figures 5E,F). In addition, mogrol suppressed TRAF6 and Siglec-15 expression at 3 or 5 days (Figures 5E–H).

### Mogrol Relieved Bone Mass Loss in OVX Mice

**FIGURE 6:**
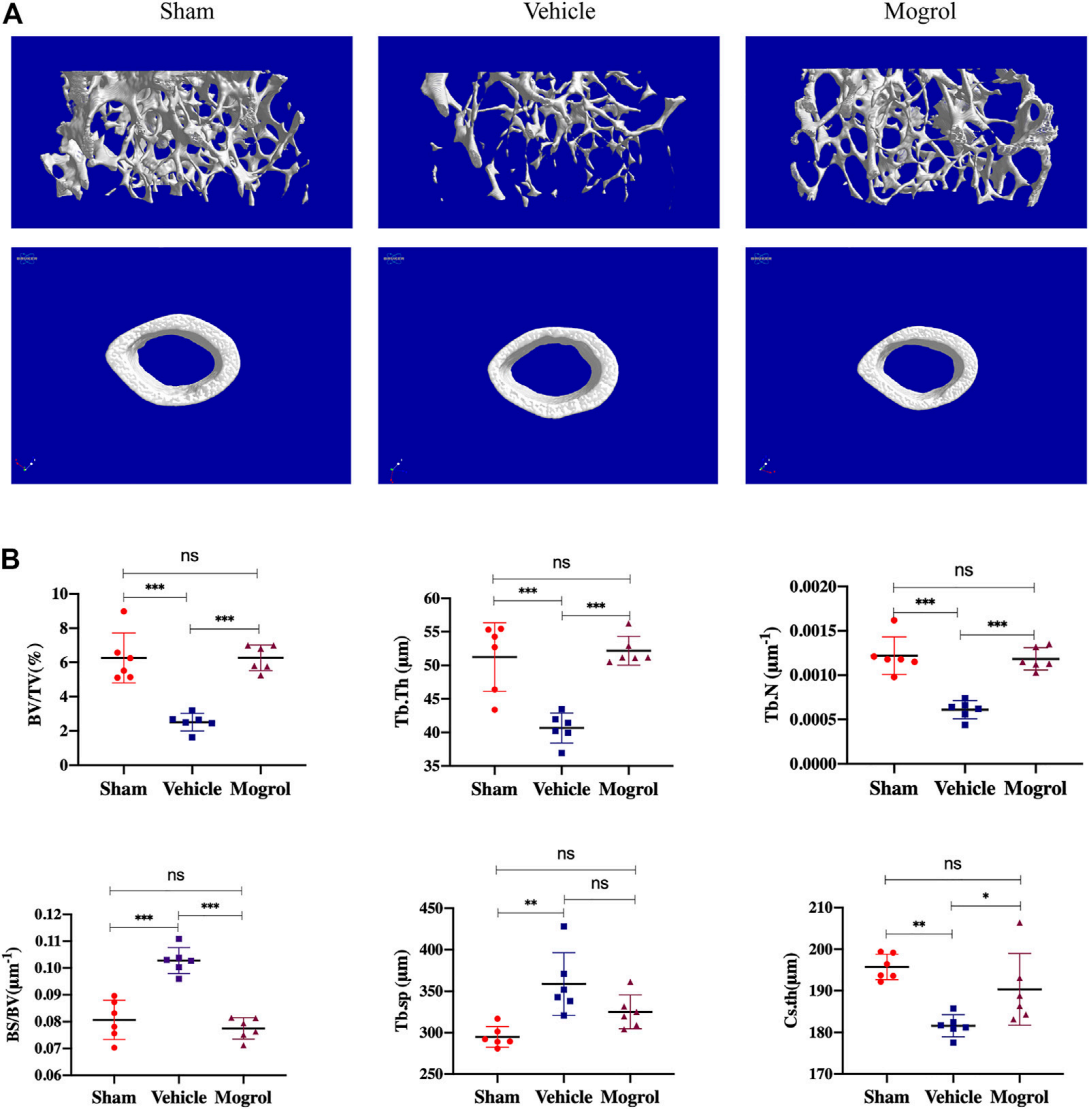
Mogrol inhibited bone mass loss in OVX mice. **(A)** 3D reconstructions of images from micro-CT scans for femurs from sham, vehicle, and mogrol groups. **(B)** Bone parameter quantitative analysis, comprising BV/TV, BS/BV, Tb. Th, Tb. N, Tb. Sp, and Cs. th. (*n* = 6).

**FIGURE 7:**
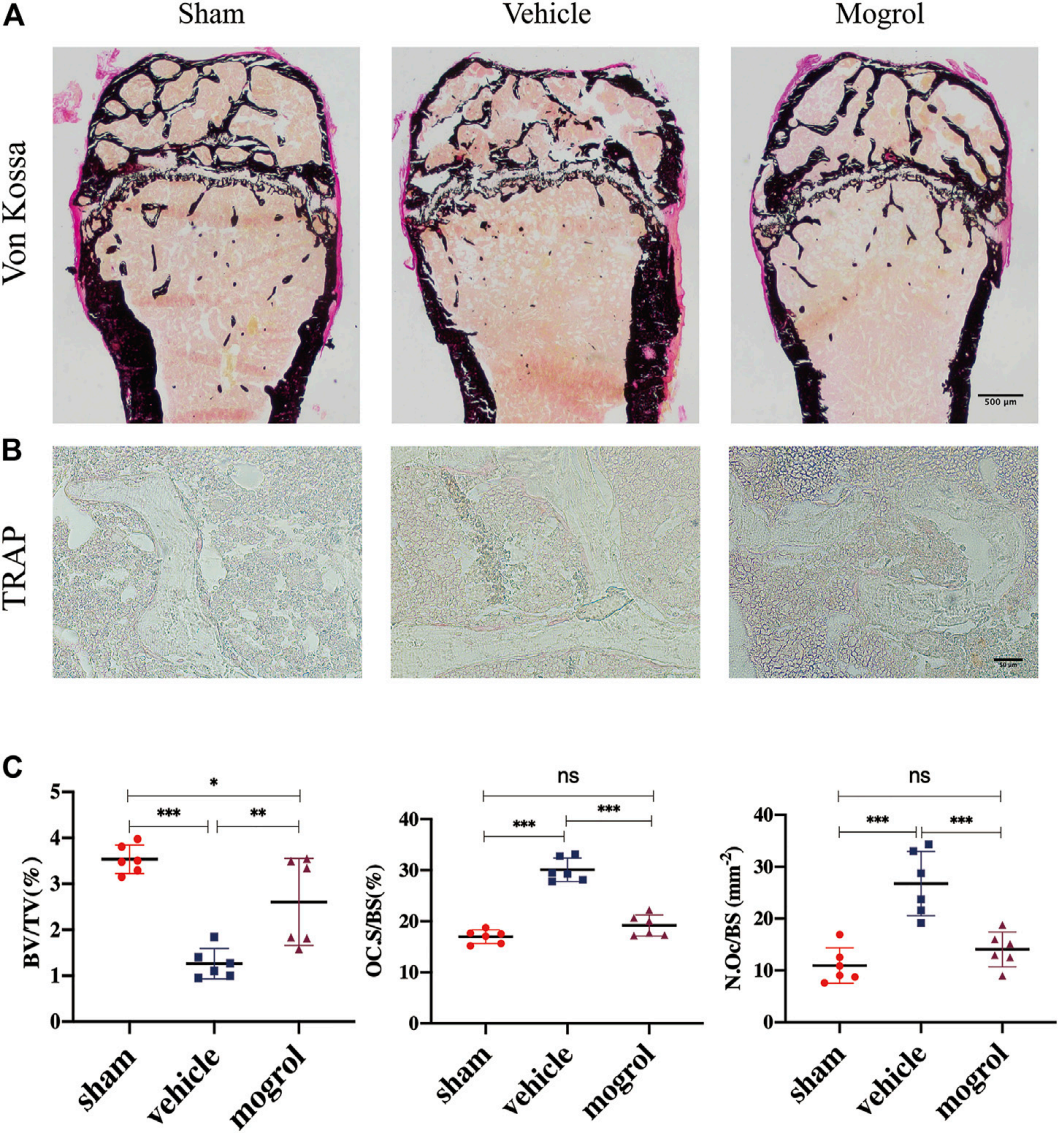
Mogrol relieved bone mass loss in OVX mice by suppressing osteoclast differentiation or activity. **(A)** Images of Von Kossa staining of the femurs. **(B)** Images of TRAP staining. **(C)** Quantitative analysis of BV/TV in Von Vossa, Oc. S/BS, and N. Oc/B in TRAP staining (*n* = 6).

Due to the encouraging findings obtained *in vitro*, the OVX mouse model was subsequently conducted to observe the inhibitory the effect of mogrol on osteoclasts *in vivo*. Mice were administered mogrol or normal saline every second day for 42 days. Micro-CT 3D reconstructions indicated that mogrol decreased bone loss of femurs in OVX mice. The quantitative analysis of micro-CT showed that the parameters BV/TV, Tb. Th, Tb. N and Cs. th of vehicle group were lower than sham group, while Tb. Sp and BS/BV were higher. However, BV/TV, Tb. Th, Tb. The N and Cs. th of the mogrol group were considerably higher than those of the vehicle group, while the BS/BV was inversely lower (Figures 6A,B). Histological assessment was conducted to further demonstrate the protective impact of Mogrol against bone loss after OVX. The Von Kossa results demonstrated that the BV/TV of the vehicle group was much lower than that of the sham group, while the BV/TV of the mogrol group was dramatically higher than that of the vehicle group (Figures 7A,C). Additionally, immunohistochemistry TRAP staining of femurs was used to assess the mogrol’s impact on osteoclasts in OVX mice. As shown in Figures 7B,C, Oc. The S/BS and N. Oc/B of the mogrol group were substantially lower than those of the vehicle group. The results of animal study suggested that mogrol might dramatically attenuate bone mass loss in OVX mice.

## Discussion

Osteoporosis is the most common chronic bone metabolic condition and affects at least 200 million individuals globally over the age of 60 (Lane, 2006). As a result, there are more than 9 million fractures annually worldwide, with approximately one fracture every 3 s, due to osteoporosis. In particular, up to 40% of postmenopausal women suffer from osteoporosis (De Martinis et al., 2021). Moreover, 61% of osteoporotic fractures, including 70% of hip fractures, occur in women (Johnell and Kanis, 2006). Bone mineral density (BMD) is utilized in the clinic to identify and assess osteoporosis, which has a heritability of 0.6–0.8. Numerous osteoporosis susceptibility loci, including ESR1, LRP4, DAAM2, WNT 16, and SOX 6, have been found and investigated using multiomics techniques based on systems genetics and genomics. Of note, drugs targeting susceptibility loci are more effective in clinical trials than those without genetic support (Yang et al., 2020). In addition to genomics studies, single-cell RNA sequencing (scRNA-Seq) has been used in osteoporosis research. For example, multipotent human skeletal stem cells are capable of forming bone and cartilage by scRNA-Seq. Due to its superiority, scRNA-Seq supports the viability of exploring the underlying cellular mechanisms of osteoporosis (Chan et al., 2018). Additionally, the gut microbiota which affects bone metabolism is receiving increasing attention in osteoporosis research (Chen et al., 2017).

For osteoporosis treatment, different drugs have been exploited. These drugs include bisphosphonates, RANKL antibodies, SERMs, and bone-anabolic agents. However, side effects such as esophageal irritation, thromboembolic disease, hypercalcemia, and renal toxicity limit the use of these drugs (Curry et al., 2018; Langdahl, 2021). These side effects prevent osteoporosis patients from taking these anti-osteoporosis drugs, particularly bisphosphonates (Khosla and Hofbauer, 2017). As a result, there is still a need to discover new medications that are free of these adverse effects and could be applied long-term. Recently, natural plant extracts have been an attractive alternative drug for osteoporosis due to their low toxicity (Zhan et al., 2019; Lin et al., 2020; Wang et al., 2021). Our study showed that mogrol, an aglycon of mogroside extracted from *Siraitia grosvenorii* (Swingle), has an inhibitory impact on osteoclast differentiation and function *in vitro* and protects against bone mass loss in postmenopausal mice.

RANKL, a member of the tumor necrosis factor superfamily, is produced by osteoblasts and osteocytes in the skeletal system (Suda et al., 1999; Nakashima et al., 2011). When RANKL binds to RANK, which is abundantly expressed in preosteoclasts, it results in an increase in TRAF6, which further stimulates downstream signaling pathways during osteoclastogenesis (Kobayashi et al., 2001). For example, in the NF-κB signaling pathways, the recruitment of TRAF6 induces the phosphorylation of IκBα, leading to its subsequent degradation. Following IκBα breakdown, P65 is phosphorylated and transported from the cytoplasm to the nucleus, activating downstream transcription factors during osteoclastogenesis. (Jimi et al., 2019). It has been demonstrated that mogrol exerted neuroprotection against memory impairment and neuroinflammation by inhibiting the NF-κB signaling pathway (Chen et al., 2019). As shown by western blotting, mogrol inhibited IκBα protein degradation and p65 phosphorylation. Furthermore, the immunofluorescence staining results demonstrated that mogrol blocked RANKL-activated p65 nuclear translocation. These results demonstrated that mogrol inhibited the NF-κB signaling pathway to suppress osteoclast formation.

RANKL also activates the MAPK signaling pathway, which is comprised of the proteins JNK, P38, and ERK during osteoclastogenesis. Concurrent with NF-κB signaling pathway activation, TRAF6 recruitment induces the formation of a complex including TAK-1 and TAB. TAK1 stimulates IKKs and MKKs, resulting in the phosphorylation of JNK1, JNK2, and p38 MAP kinases. IKKβ activates protein kinase Tpl2 (MAP3K8) as a component of the IKK complex, and subsequently MEK1 and MEK2 are activated (MAP or ERK) (Strickson et al., 2017). Previously published research established that mogrol inhibited leukemia cell proliferation by suppressing ERK1/2 phosphorylation (Liu et al., 2015). Our Western blotting analysis indicated that mogrol suppressed the phosphorylation of ERK, P38 or JNK in the MAPK signaling pathway. Therefore, the NF-κB and MAPK signaling pathways were blocked by mogrol. We next demonstrated that mogrol reduced the levels of TRAF6 in BMMs using western blotting. Mogrol, on the other hand, has a suppressive impact on TRAF6. TRAF6-related downstream signaling pathways, including MAPK, NF-κB, and ROS, are regulated by K63- or K48-linked ubiquitination. K63-linked ubiquitination of TRAF6 activates TAK-1, which is upstream of MAPKs and the NF-κB pathway, as described above. Act1 was identified to mediate K63-linked ubiquitination of TRAF6 (Wu et al., 2015). In contrast, TRAF6 degradation is promoted by K48-linked ubiquitination, which suppresses RANKL-induced osteoclastogenesis. Different molecules, such as IPMK, RNF19a and TRIM38, were identified as regulators of the K48-linked ubiquitination of TRAF6 (Kim et al., 2017; Wu et al., 2017). Recent research has discovered that some natural extracts promote TRAF6 degradation by enhancing K48-linked ubiquitination, such as icartin (Tan et al., 2017) and curcumenol, which suppress IPMK (Wang et al., 2021). There is a possibility that mogrol inhibits osteoclastogenesis by affecting the regulator of K63- or K48-linked ubiquitination. Nevertheless, further research on the underlying mechanism is required.

The c-Fos and Jun proteins, downstream of the NF-κB and MAPK signaling pathways, were dimerized to form AP-1, which was recruited to the promoter region of the NFATc1 gene to induce its expression, which subsequently promoted a variety of osteoclastogenic genes expression, such as Dcstamp, Atp6v0d2, ACP5, MMP9 and Ctsk. To determine how expression was altered by mogrol in RANKL-induced BMMs, RNA-seq was conducted. The RNA-seq results demonstrated that mogrol may be related to osteoclast differentiation, such as by upregulating the osteoclast inhibitory factors lilirb4a and fcgr3 or downregulating osteoclastogenesis-related genes such as ACP5, Dcstamp, MMP9 and Ctsk. Then, using real-time PCR, we demonstrated that mogrol reduced osteoclastogenic gene expression in BMMs. Consistent with the osteoclastogenesis results, mogrol decreased NFATc1 and c-Fos expression. Thus, we hypothesized that mogrol blocks osteoclast formation by inhibiting the NF-κB and MAPK signaling pathways by reducing TRAF6. Interestingly, we found that mogrol also suppressed Siglec-15 expression. Siglec-15 appears to promote the activation of ERK, AKT, or PI3K to modulate osteoclast differentiation as a member of the Siglec family of glycan-recognition proteins. Murine BMM and human osteoclast precursor differentiation into osteoclasts was suppressed by utilizing anti-Siglec-15 antibodies. There are still undisclosed signaling pathways regulated by Siglec-15 in osteoclast differentiation. Mogrol inhibited osteoclast fusion, as shown by TRAP staining, which was consistent with the western blot findings showing Siglec-15 suppression. However, it is unclear whether the inhibitory effect of mogrol on Siglec-15 is due to direct influences or reductions in the number of mature osteoclasts. It is worthwhile to further explore.

Finally, we also showed that intraperitoneal treatment with mogrol relieved bone mass loss in OVX mice *via* suppressing osteoclast activity, according to micro-CT and histological assessment. Interestingly, mogrol also rescued bone cortex thinning induced by OVX. Estrogen is essential for bone development and remodeling. Menopause or surgical ovariectomy results in trabecular and cortical bone loss by increasing osteoclast production and function. Endocortical resorption was increased after estrogen withdrawal in cortical bone (Väänänen and Härkönen, 1996). In addition, the production of inflammatory cytokines and RANKL promote cortical bone erosion by stimulating osteoclast differentiation and resorptive activity in bone disorders (Schett and Gravallese, 2012). These results indicated that osteoclasts are an important factor in bone metabolism. As shown in our results, in OVX mice, mogrol reduced cortical and trabecular bone loss, likely by inhibiting osteoclast development and activity *in vivo*. Notably, though a single dose treatment for OVX-mediated bone loss is limited, this is a first study to assess the effect of mogrol in pathological animal model of postmenopausal osteoporosis in detail. Additionally, due to the pharmacodynamics and pharmacokinetics of mogrol are largely unclear, further studies focusing on the magnitude of mogrol need to address the optimization of dosing and delivery strategies for ameliorating bone loss.

In conclusion, our study indicated that mogrol suppresses osteoclastogenesis and resorption by inhibiting the RANKL-dependent TRAF6/NF-κB/MAPK signaling pathway and relieved bone mass loss in postmenopausal mice. These findings demonstrate that mogrol could be an attractive natural extract to be used as a nontoxic drug for treating osteoclast-mediated osteolytic disorders such as osteoporosis.

## Data Availability

The data presented in the study are deposited in the GEO repository, accession number GSE196742.
