# Supplementary material for: Mogrol Attenuates Osteoclast Formation and Bone Resorption by Inhibiting the TRAF6/MAPK/NF-κB Signaling Pathway In vitro and Protects Against Osteoporosis in Postmenopausal Mice
Source: Front Pharmacol. 2022 Mar 9;13:803880. doi: 10.3389/fphar.2022.803880 (PMC9038946; doi:10.3389/fphar.2022.803880)
Supplement: Supplementary file 1 [file DataSheet3.ZIP › Data-figure4/RNA-Seq/Table 2.docx]

Table 2. Osteoclastogensis related genes

| Gene symbol | Log2 fold change | p-value |
| --- | --- | --- |
| Lilr4b | 1.127 | 8.73E-99 |
| Fcgr3 | 1.085 | 5.95E-30 |
| Lilrb4a | 1.051 | 2.64E-90 |
| CTSK | -1.116 | 4.10E-57 |
| ACP5 | -0.952 | 6.50E-49 |
| Oscar | -0.776 | 1.45E-43 |
| siglec15 | -0.724 | 5.84E-08 |
| MMP-9 | -0.527 | 1.25E-08 |
| Atp6v0d2 | -0.413 | 6.56E-24 |
