# Supplementary figures and images for: Mogrol Attenuates Osteoclast Formation and Bone Resorption by Inhibiting the TRAF6/MAPK/NF-κB Signaling Pathway In vitro and Protects Against Osteoporosis in Postmenopausal Mice
Source: Front Pharmacol. 2022 Mar 9;13:803880. doi: 10.3389/fphar.2022.803880 (PMC9038946; doi:10.3389/fphar.2022.803880)

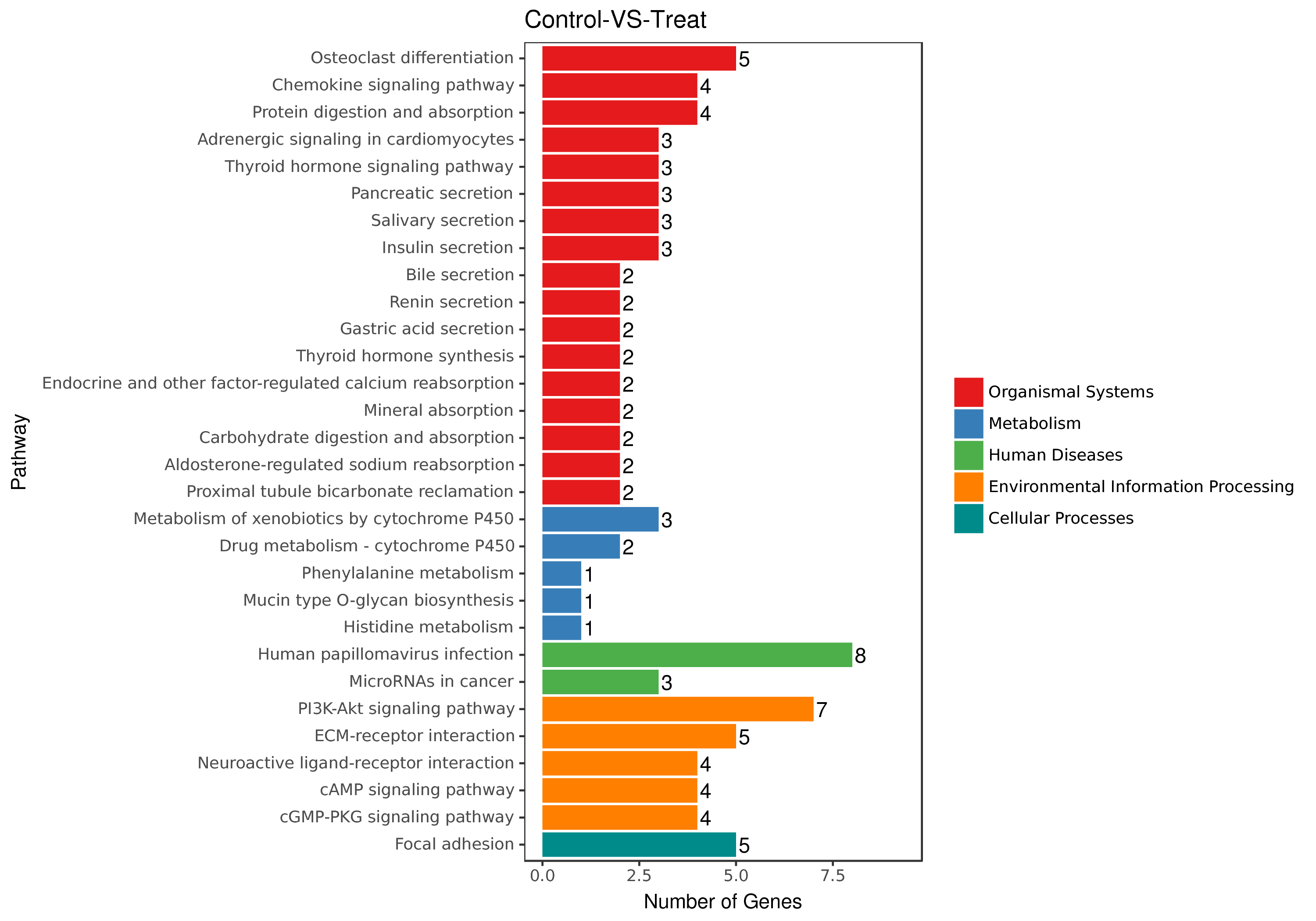

Supplement: Supplementary file 1 [file DataSheet3.ZIP › Data-figure4/RNA-Seq/Control-VS-Treat_PathwayBar.png]

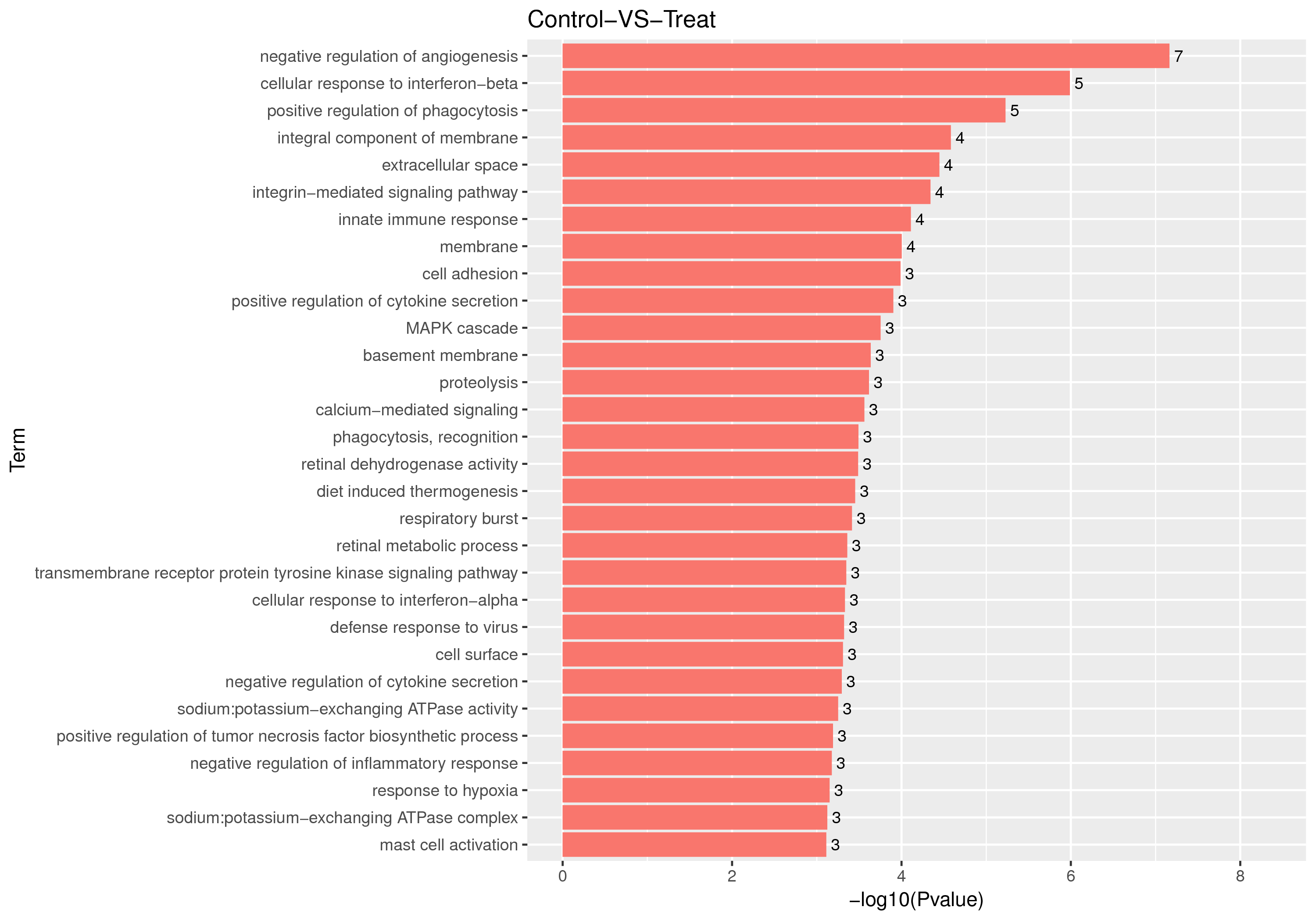

Supplement: Supplementary file 1 [file DataSheet3.ZIP › Data-figure4/RNA-Seq/Control-VS-Treat_GO_pvalue.png]

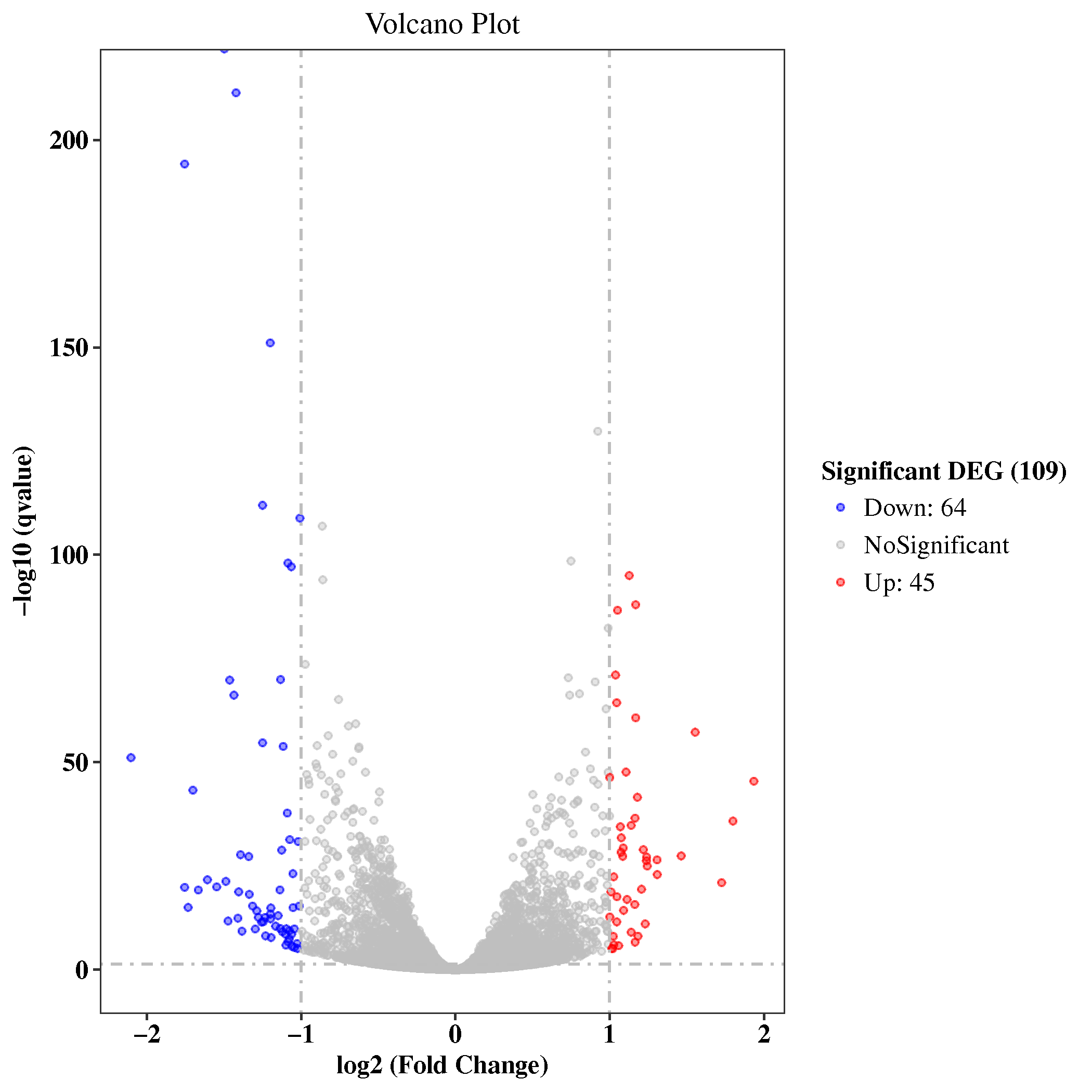

Supplement: Supplementary file 1 [file DataSheet3.ZIP › Data-figure4/RNA-Seq/Control-VS-Treat_results.Volcano.png]

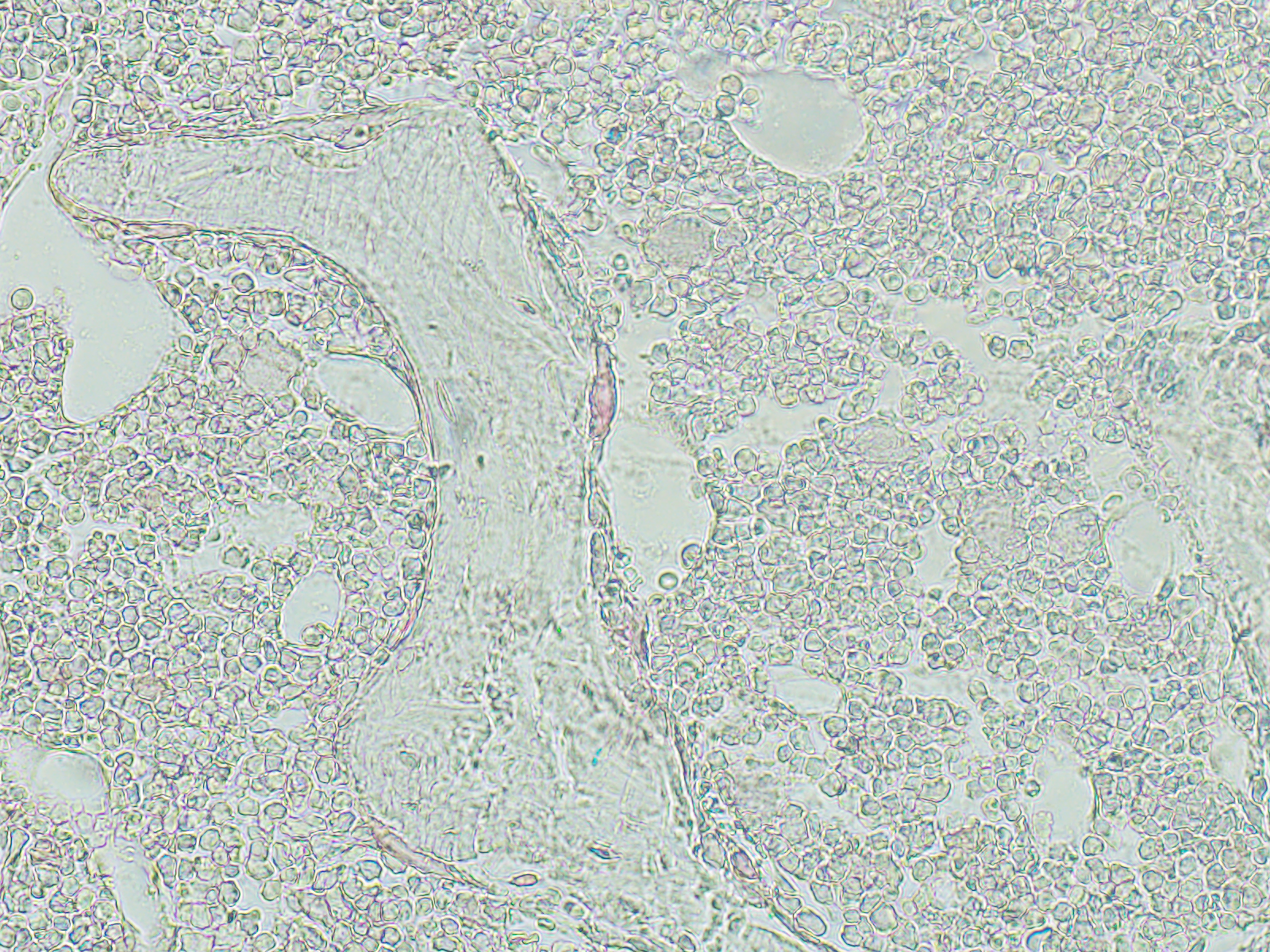

Supplement: Supplementary file 2 [file DataSheet8.ZIP › figure 7 data/TRAP/sham.tif]

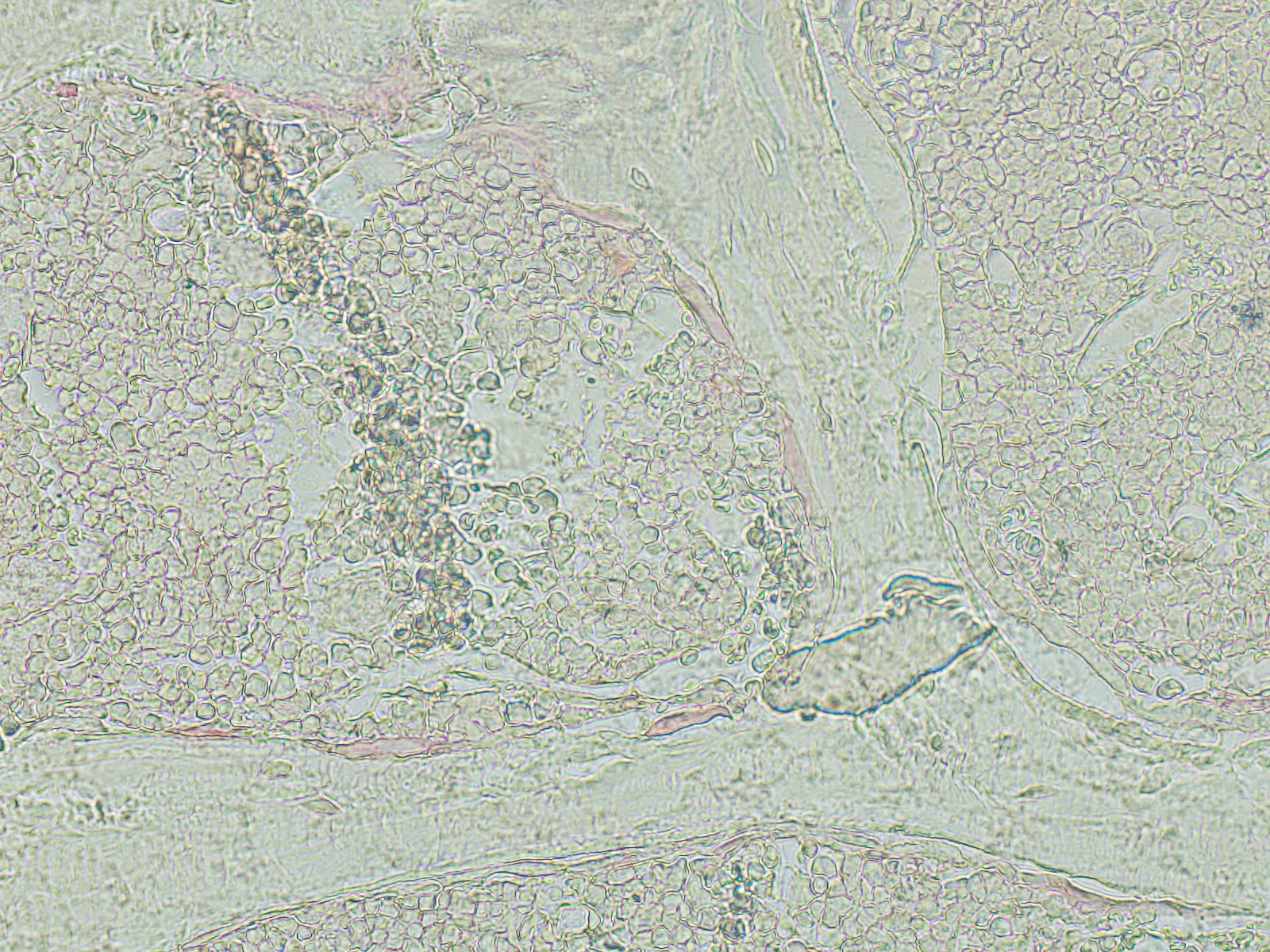

Supplement: Supplementary file 2 [file DataSheet8.ZIP › figure 7 data/TRAP/vehicle.tif]

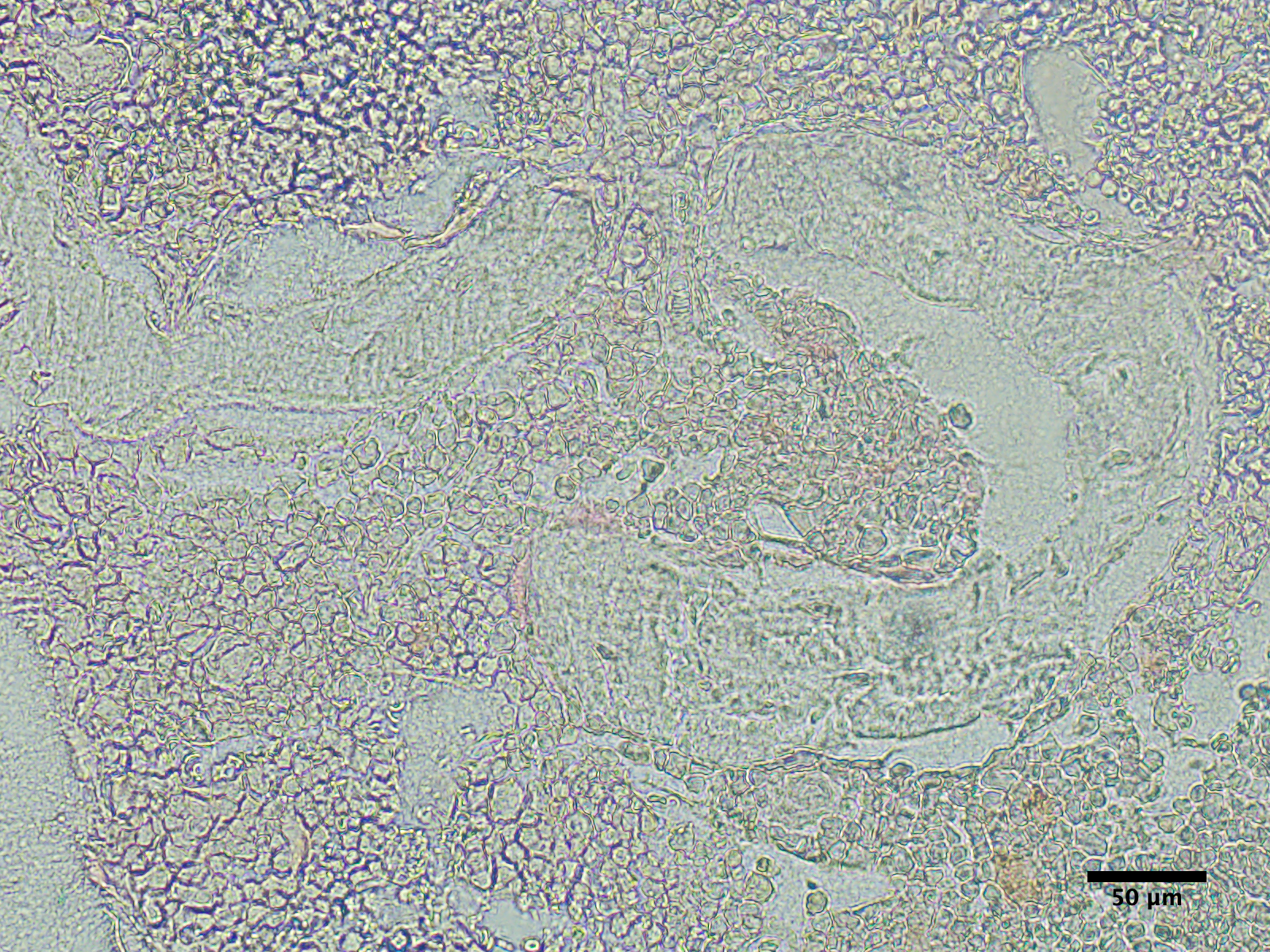

Supplement: Supplementary file 2 [file DataSheet8.ZIP › figure 7 data/TRAP/mogrol2.tif]

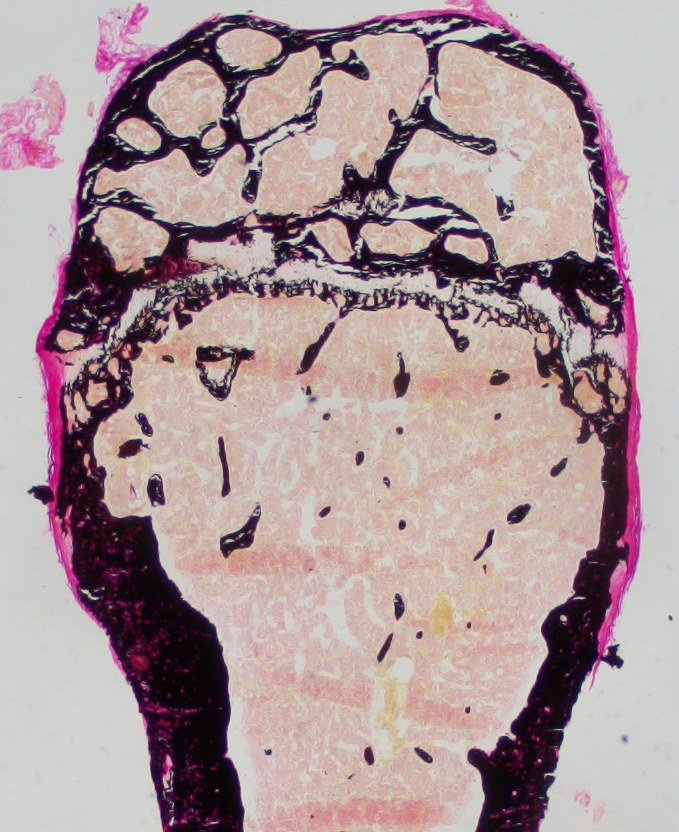

Supplement: Supplementary file 2 [file DataSheet8.ZIP › figure 7 data/von kossa/Sham.tif]

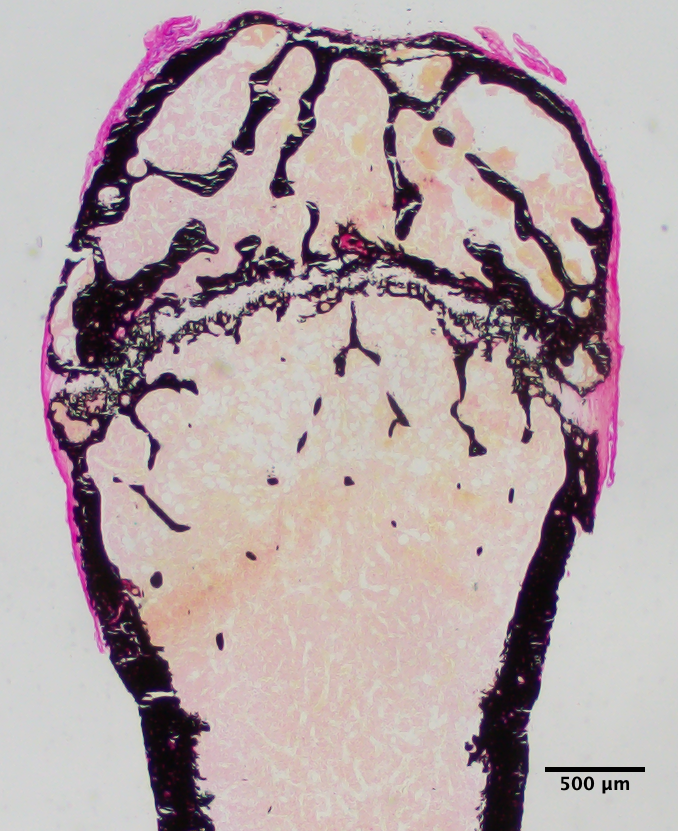

Supplement: Supplementary file 2 [file DataSheet8.ZIP › figure 7 data/von kossa/Mogrol.tif]

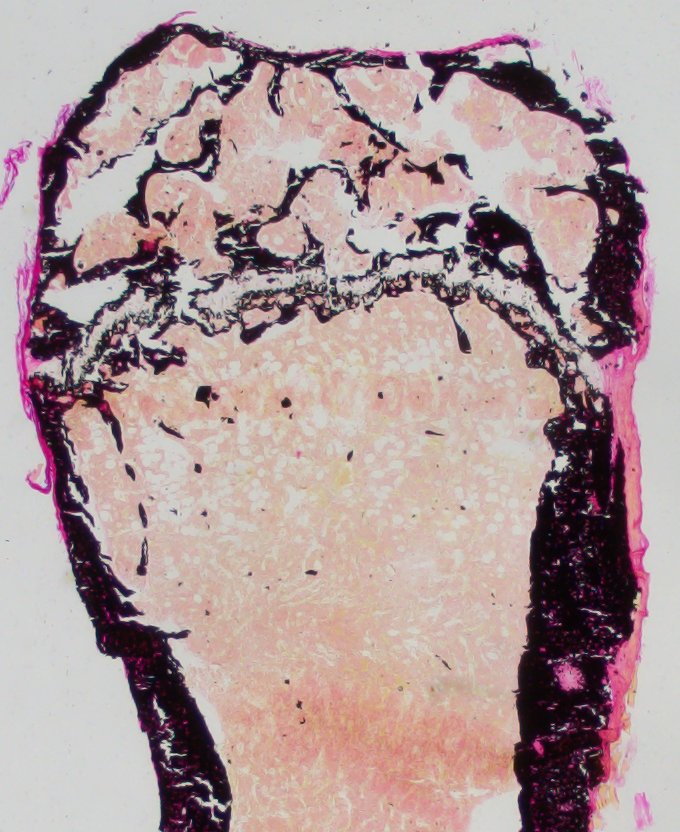

Supplement: Supplementary file 2 [file DataSheet8.ZIP › figure 7 data/von kossa/Vehicle.tif]

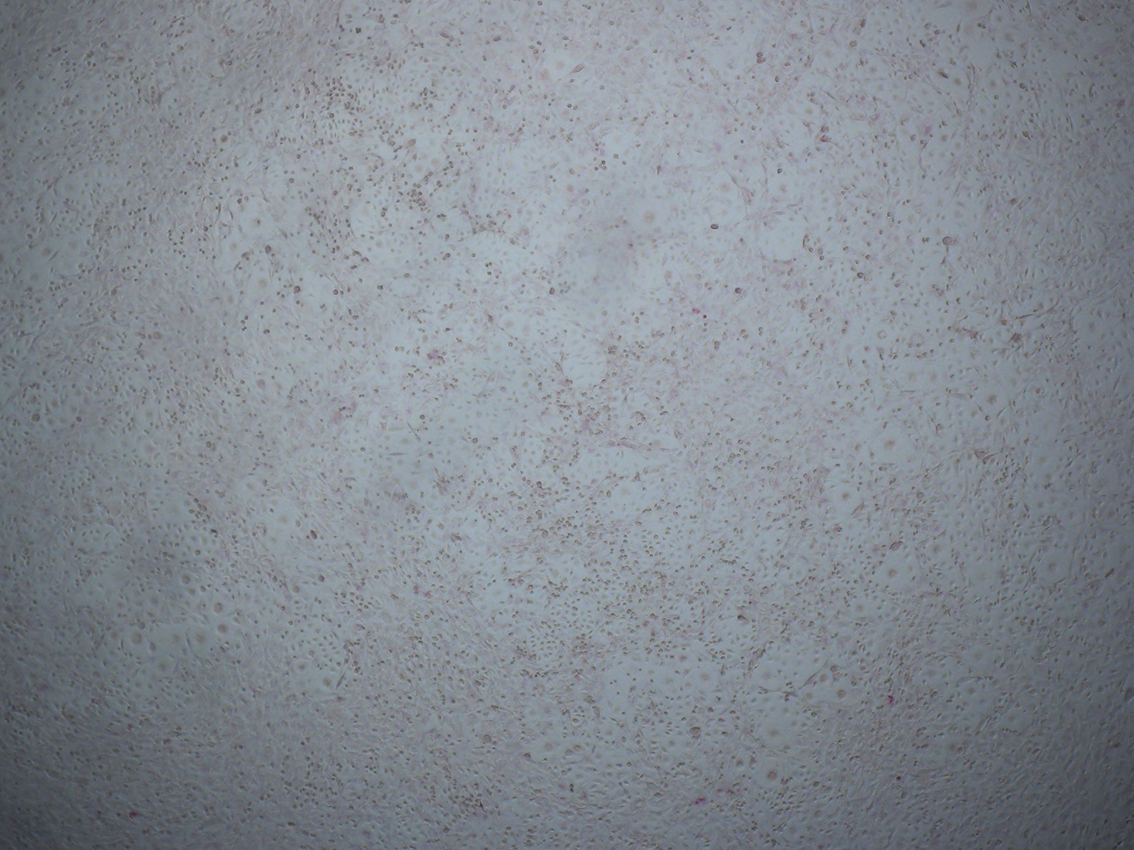

Supplement: Supplementary file 3 [file DataSheet9.ZIP › supplementary image data(1)/A/10uM.tif]

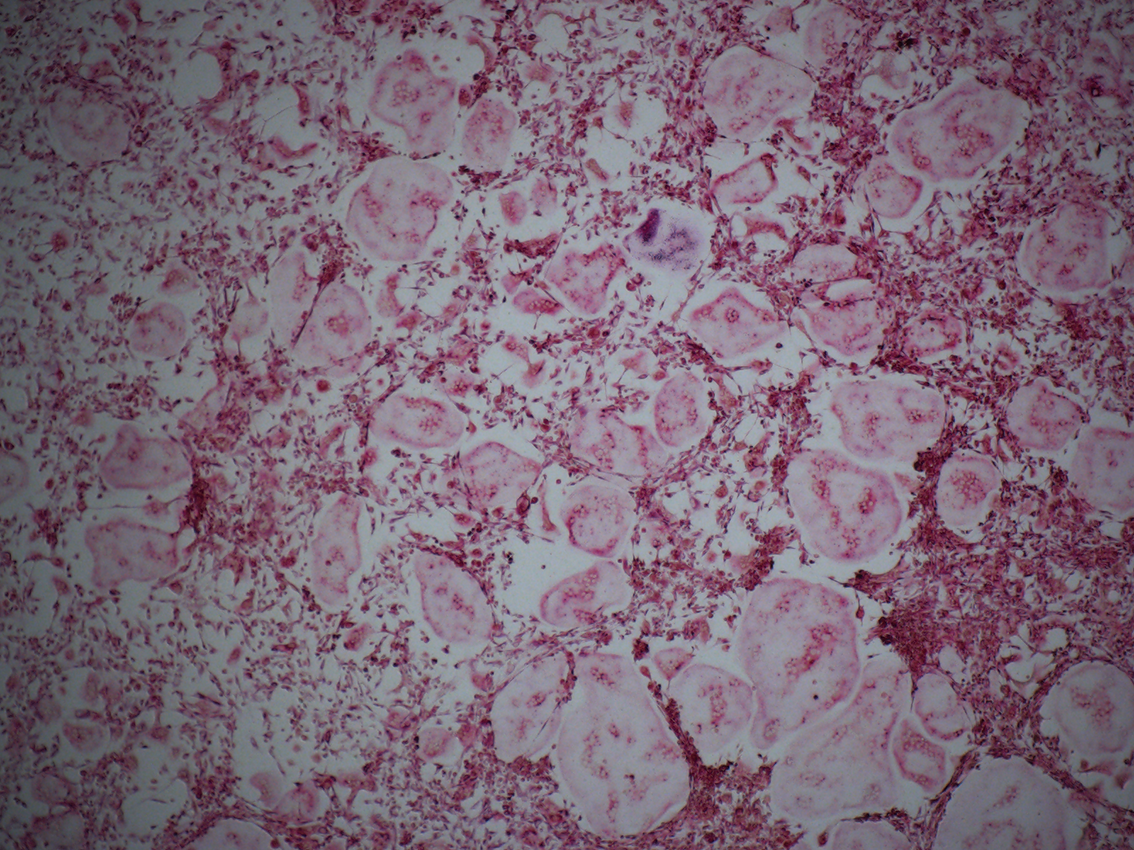

Supplement: Supplementary file 3 [file DataSheet9.ZIP › supplementary image data(1)/A/rankl.tif]

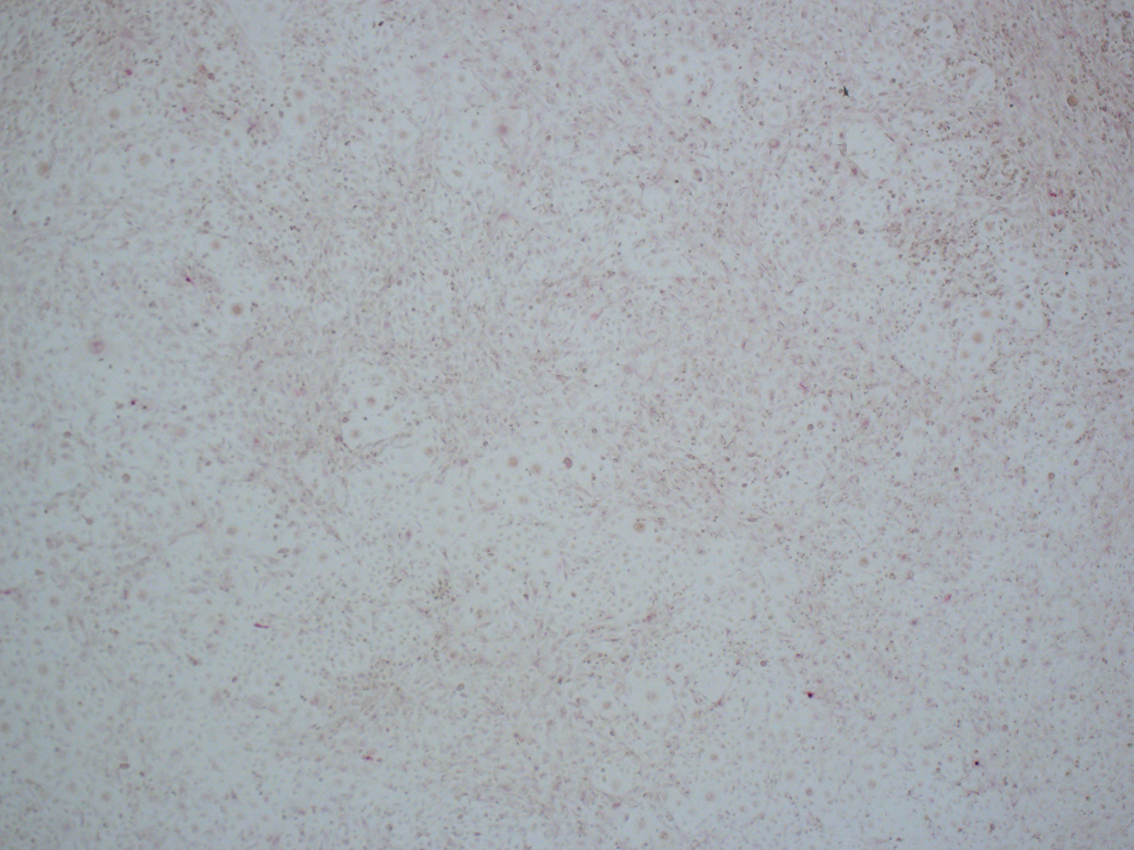

Supplement: Supplementary file 3 [file DataSheet9.ZIP › supplementary image data(1)/A/0.tif]

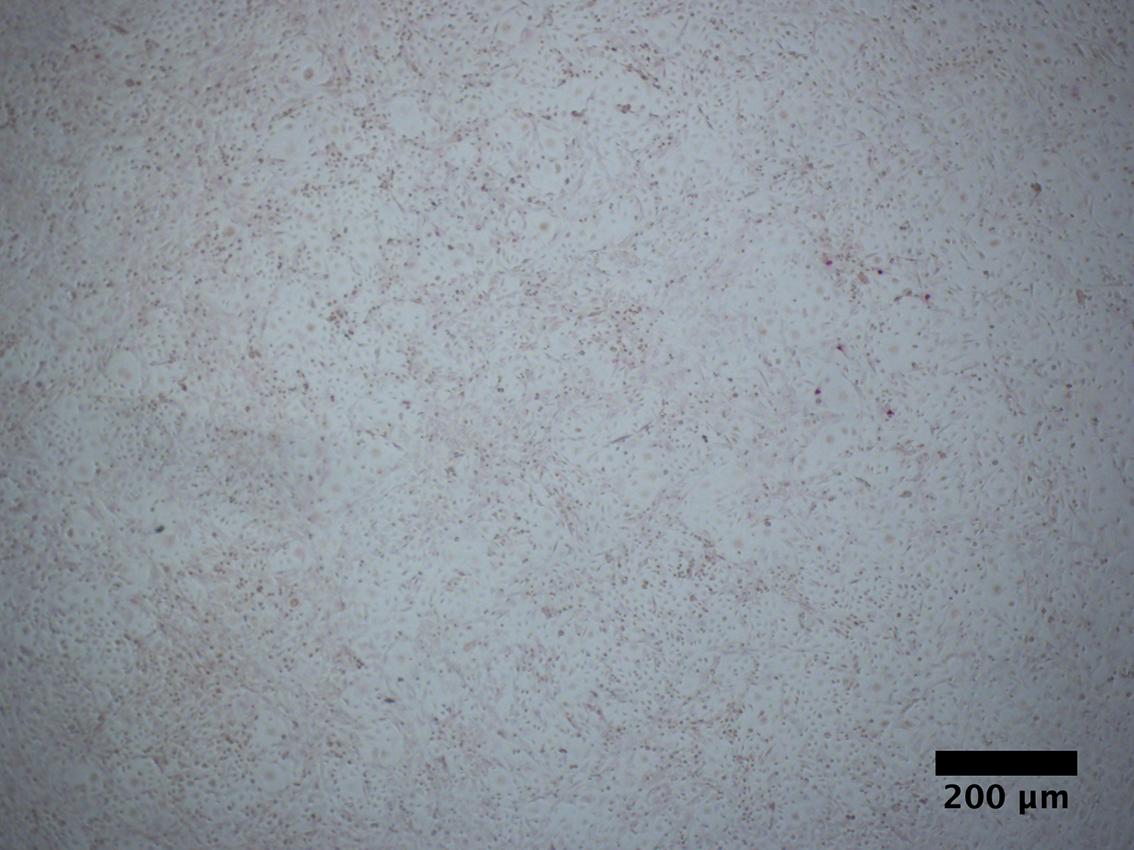

Supplement: Supplementary file 3 [file DataSheet9.ZIP › supplementary image data(1)/A/20uM.tif]

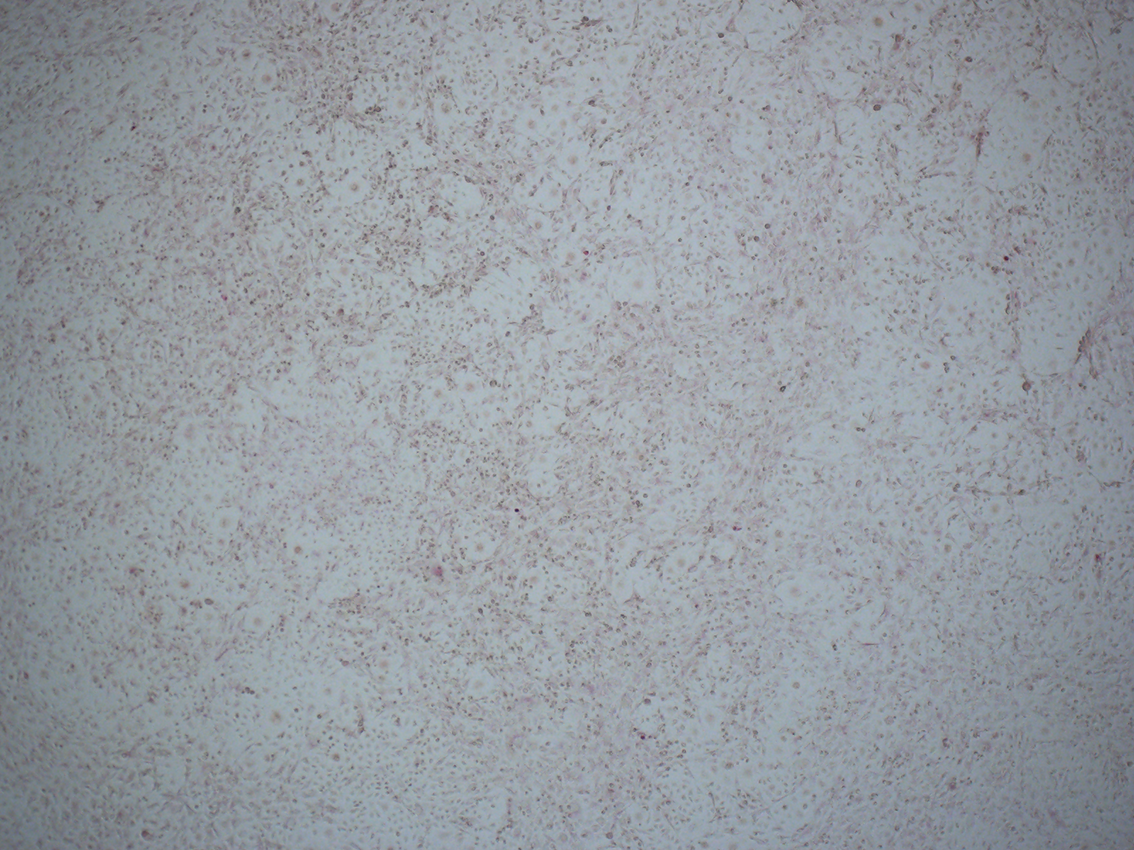

Supplement: Supplementary file 3 [file DataSheet9.ZIP › supplementary image data(1)/A/5uM.tif]

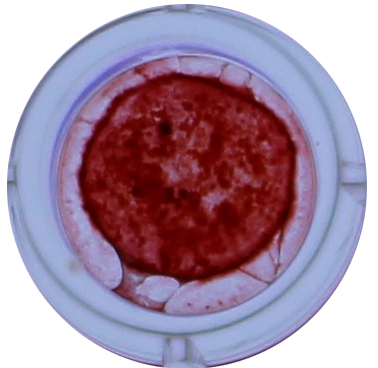

Supplement: Supplementary file 3 [file DataSheet9.ZIP › supplementary image data(1)/C/10uM1.jpg]

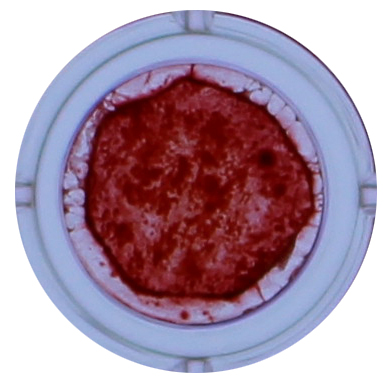

Supplement: Supplementary file 3 [file DataSheet9.ZIP › supplementary image data(1)/C/5uM1.jpg]

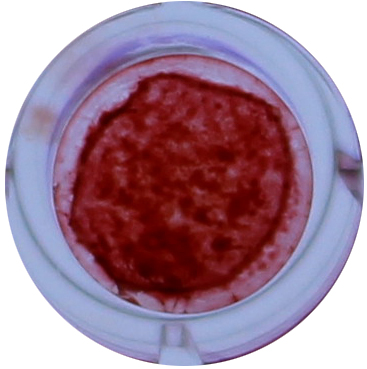

Supplement: Supplementary file 3 [file DataSheet9.ZIP › supplementary image data(1)/C/20uM1.jpg]

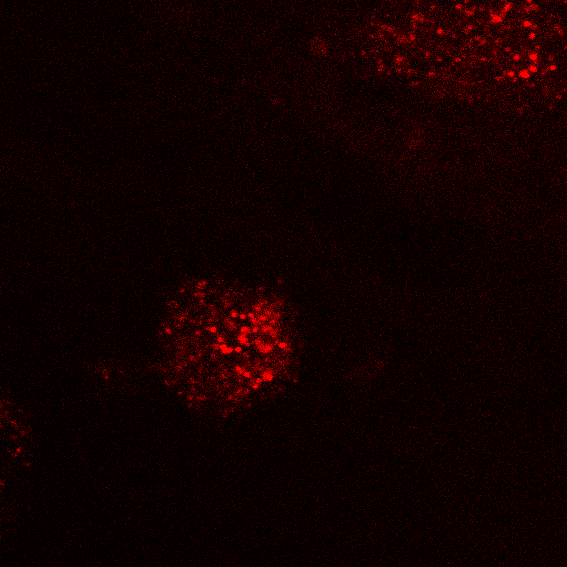

Supplement: Supplementary file 4 [file DataSheet4.ZIP › Data-figure5/p65/RANKL1.tif]

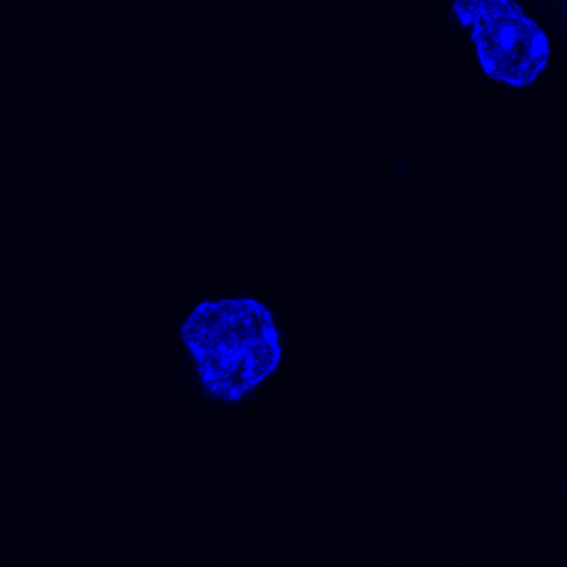

Supplement: Supplementary file 4 [file DataSheet4.ZIP › Data-figure5/p65/RANKL3.tif]

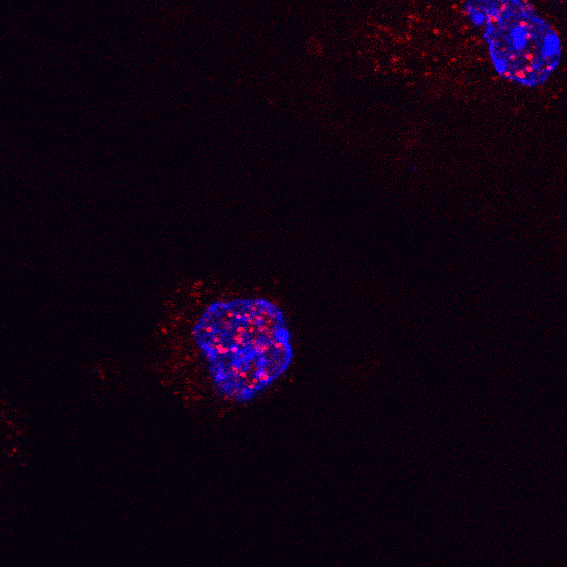

Supplement: Supplementary file 4 [file DataSheet4.ZIP › Data-figure5/p65/RANKL2.tif]

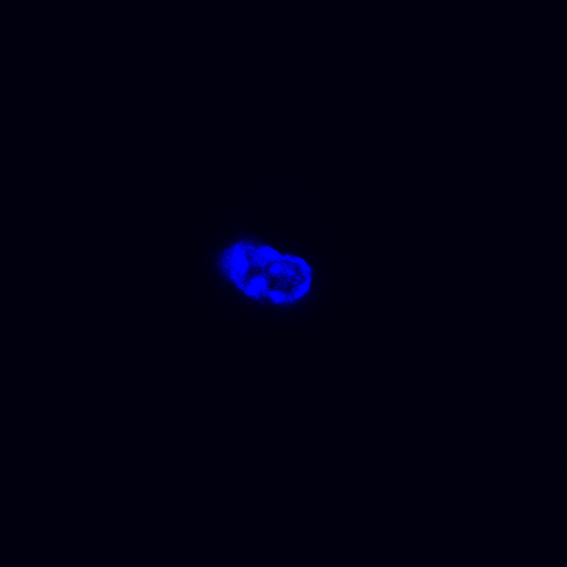

Supplement: Supplementary file 4 [file DataSheet4.ZIP › Data-figure5/p65/mogrol3.tif]

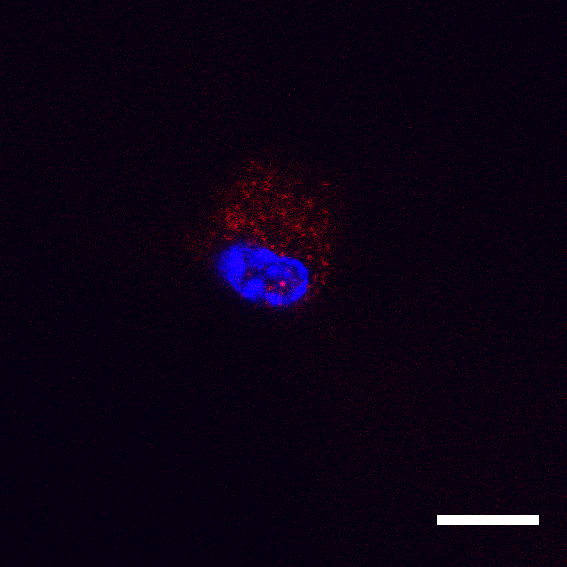

Supplement: Supplementary file 4 [file DataSheet4.ZIP › Data-figure5/p65/mogrol2.tif]

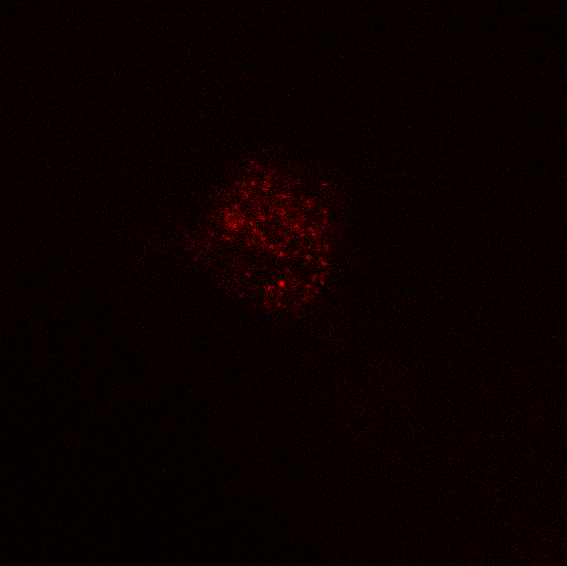

Supplement: Supplementary file 4 [file DataSheet4.ZIP › Data-figure5/p65/mogrol1.tif]

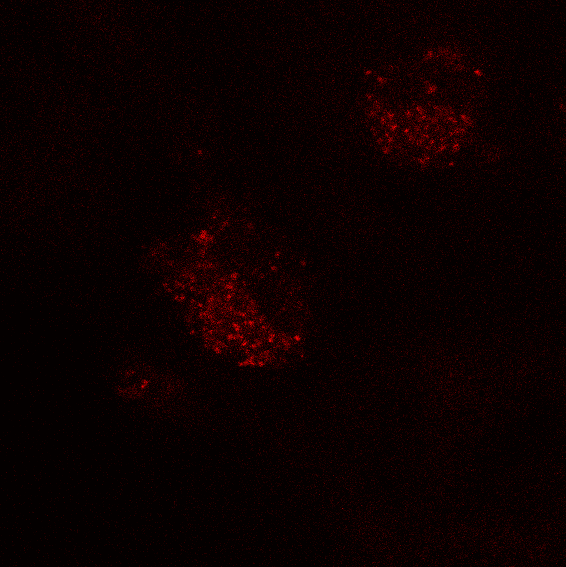

Supplement: Supplementary file 4 [file DataSheet4.ZIP › Data-figure5/p65/ctrl1.tif]

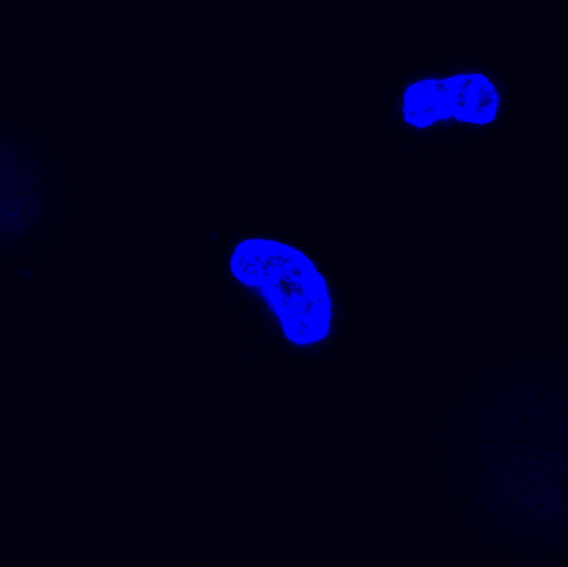

Supplement: Supplementary file 4 [file DataSheet4.ZIP › Data-figure5/p65/ctrl3.tif]

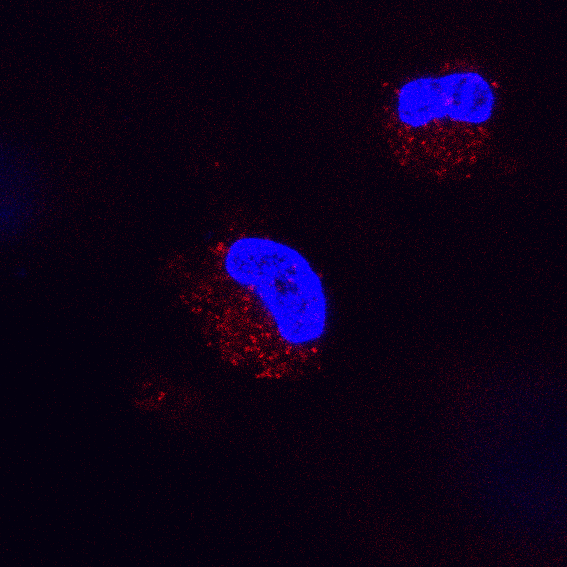

Supplement: Supplementary file 4 [file DataSheet4.ZIP › Data-figure5/p65/ctrl2.tif]

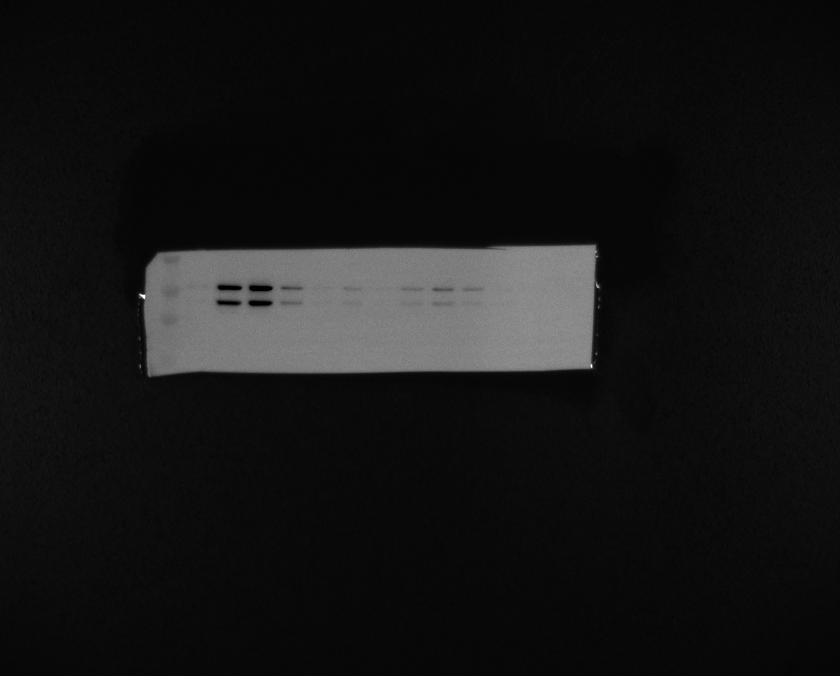

Supplement: Supplementary file 4 [file DataSheet4.ZIP › Data-figure5/western blot/WB image/pJNK.tiff]

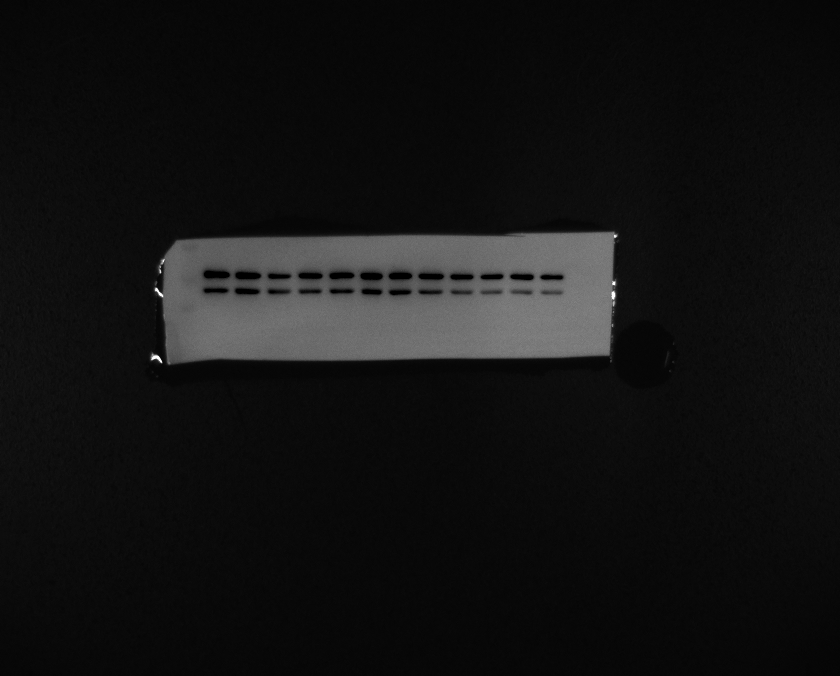

Supplement: Supplementary file 4 [file DataSheet4.ZIP › Data-figure5/western blot/WB image/JNK.tiff]

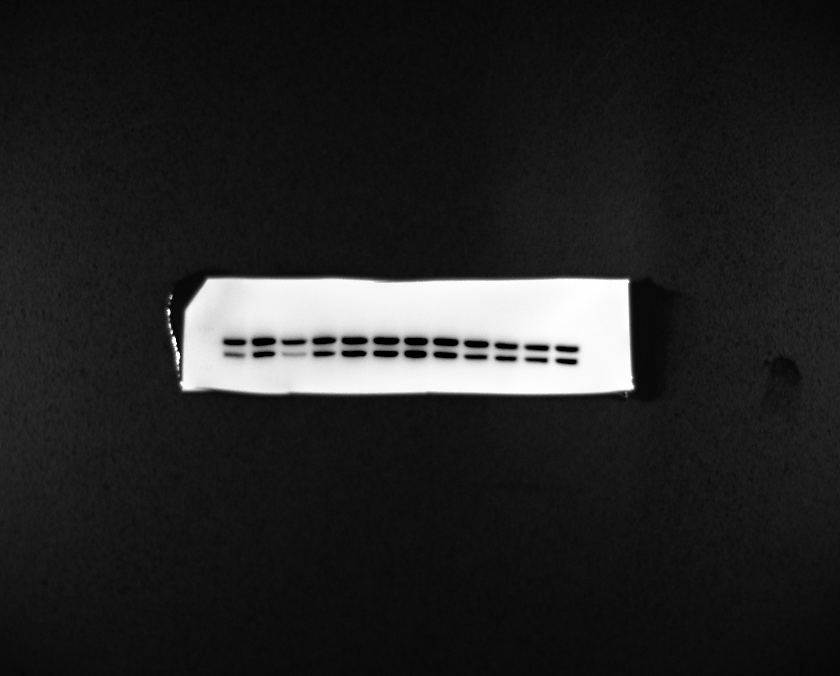

Supplement: Supplementary file 4 [file DataSheet4.ZIP › Data-figure5/western blot/WB image/ERK.tif]

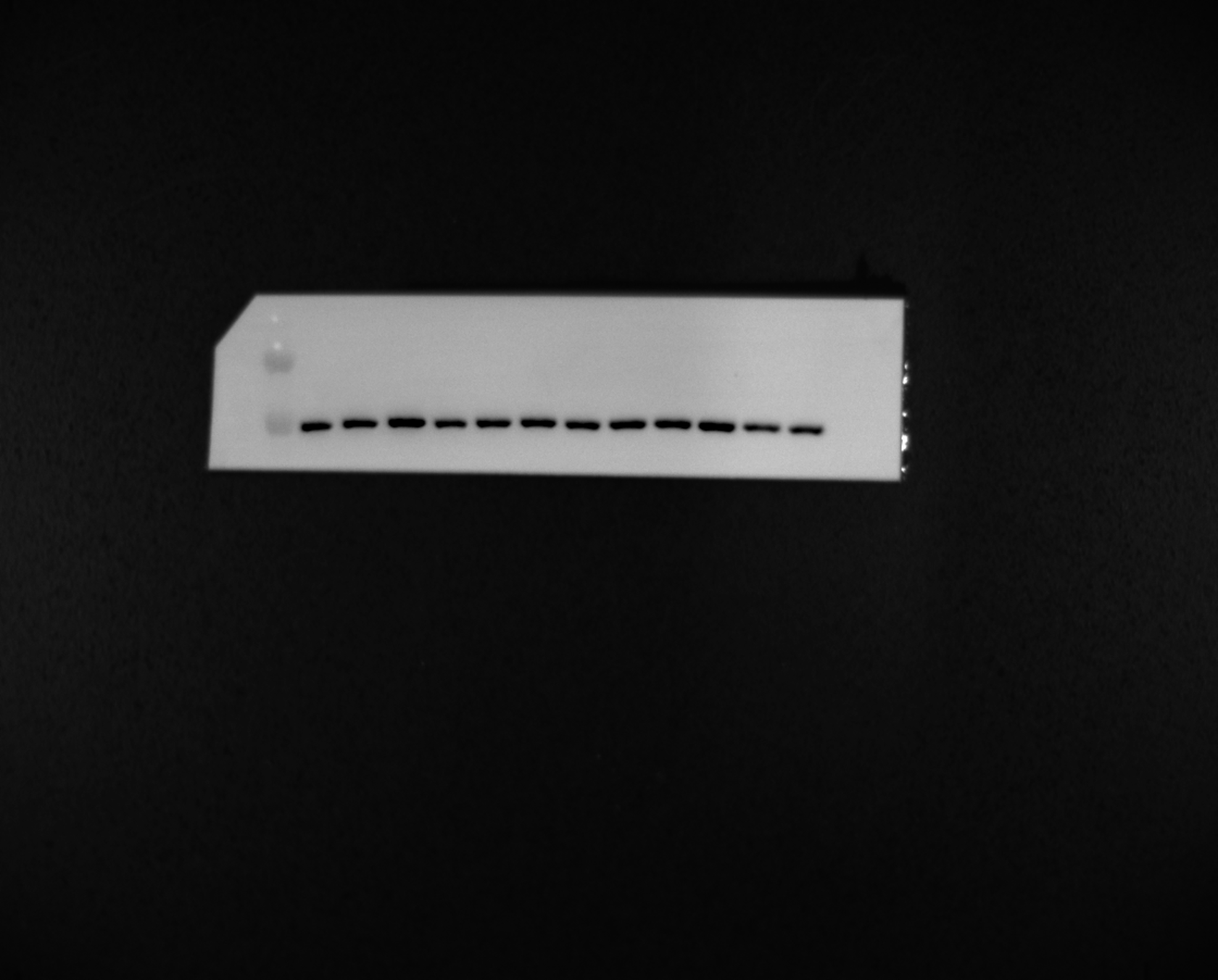

Supplement: Supplementary file 4 [file DataSheet4.ZIP › Data-figure5/western blot/WB image/GAPDH1.tiff]

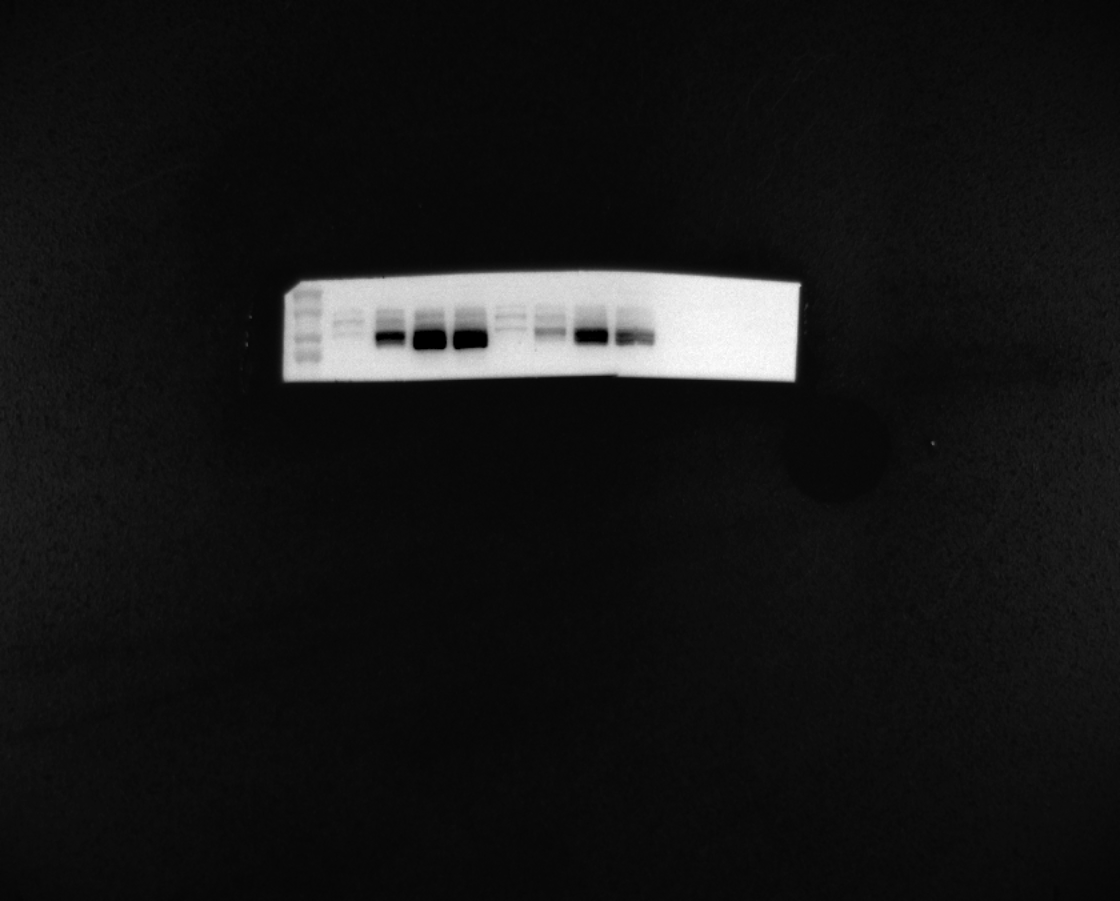

Supplement: Supplementary file 4 [file DataSheet4.ZIP › Data-figure5/western blot/WB image/NFATc1.tiff]

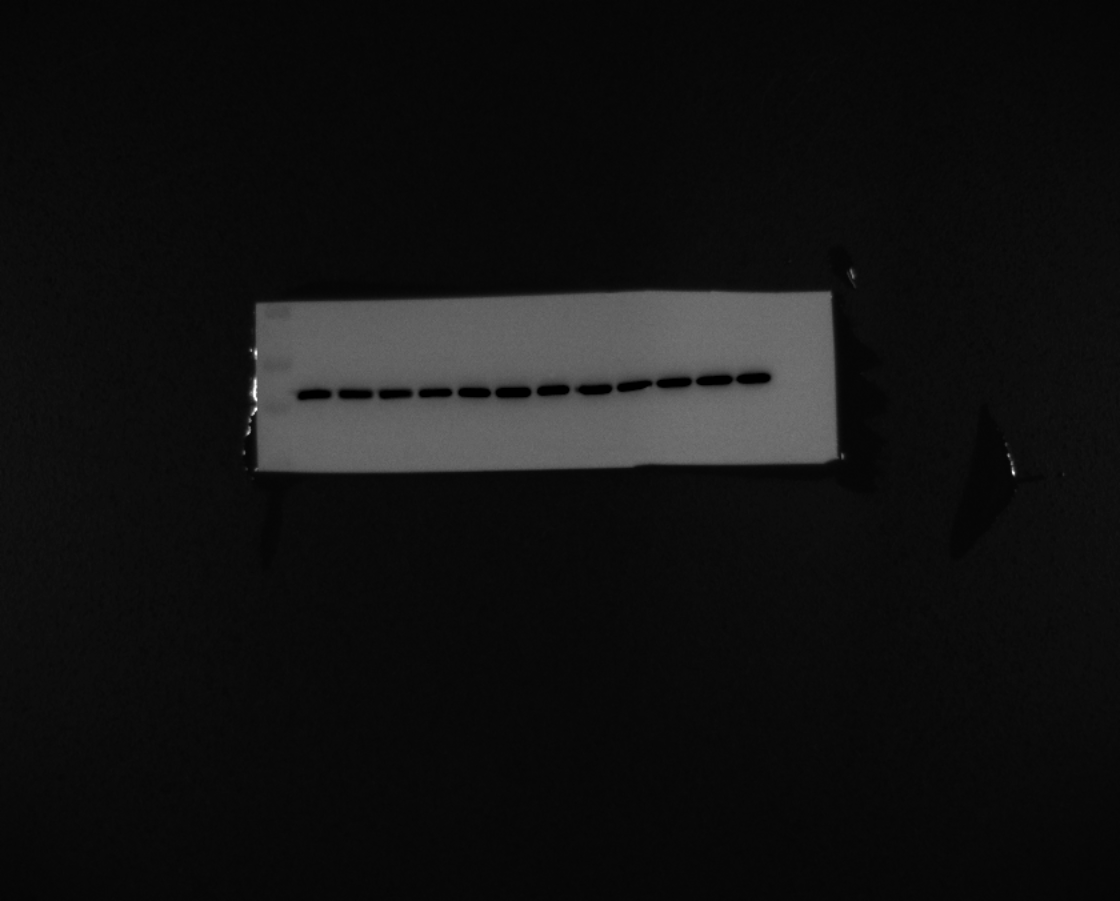

Supplement: Supplementary file 4 [file DataSheet4.ZIP › Data-figure5/western blot/WB image/p38.tiff]

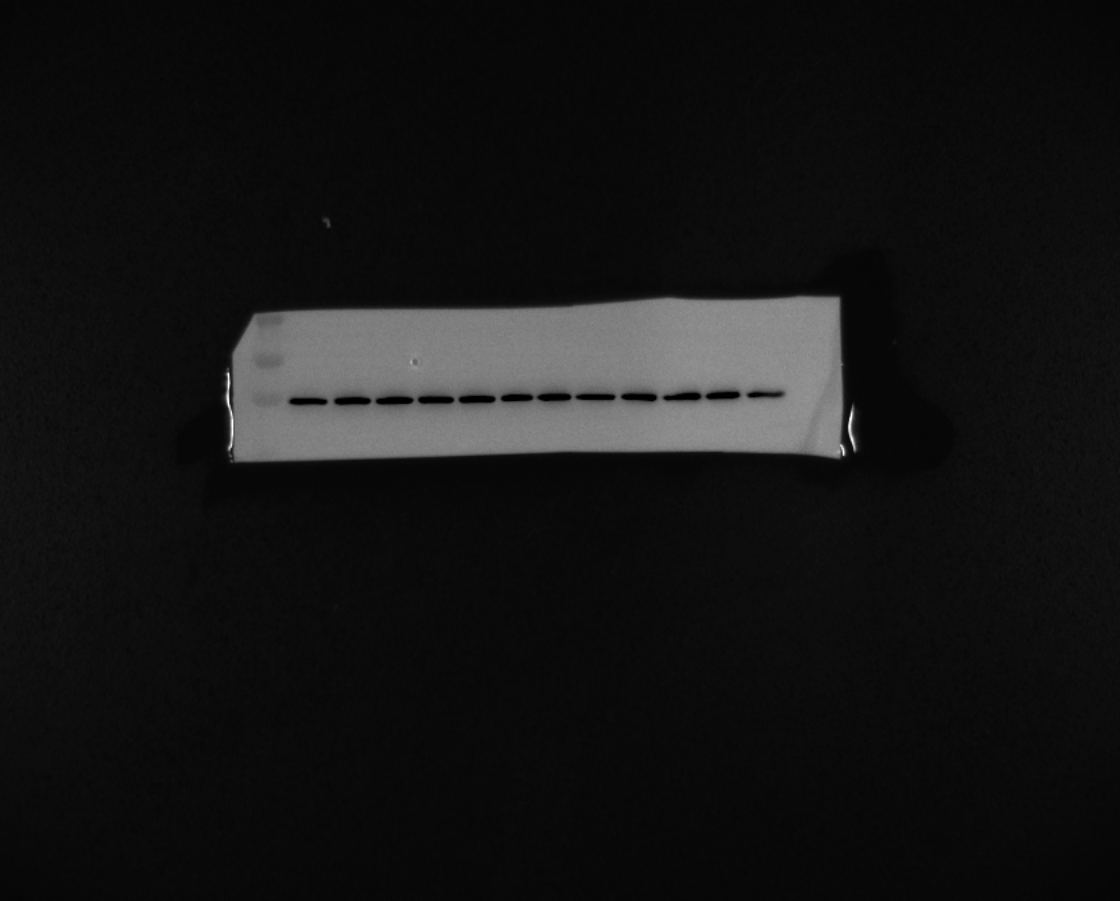

Supplement: Supplementary file 4 [file DataSheet4.ZIP › Data-figure5/western blot/WB image/GAPDH2.tiff]

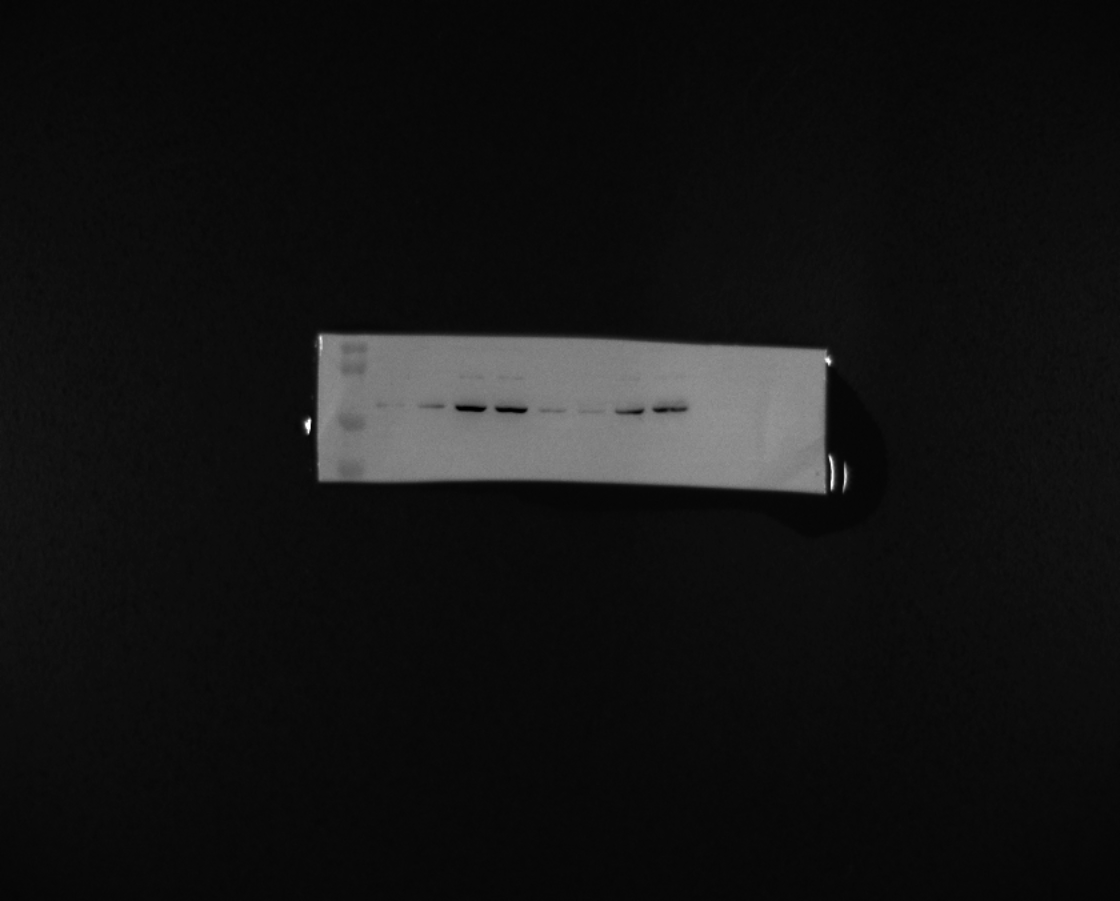

Supplement: Supplementary file 4 [file DataSheet4.ZIP › Data-figure5/western blot/WB image/TRAF6.tiff]

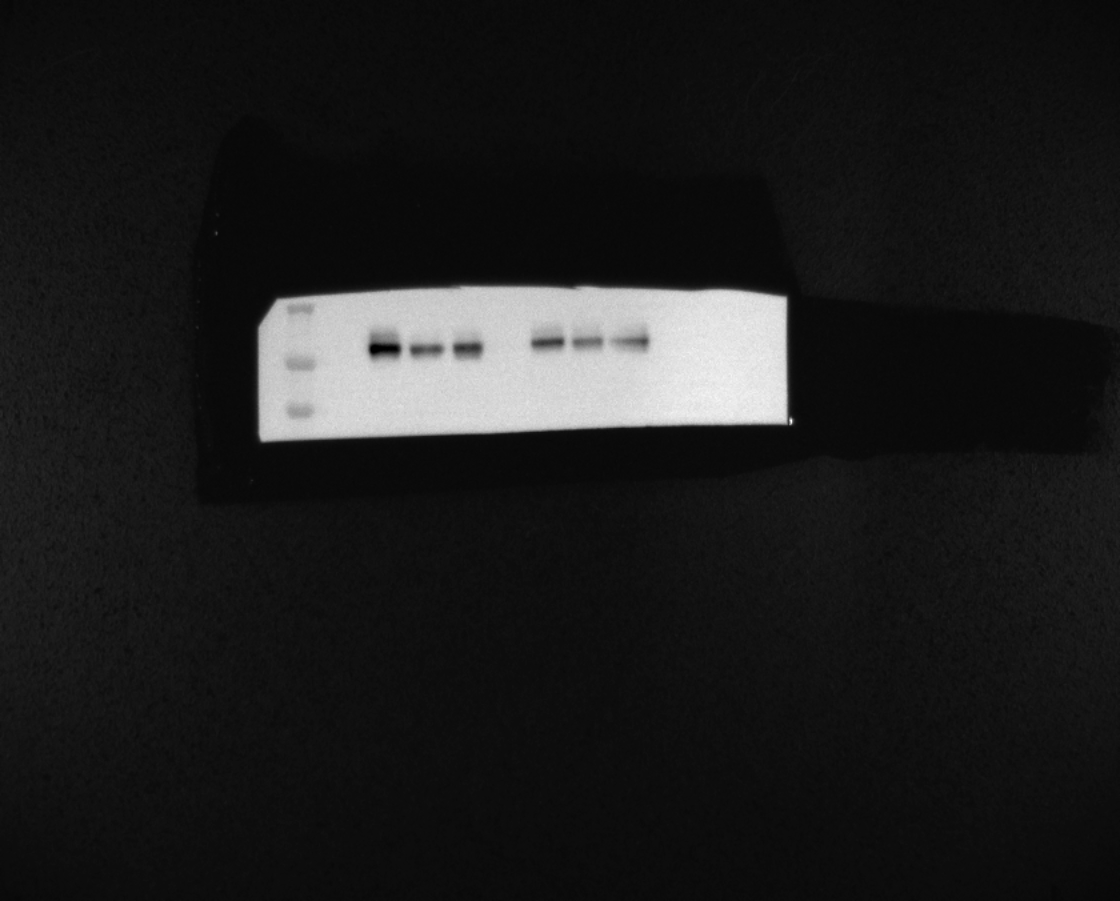

Supplement: Supplementary file 4 [file DataSheet4.ZIP › Data-figure5/western blot/WB image/c-FOS.tiff]

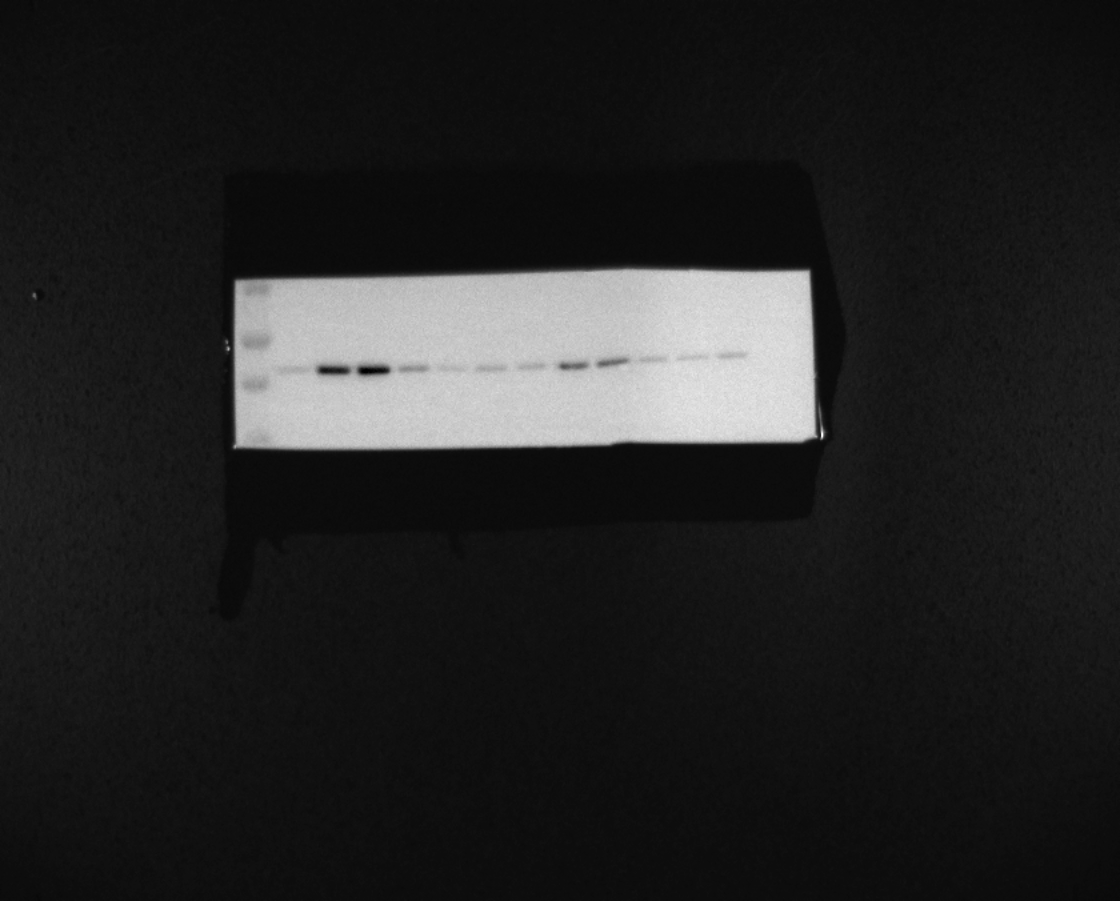

Supplement: Supplementary file 4 [file DataSheet4.ZIP › Data-figure5/western blot/WB image/pp38.tiff]

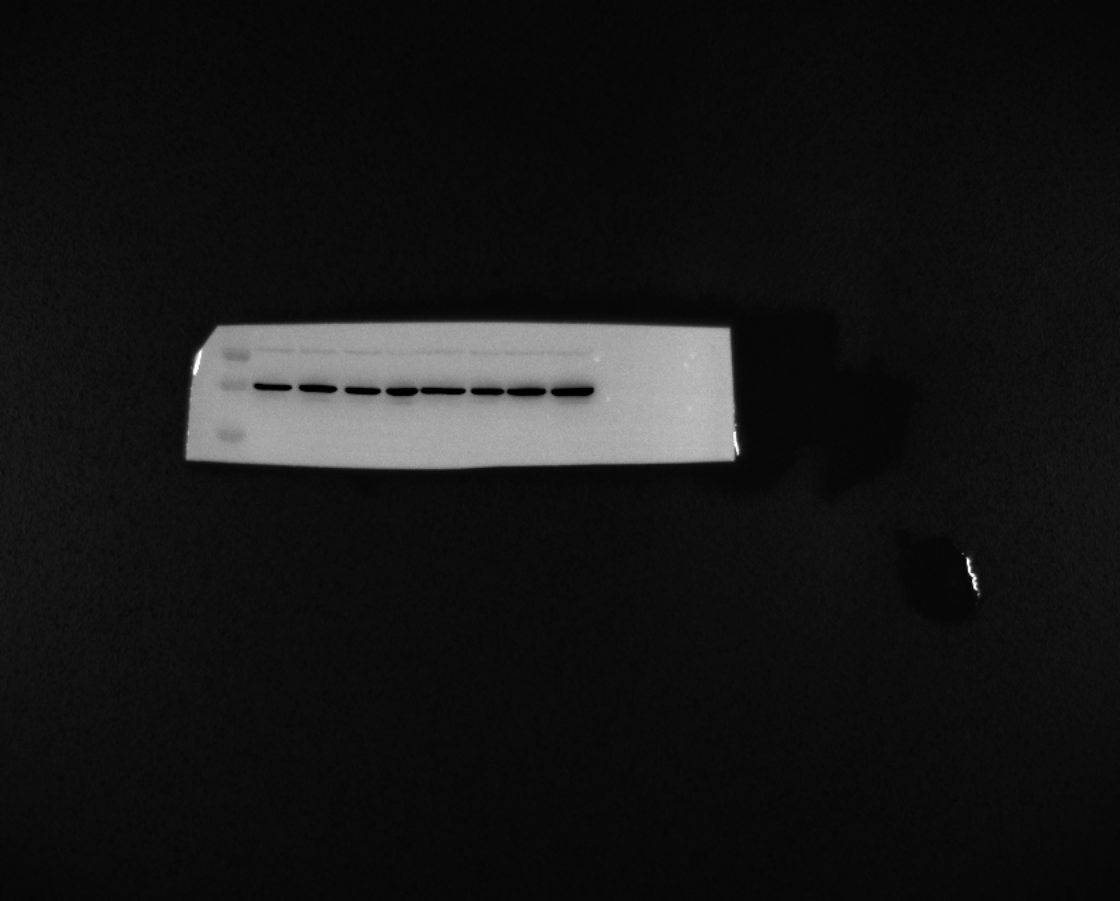

Supplement: Supplementary file 4 [file DataSheet4.ZIP › Data-figure5/western blot/WB image/GAPDH3.tiff]

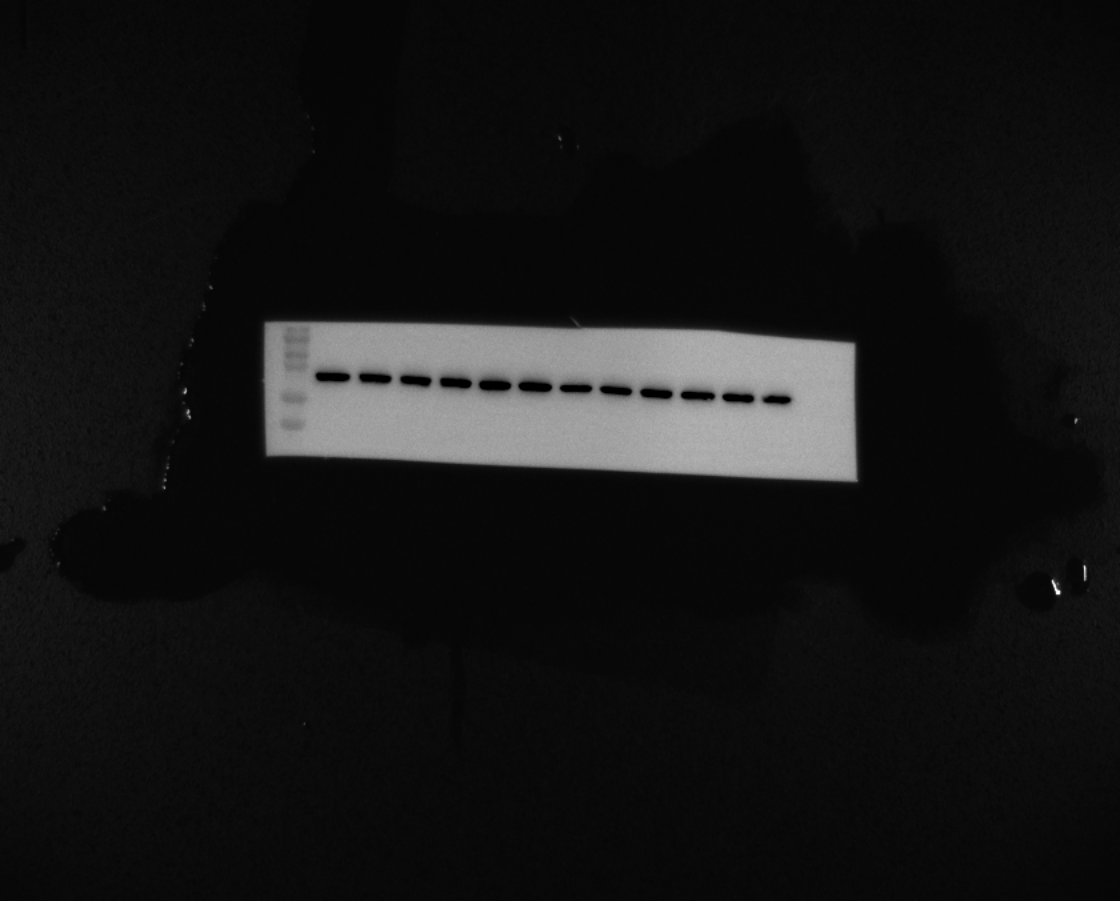

Supplement: Supplementary file 4 [file DataSheet4.ZIP › Data-figure5/western blot/WB image/p65.tiff]

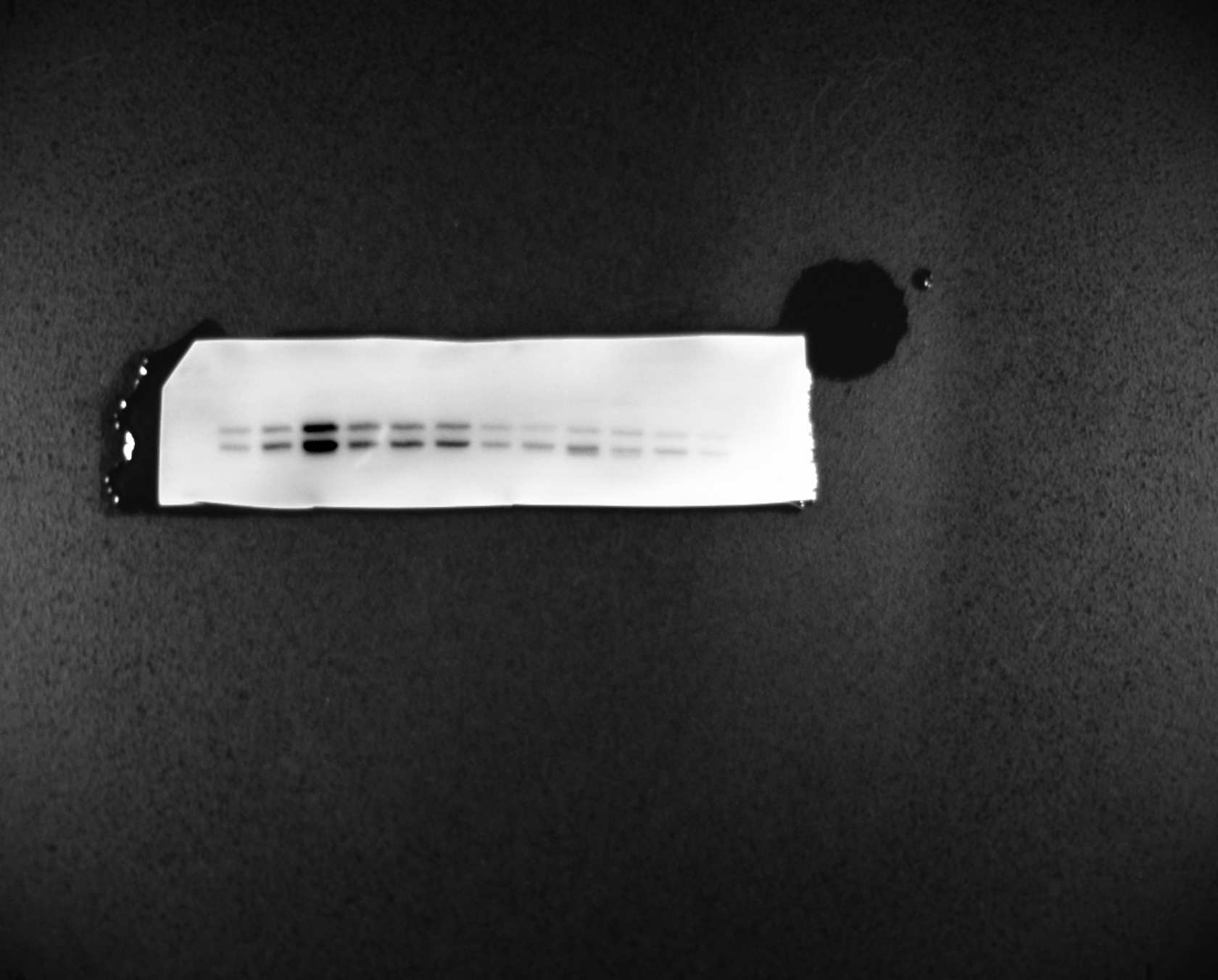

Supplement: Supplementary file 4 [file DataSheet4.ZIP › Data-figure5/western blot/WB image/perk.tiff]

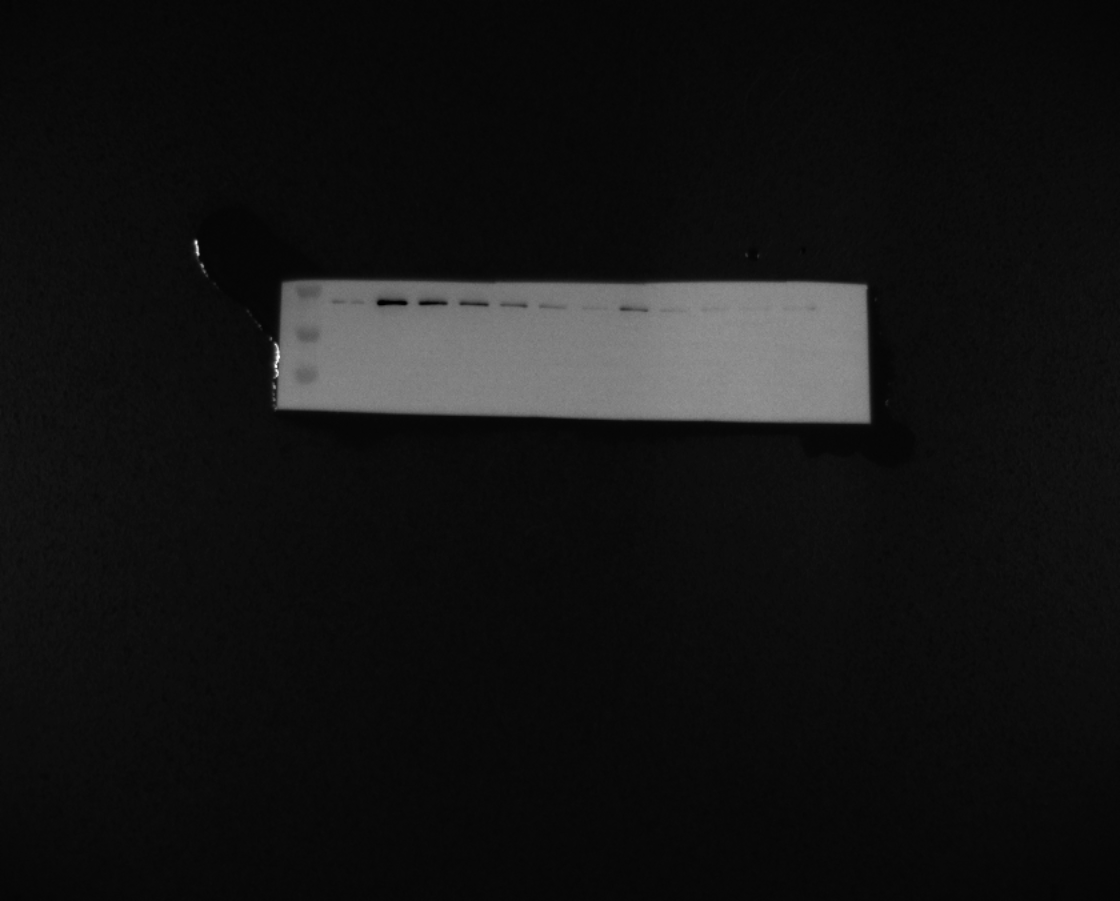

Supplement: Supplementary file 4 [file DataSheet4.ZIP › Data-figure5/western blot/WB image/pp65.tiff]

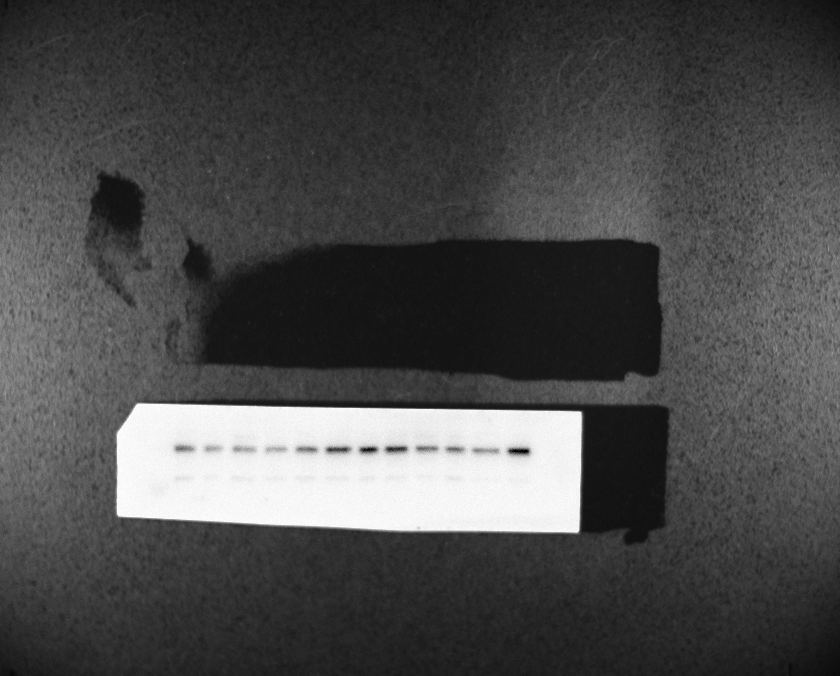

Supplement: Supplementary file 4 [file DataSheet4.ZIP › Data-figure5/western blot/WB image/I╬║B╬▒.tif]

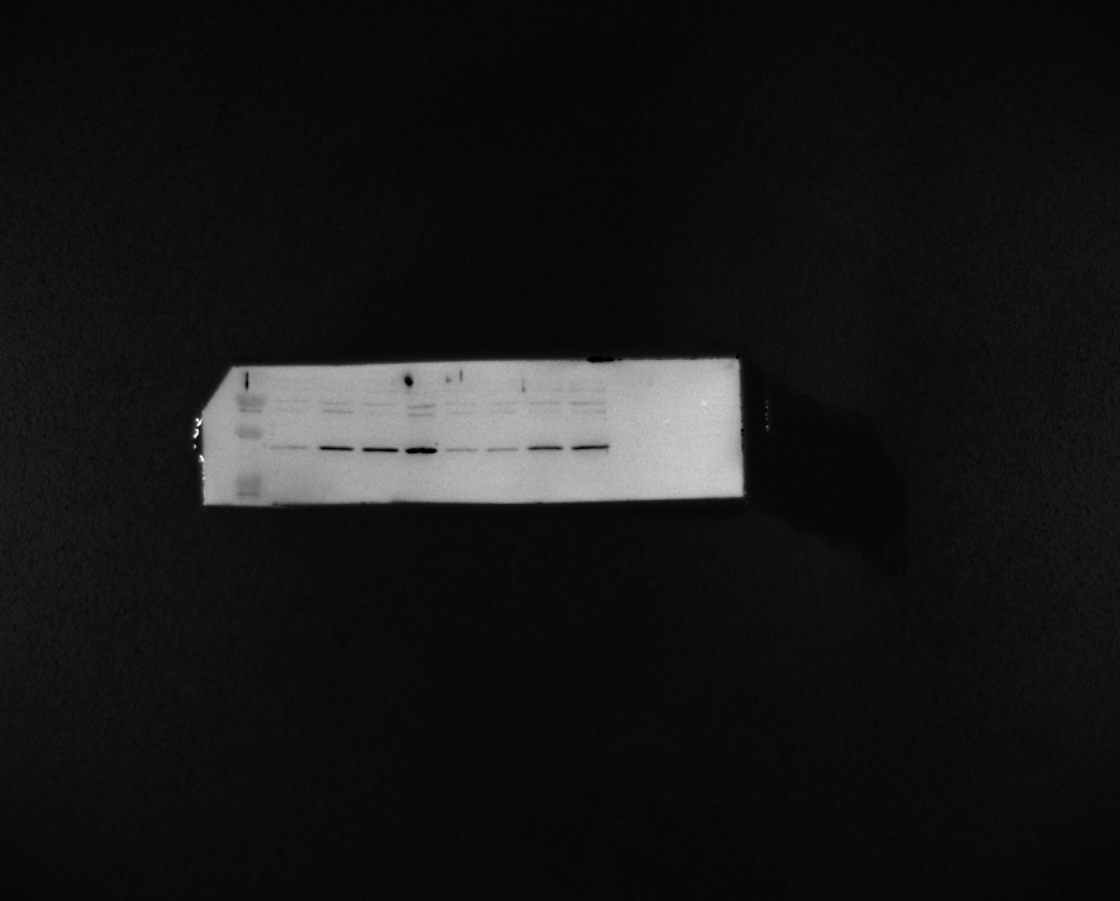

Supplement: Supplementary file 4 [file DataSheet4.ZIP › Data-figure5/western blot/WB image/siglec-15.tiff]

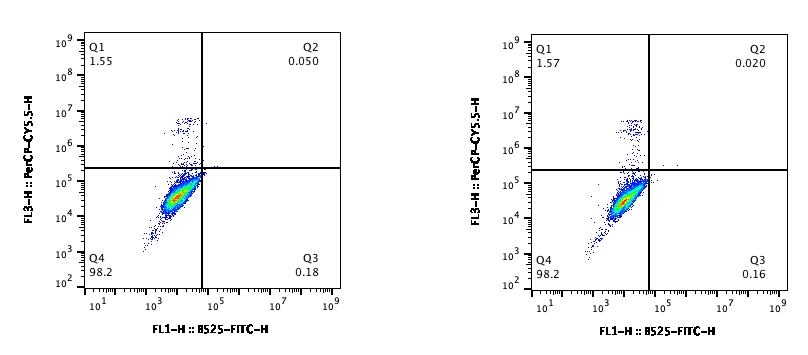

Supplement: Supplementary file 5 [file DataSheet1.ZIP › Data-figure1/flow cytometry/15-Aug-2021-Layout.jpg]

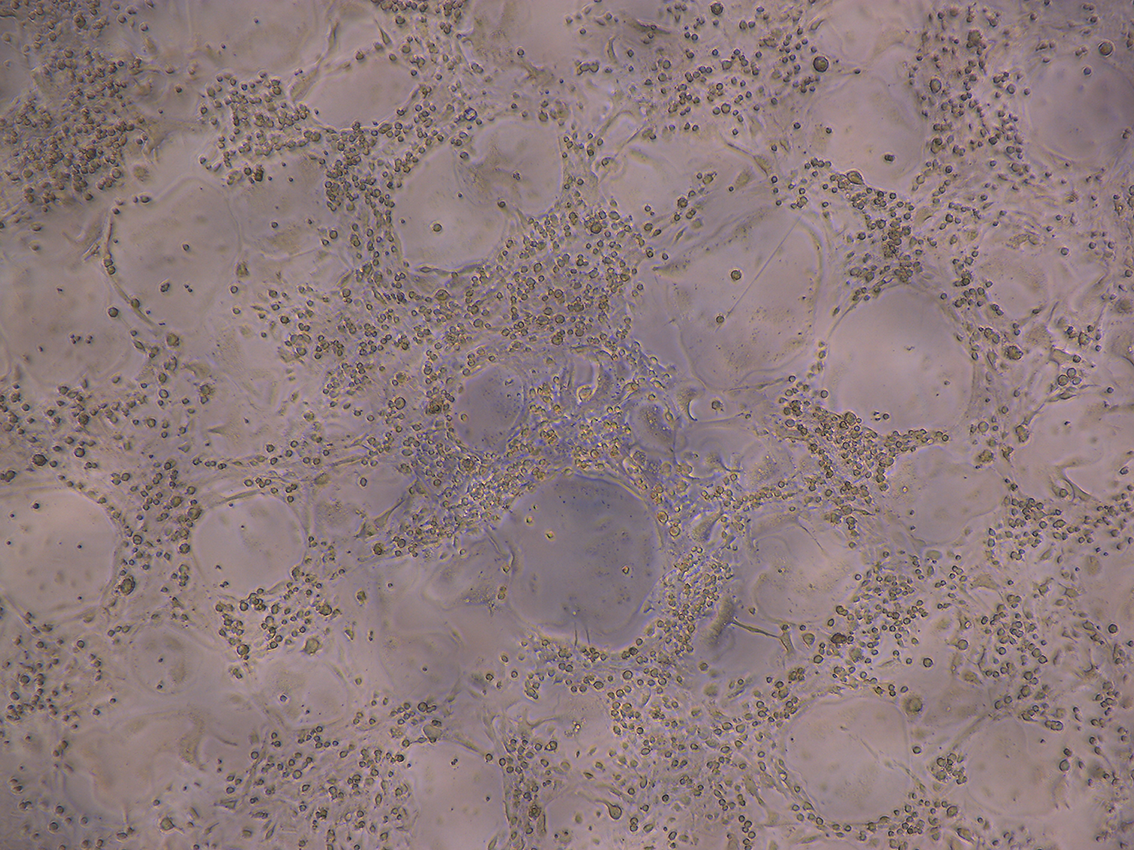

Supplement: Supplementary file 6 [file DataSheet10.ZIP › supplementary image data(2)/D/0uM'.tif]

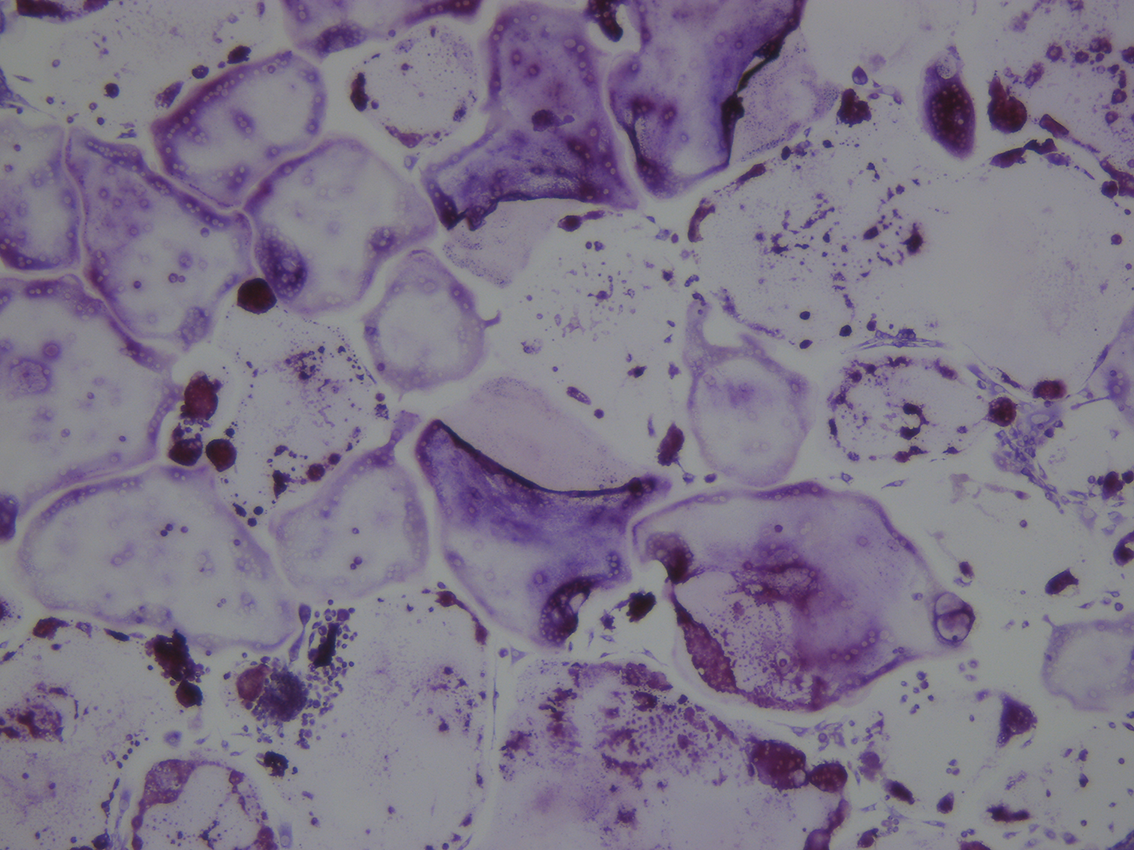

Supplement: Supplementary file 6 [file DataSheet10.ZIP › supplementary image data(2)/D/10uM.tif]

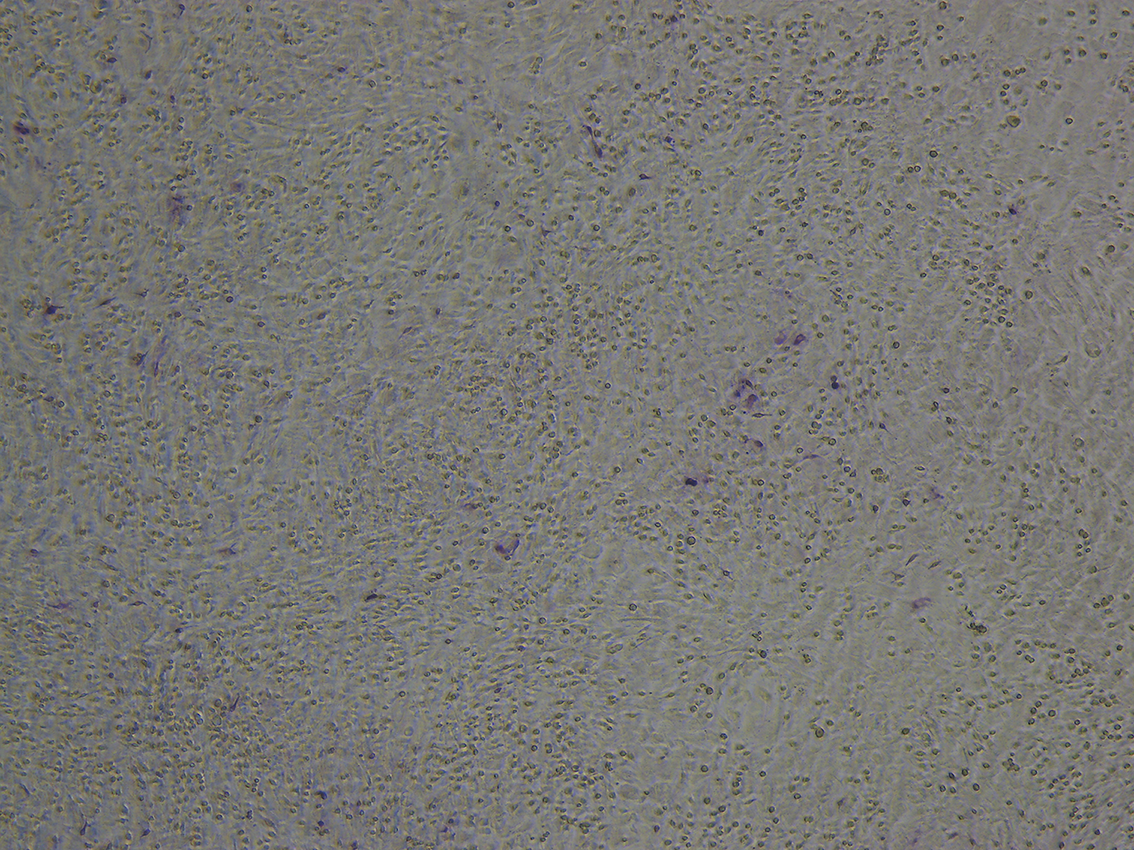

Supplement: Supplementary file 6 [file DataSheet10.ZIP › supplementary image data(2)/D/(-).tif]

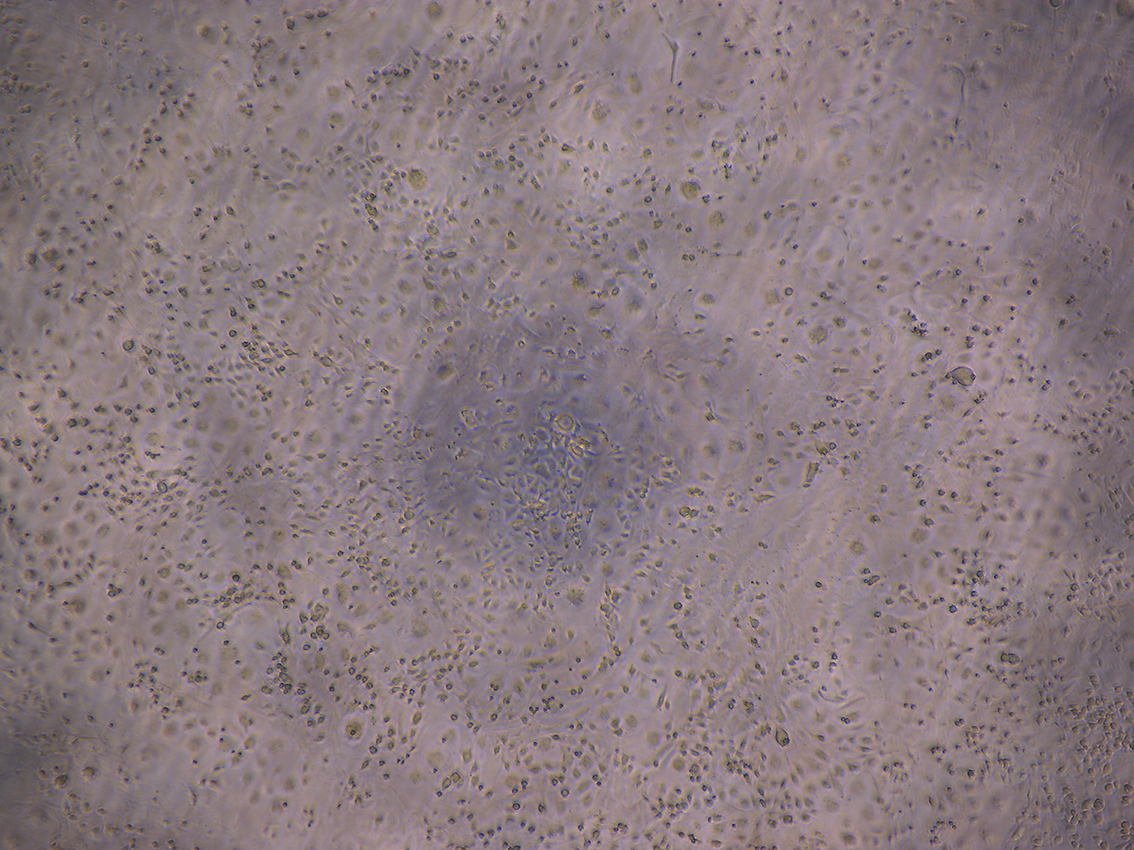

Supplement: Supplementary file 6 [file DataSheet10.ZIP › supplementary image data(2)/D/(-)-1.tif]

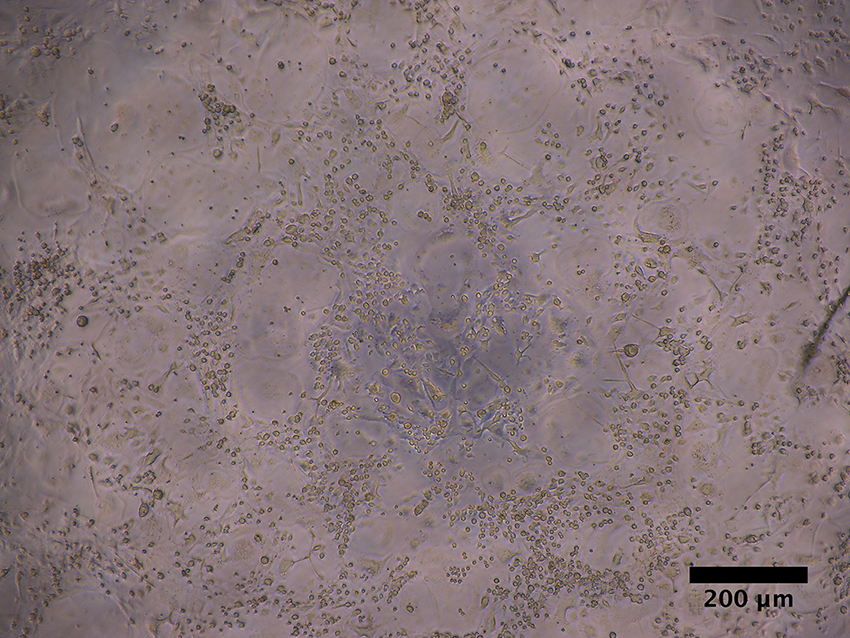

Supplement: Supplementary file 6 [file DataSheet10.ZIP › supplementary image data(2)/D/20uMΓÇÿ.tif]

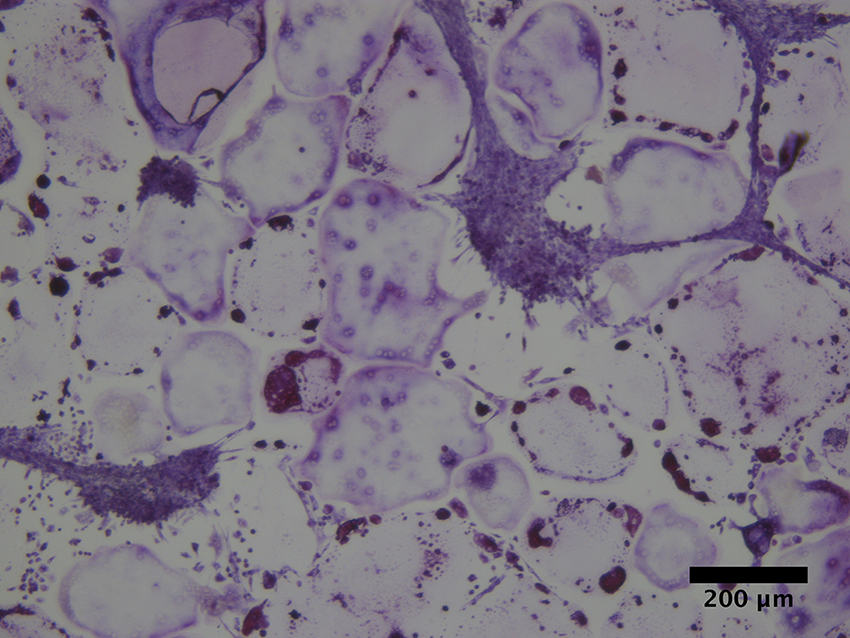

Supplement: Supplementary file 6 [file DataSheet10.ZIP › supplementary image data(2)/D/20uM.tif]

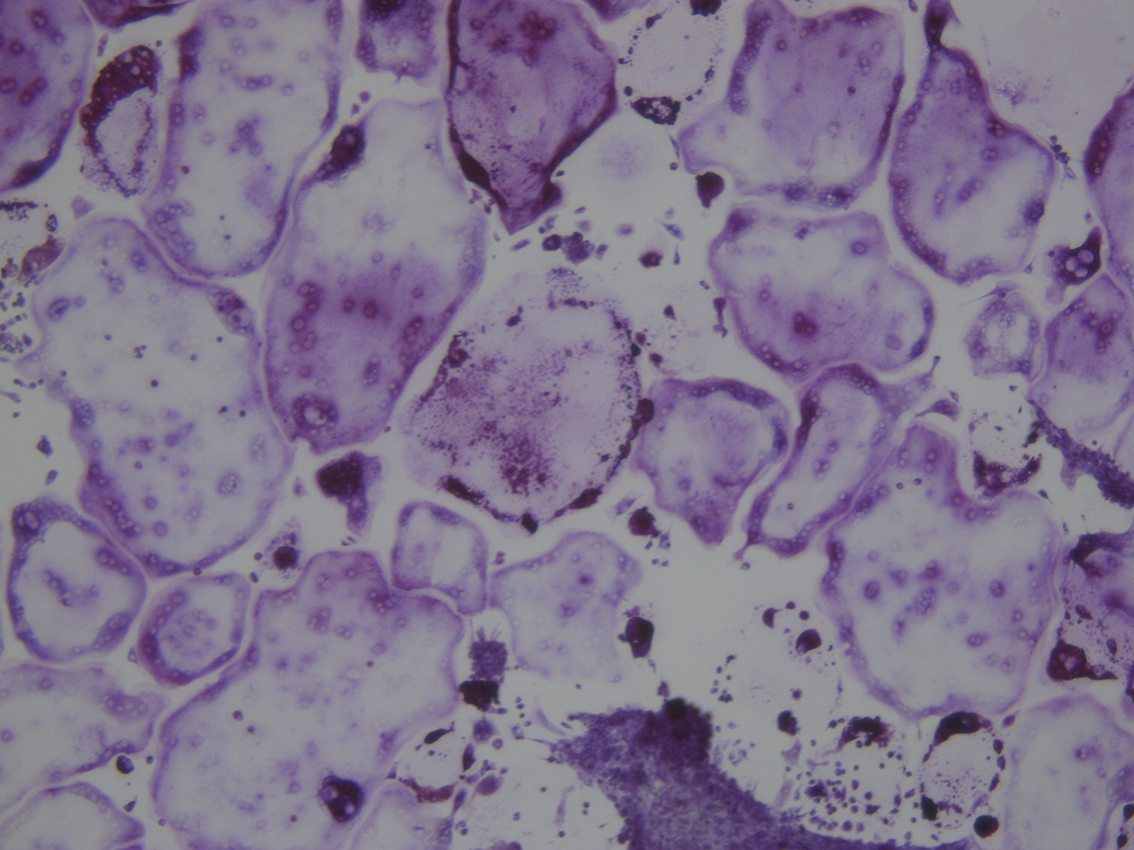

Supplement: Supplementary file 6 [file DataSheet10.ZIP › supplementary image data(2)/D/5uM.tif]

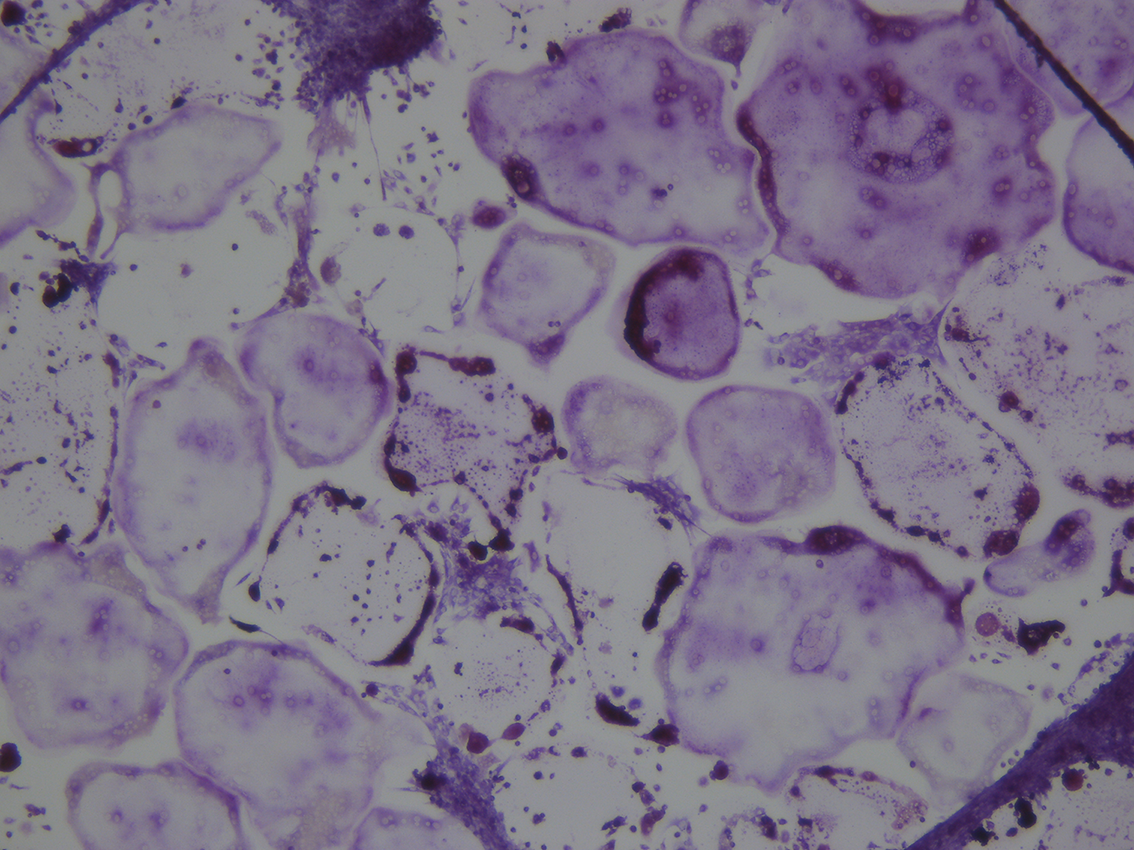

Supplement: Supplementary file 6 [file DataSheet10.ZIP › supplementary image data(2)/D/0uM.tif]

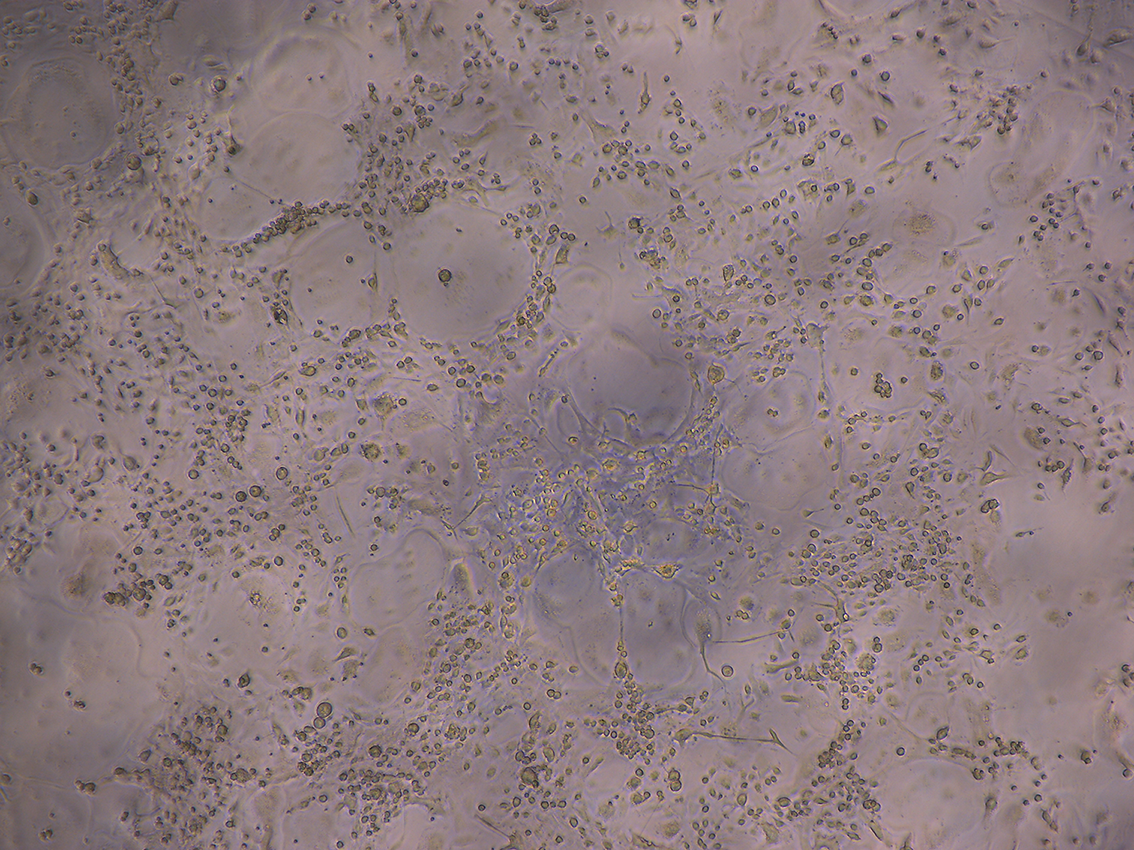

Supplement: Supplementary file 6 [file DataSheet10.ZIP › supplementary image data(2)/D/10uM'.tif]

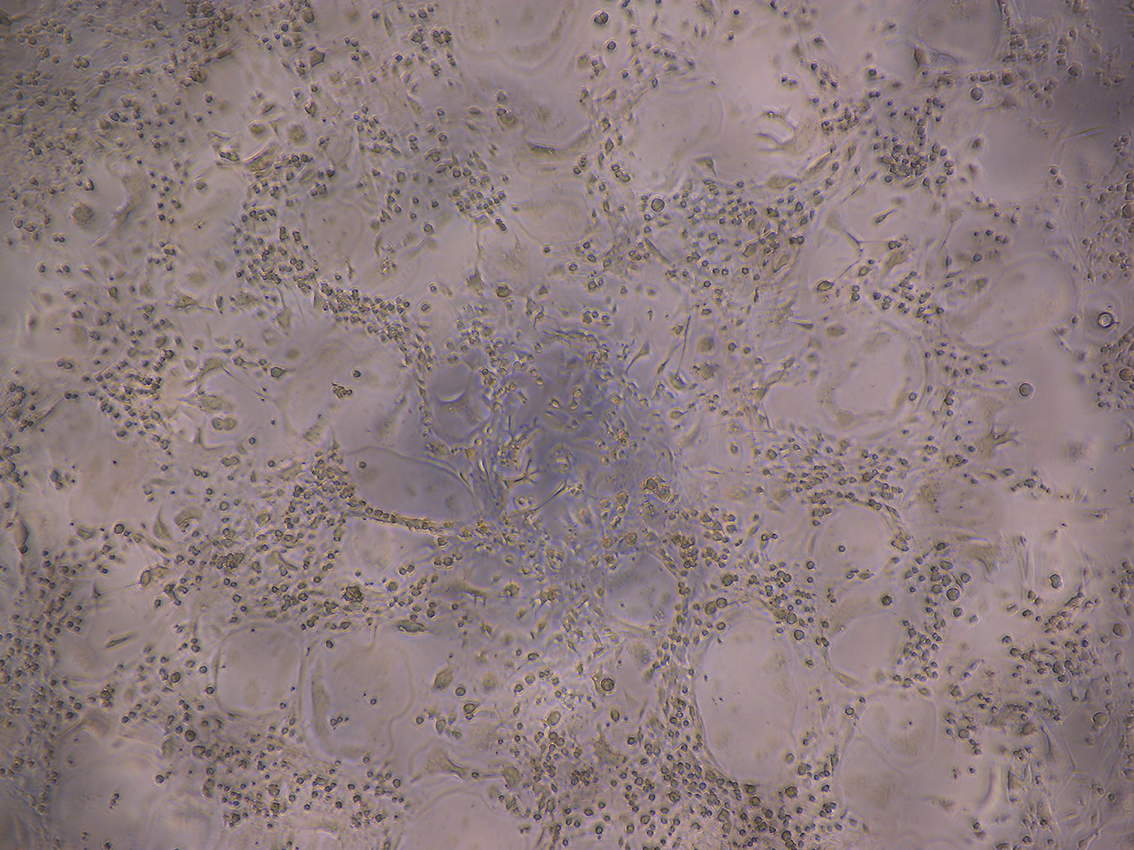

Supplement: Supplementary file 6 [file DataSheet10.ZIP › supplementary image data(2)/D/5uM'.tif]

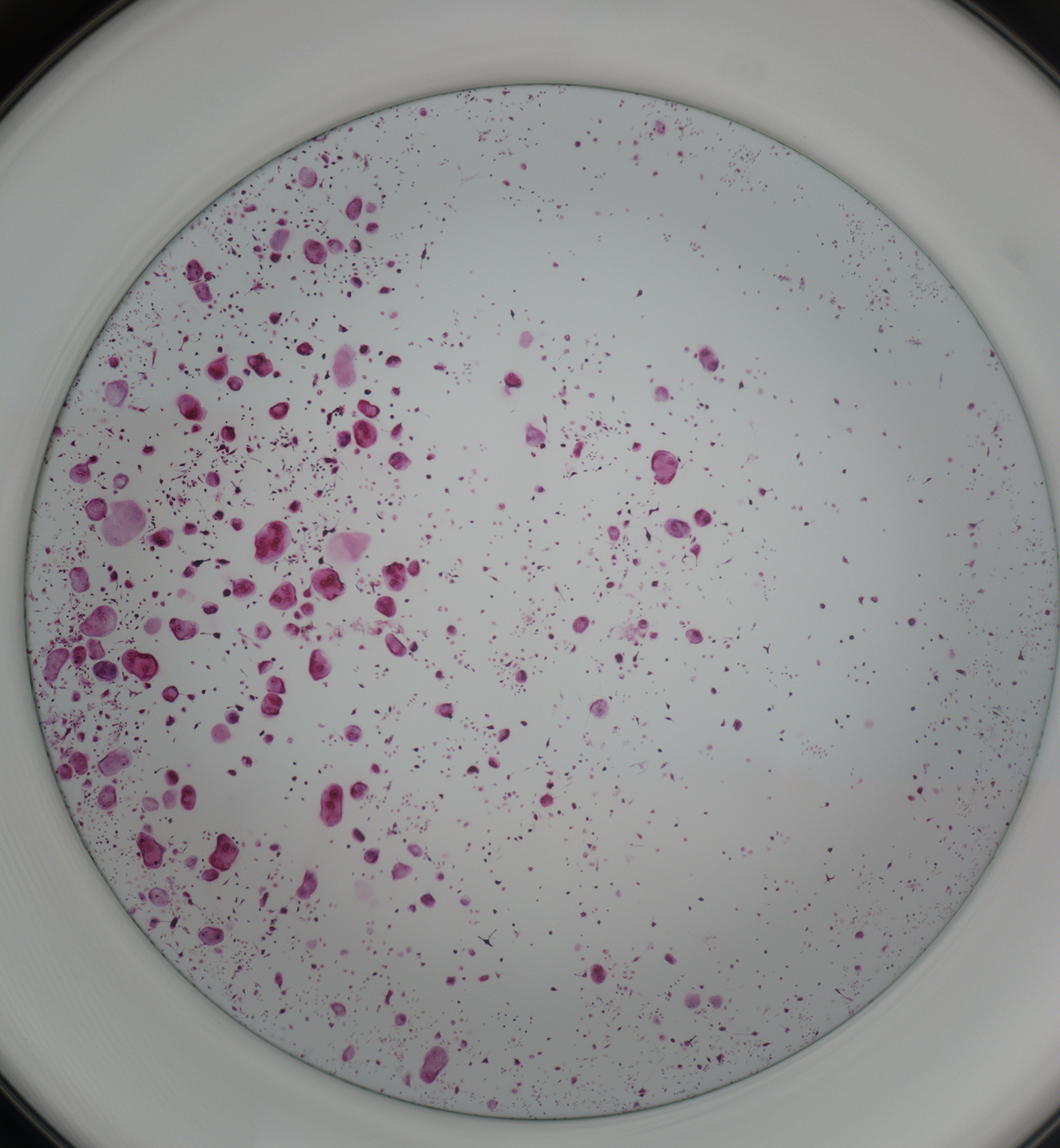

Supplement: Supplementary file 7 [file DataSheet6.ZIP › figure 2 data 1/dose/10.tif]

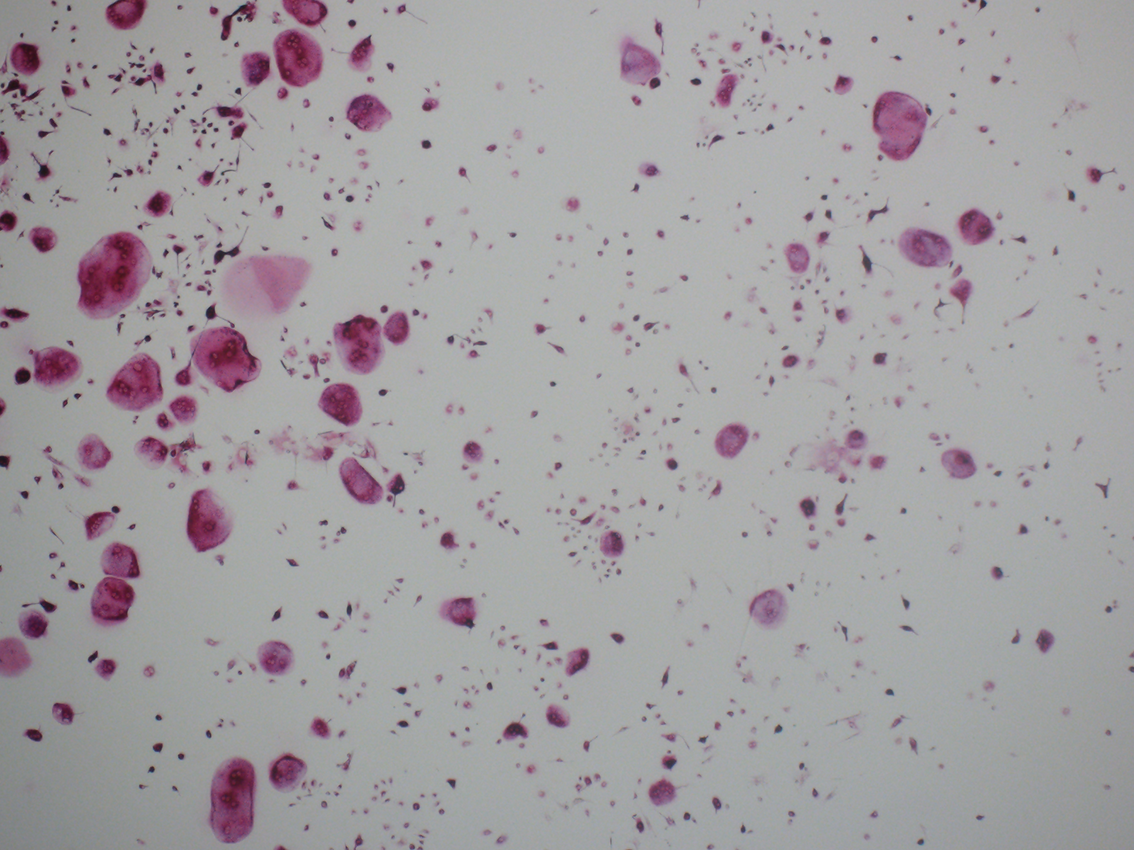

Supplement: Supplementary file 7 [file DataSheet6.ZIP › figure 2 data 1/dose/10-1.tif]

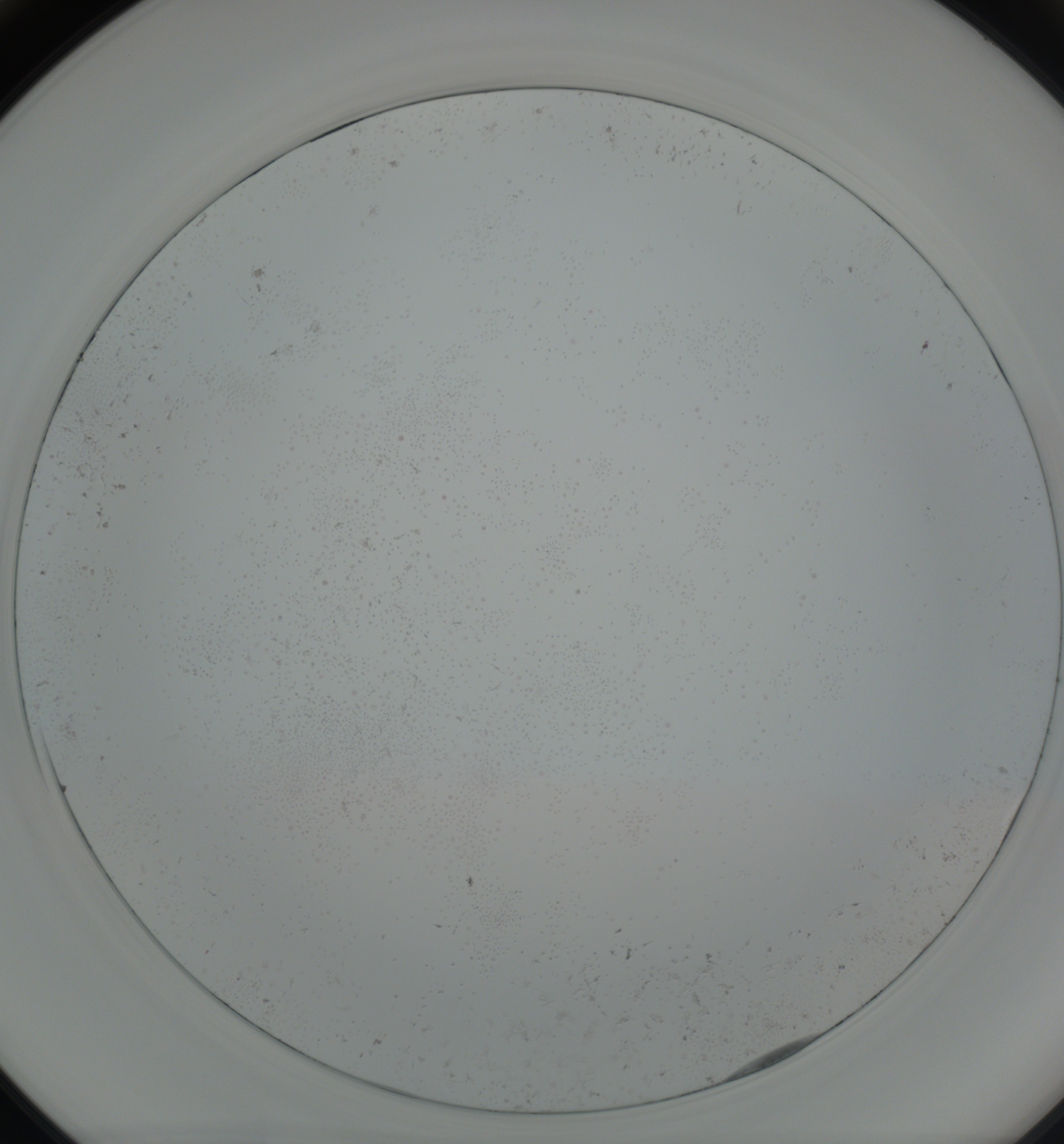

Supplement: Supplementary file 7 [file DataSheet6.ZIP › figure 2 data 1/dose/(-).tif]

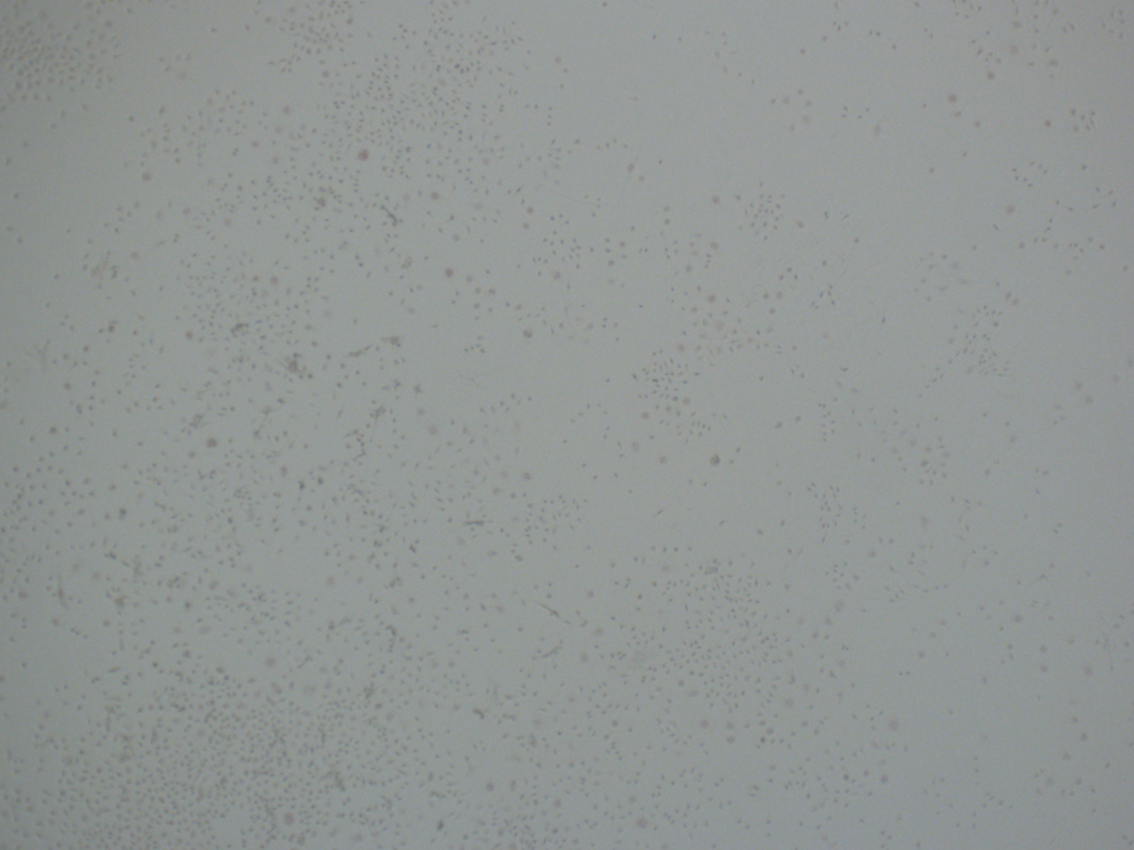

Supplement: Supplementary file 7 [file DataSheet6.ZIP › figure 2 data 1/dose/(-)-1.tif]

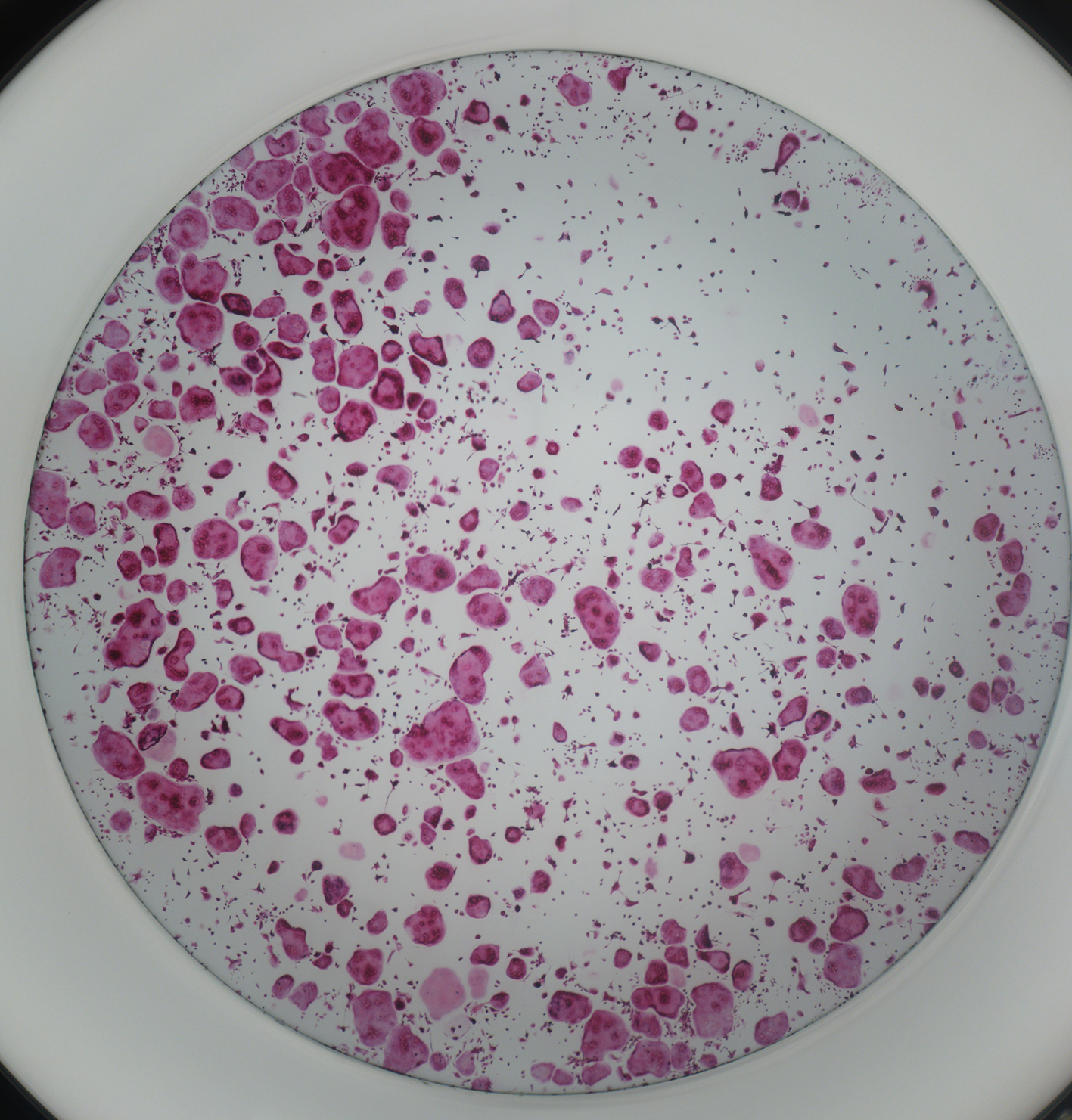

Supplement: Supplementary file 7 [file DataSheet6.ZIP › figure 2 data 1/dose/0.tif]

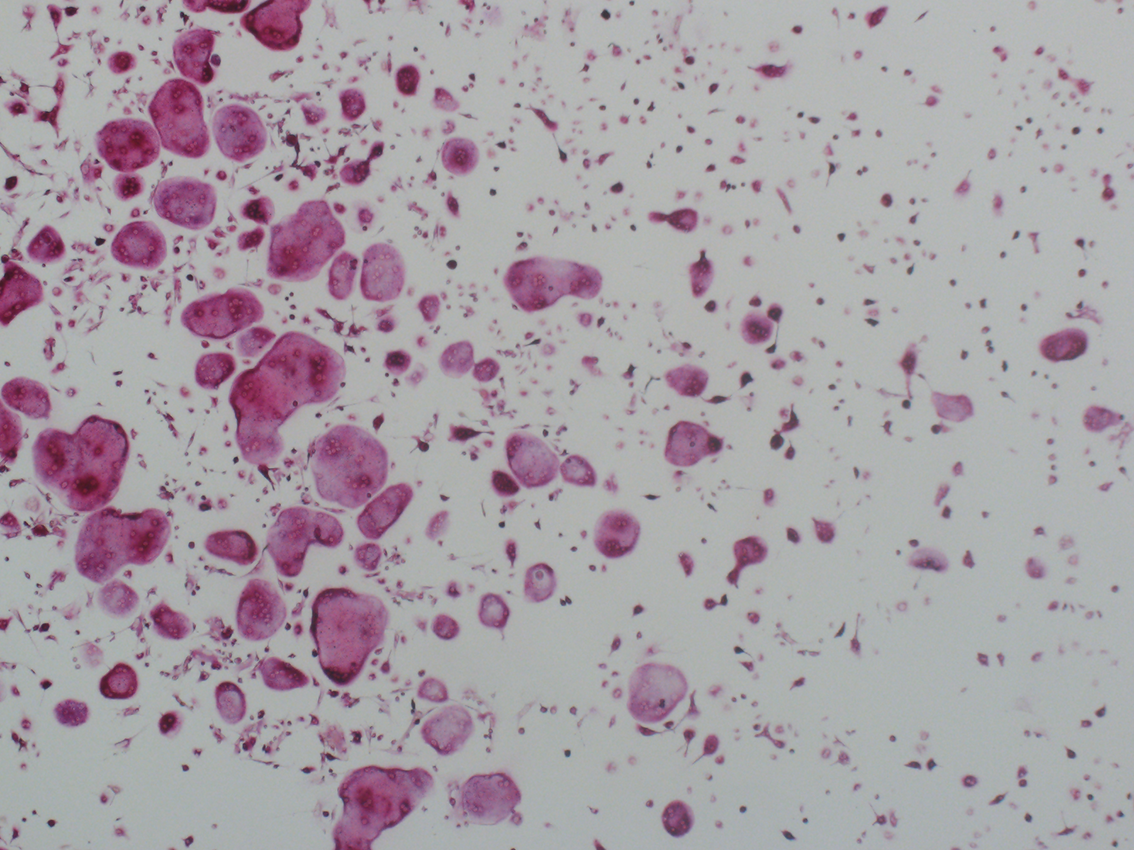

Supplement: Supplementary file 7 [file DataSheet6.ZIP › figure 2 data 1/dose/5-1.tif]

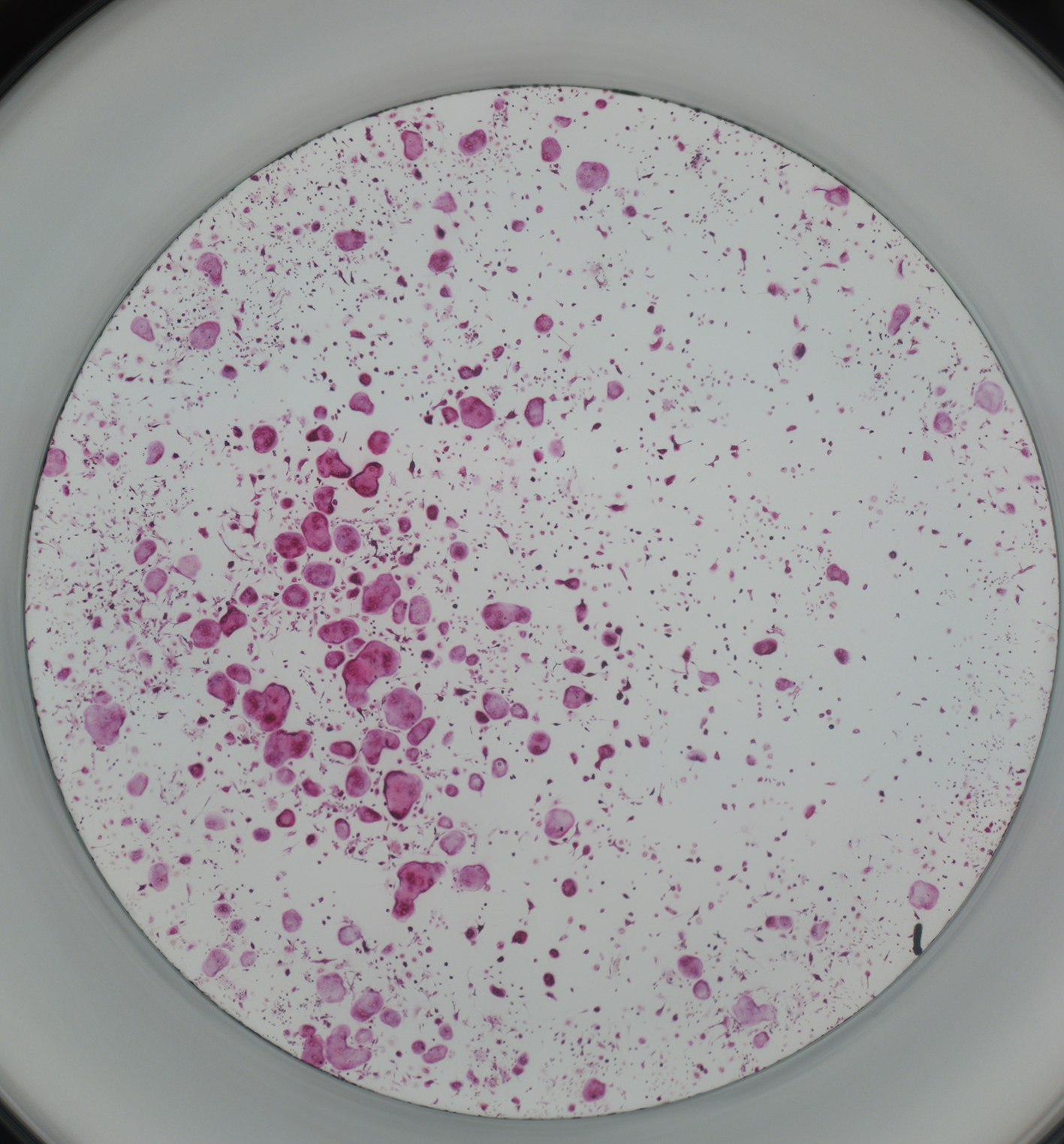

Supplement: Supplementary file 7 [file DataSheet6.ZIP › figure 2 data 1/dose/5.tif]

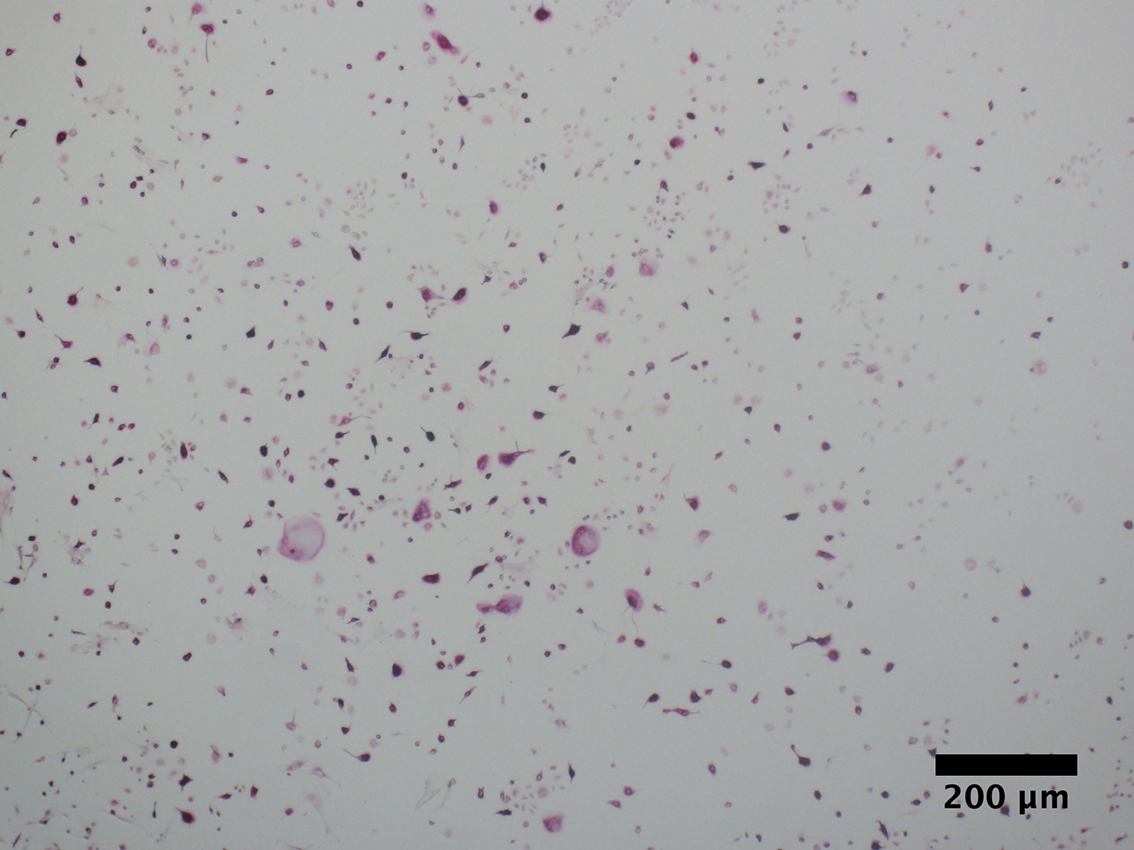

Supplement: Supplementary file 7 [file DataSheet6.ZIP › figure 2 data 1/dose/20-1.tif]

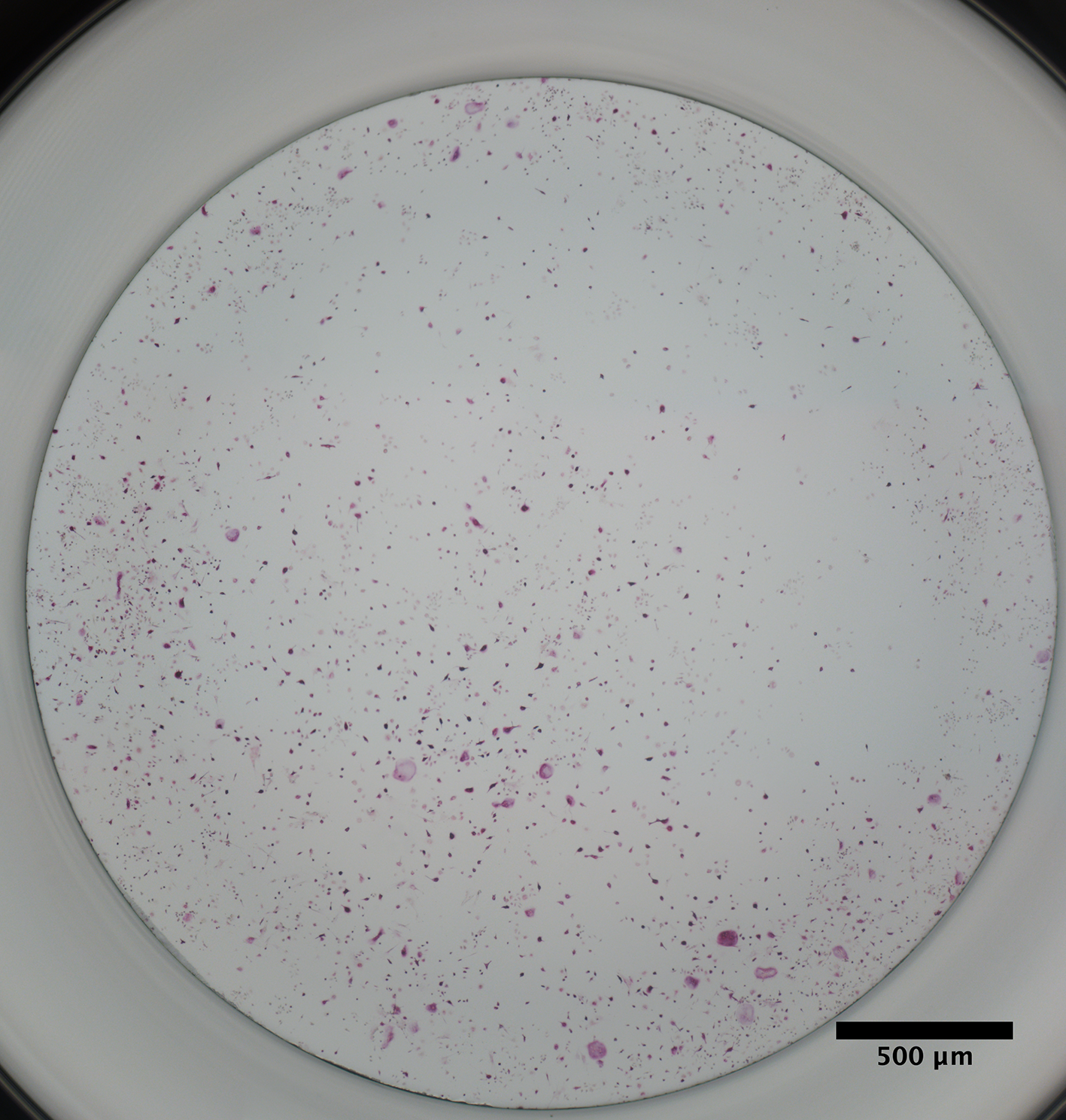

Supplement: Supplementary file 7 [file DataSheet6.ZIP › figure 2 data 1/dose/20.tif]

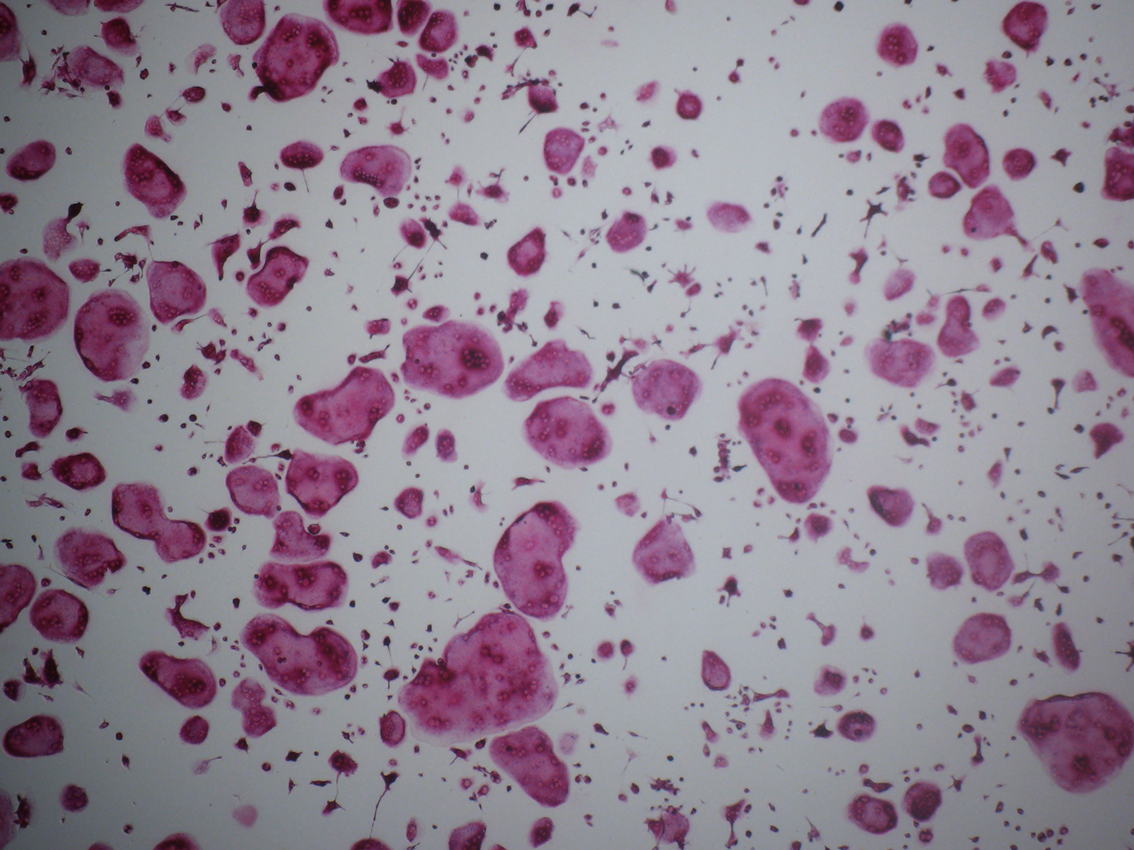

Supplement: Supplementary file 7 [file DataSheet6.ZIP › figure 2 data 1/dose/0-1.tif]

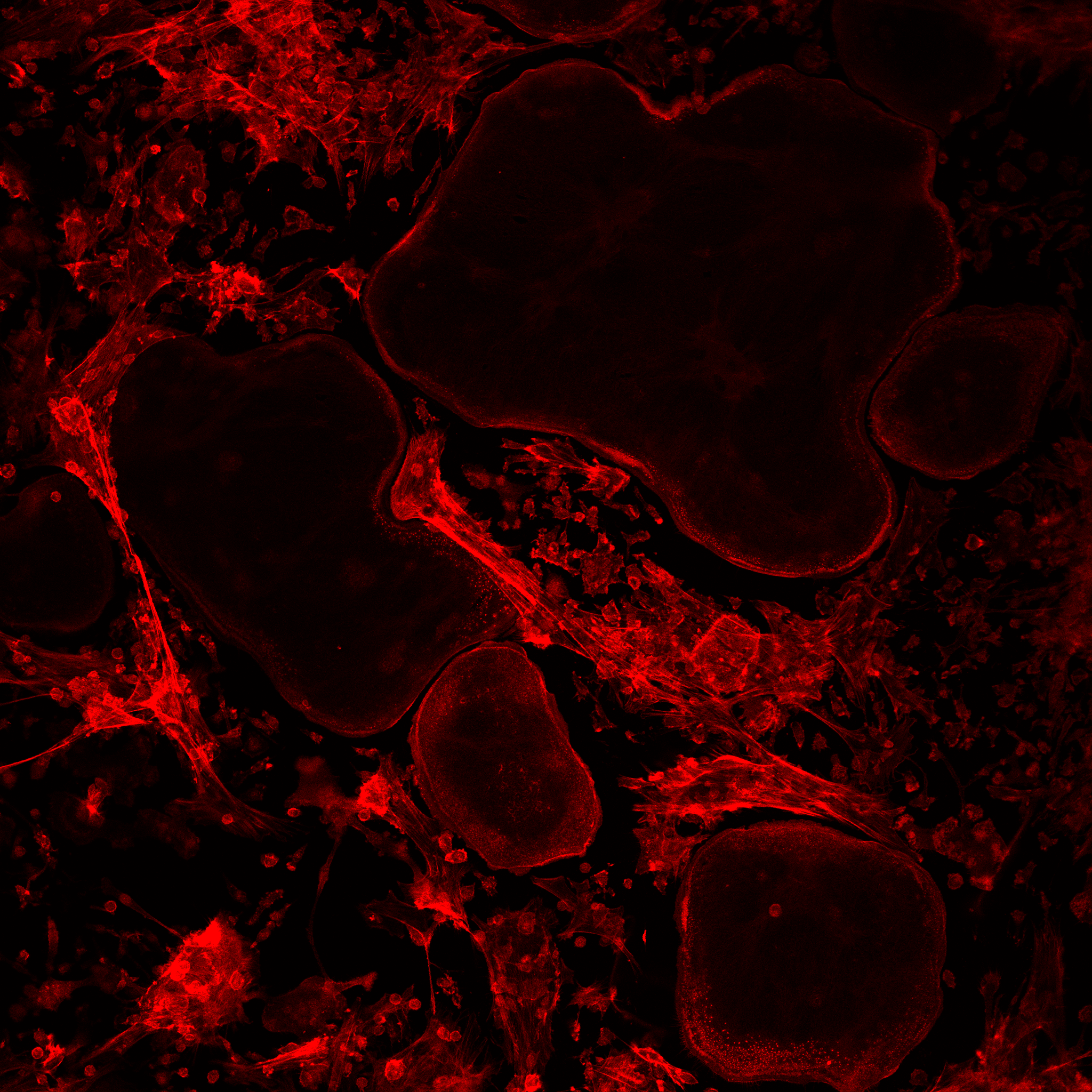

Supplement: Supplementary file 8 [file DataSheet2.ZIP › Data-figure3/F-actin/0uM f-actin.tif]

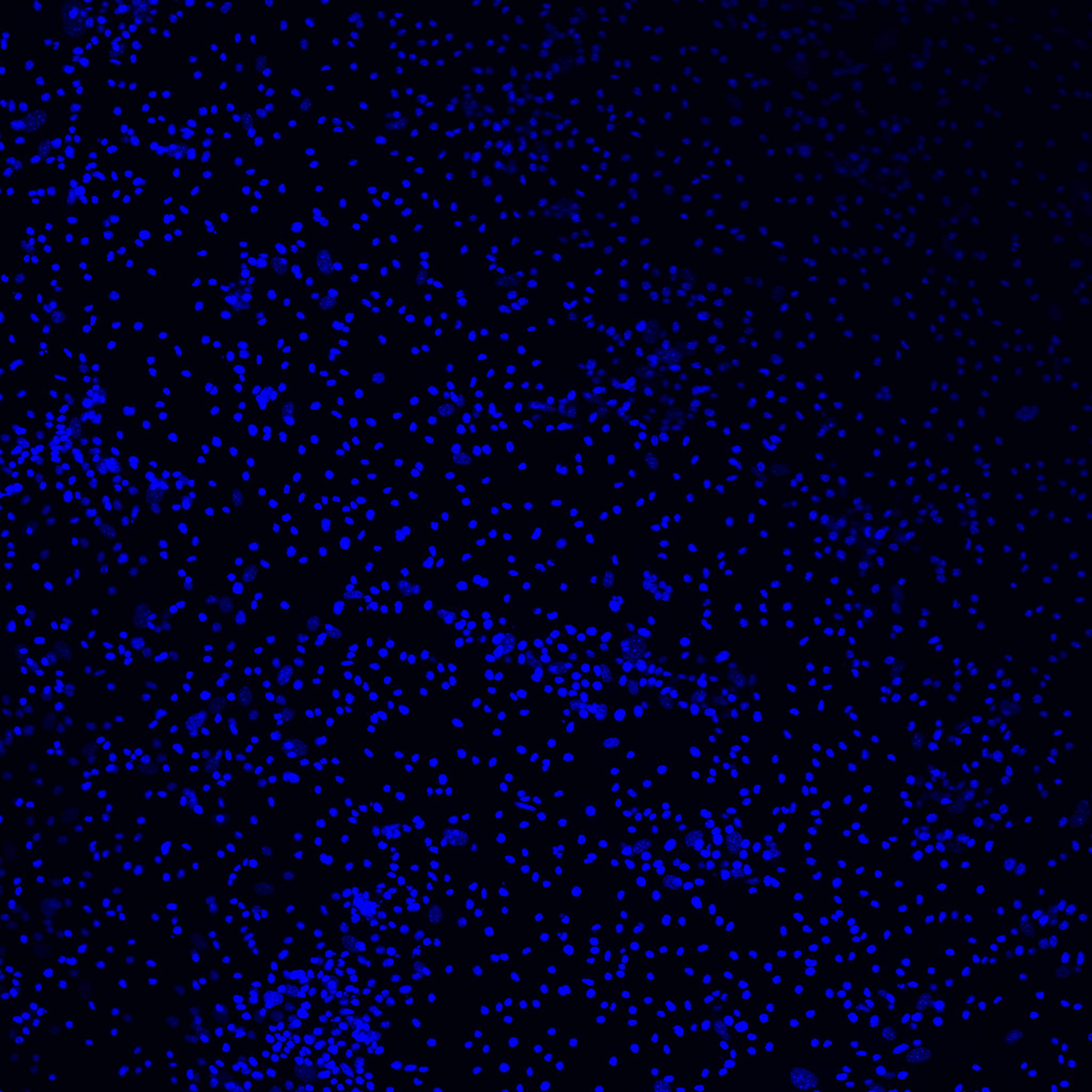

Supplement: Supplementary file 8 [file DataSheet2.ZIP › Data-figure3/F-actin/20uM DAPI.tif]

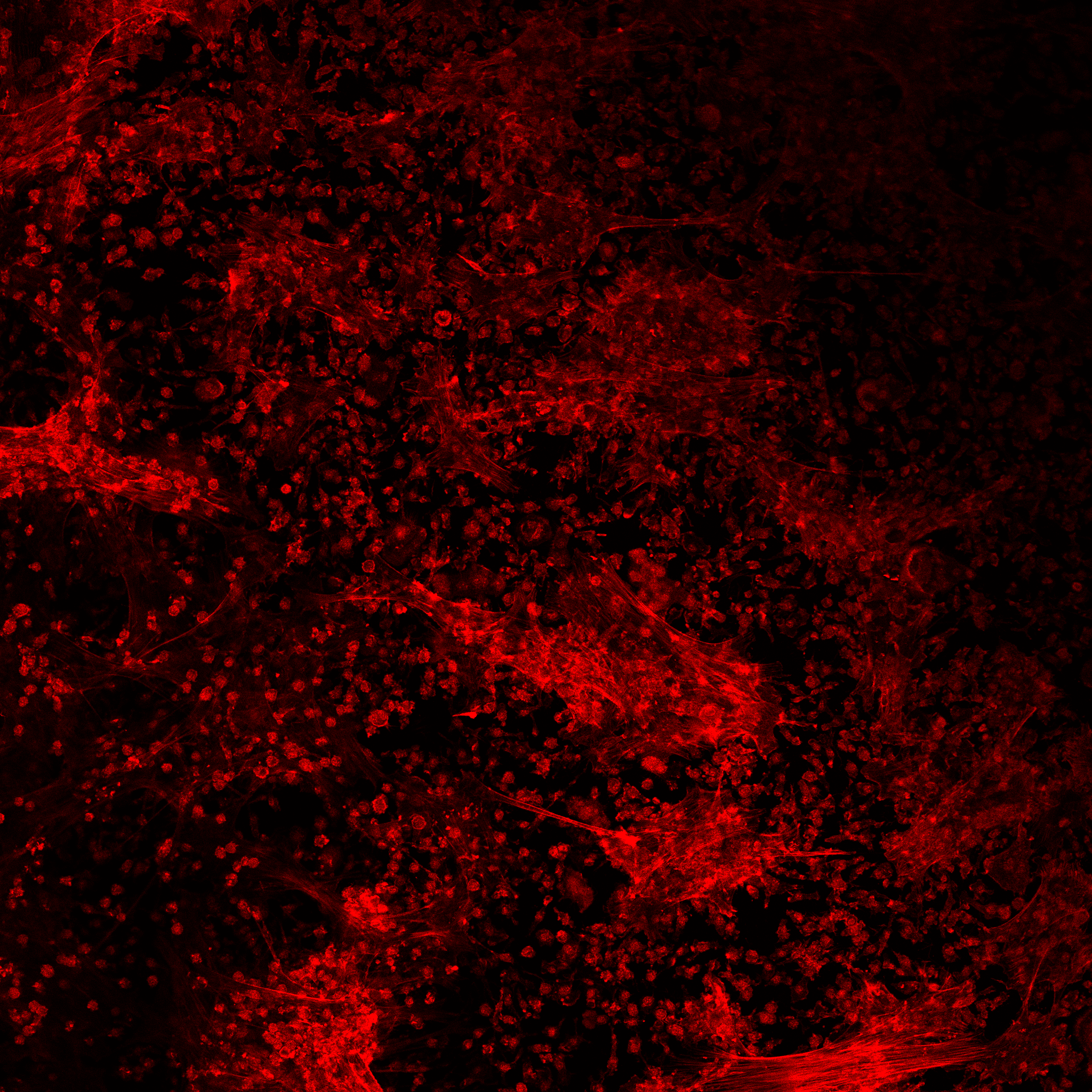

Supplement: Supplementary file 8 [file DataSheet2.ZIP › Data-figure3/F-actin/20uM f-actin.tif]

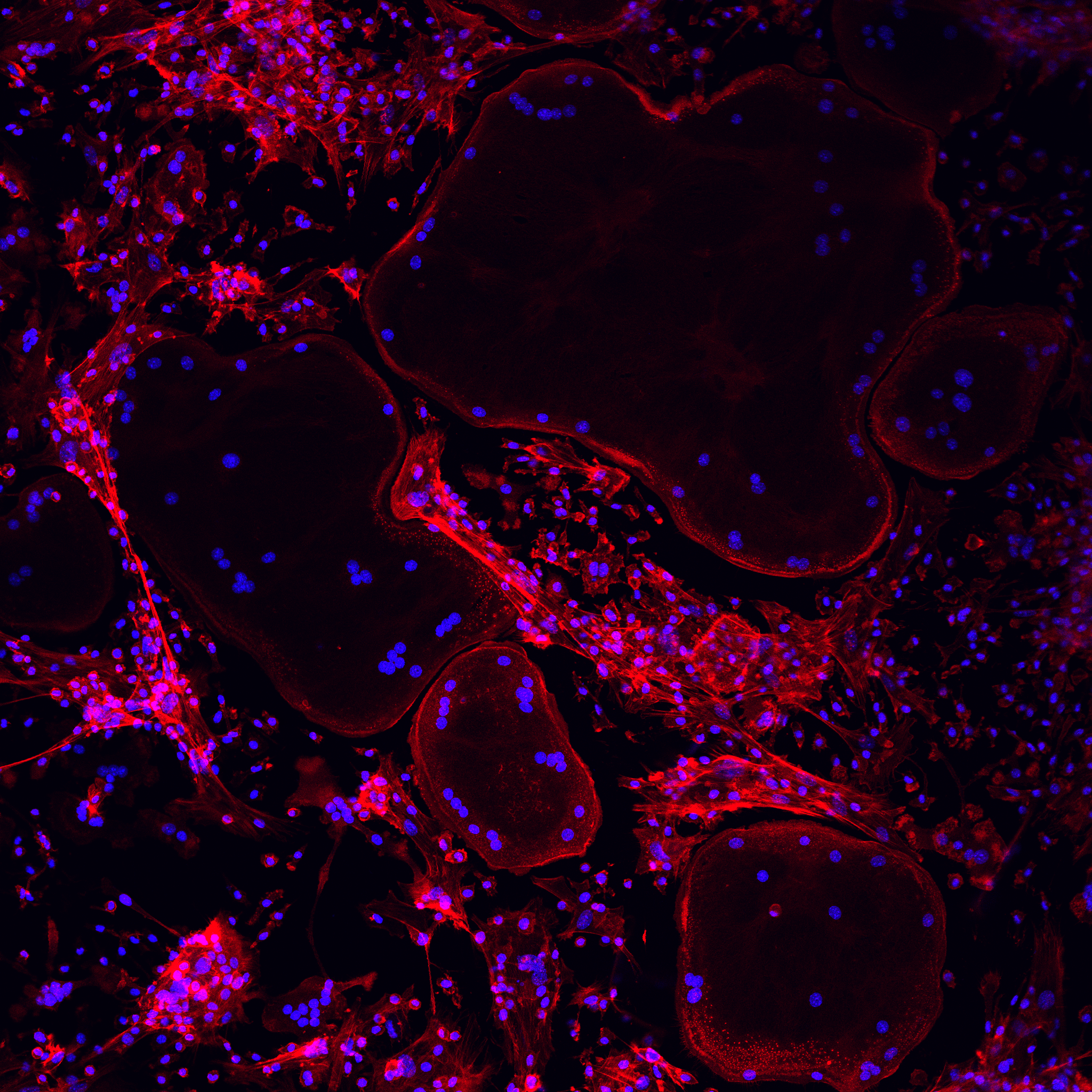

Supplement: Supplementary file 8 [file DataSheet2.ZIP › Data-figure3/F-actin/0uM merge.tif]

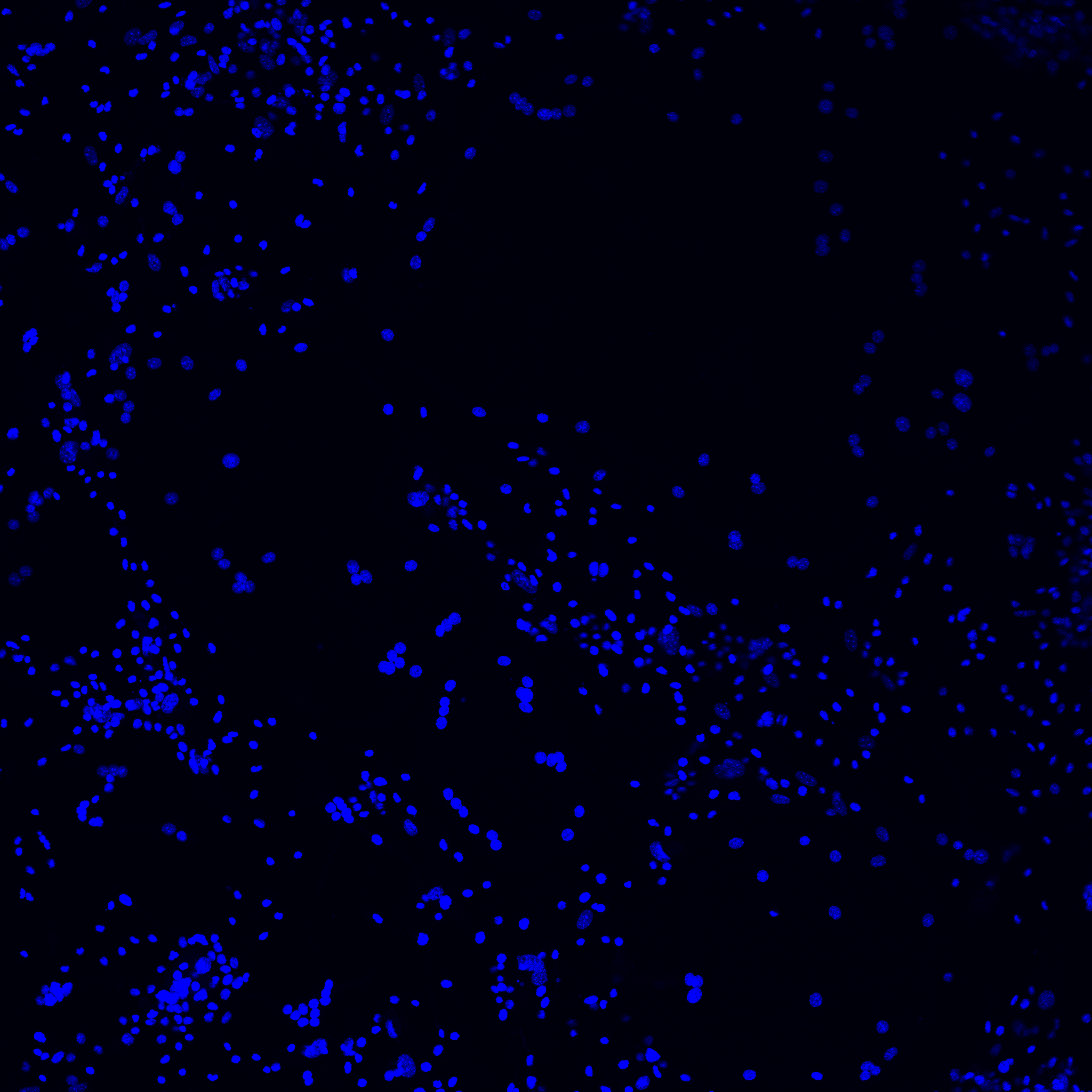

Supplement: Supplementary file 8 [file DataSheet2.ZIP › Data-figure3/F-actin/0uM DAPI.tif]

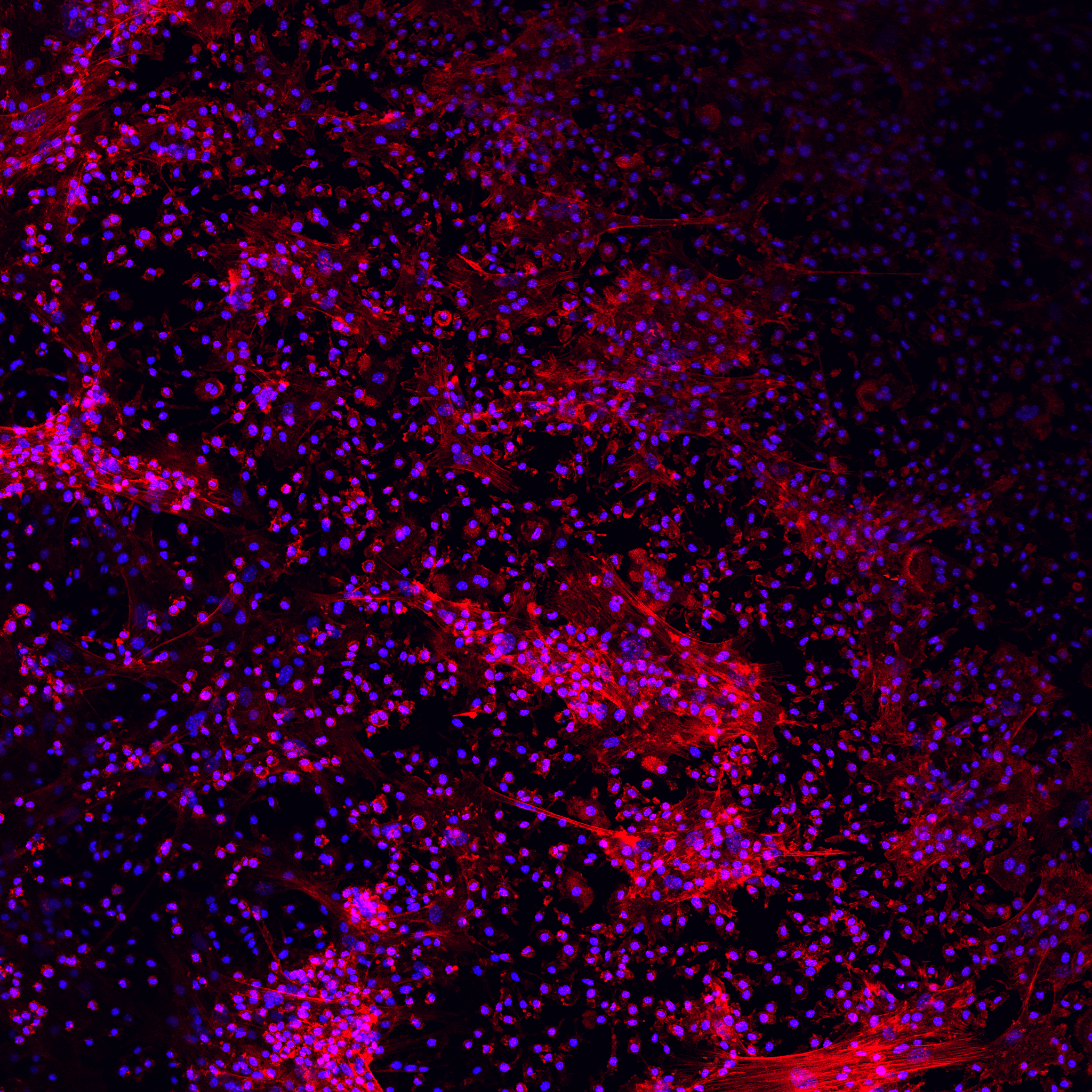

Supplement: Supplementary file 8 [file DataSheet2.ZIP › Data-figure3/F-actin/20uM merge.tif]

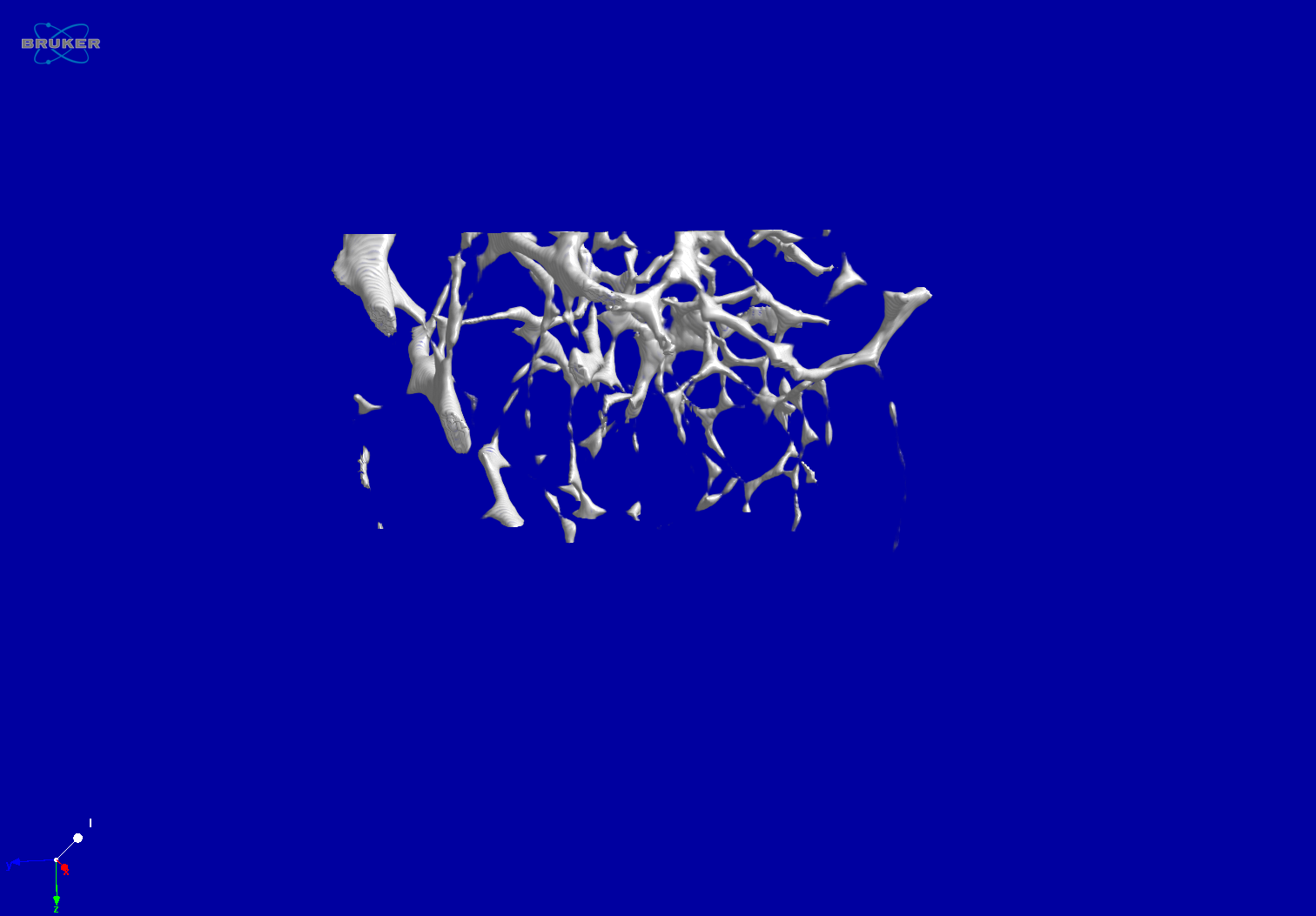

Supplement: Supplementary file 9 [file DataSheet5.ZIP › Data-figure6/vehicle.bmp]

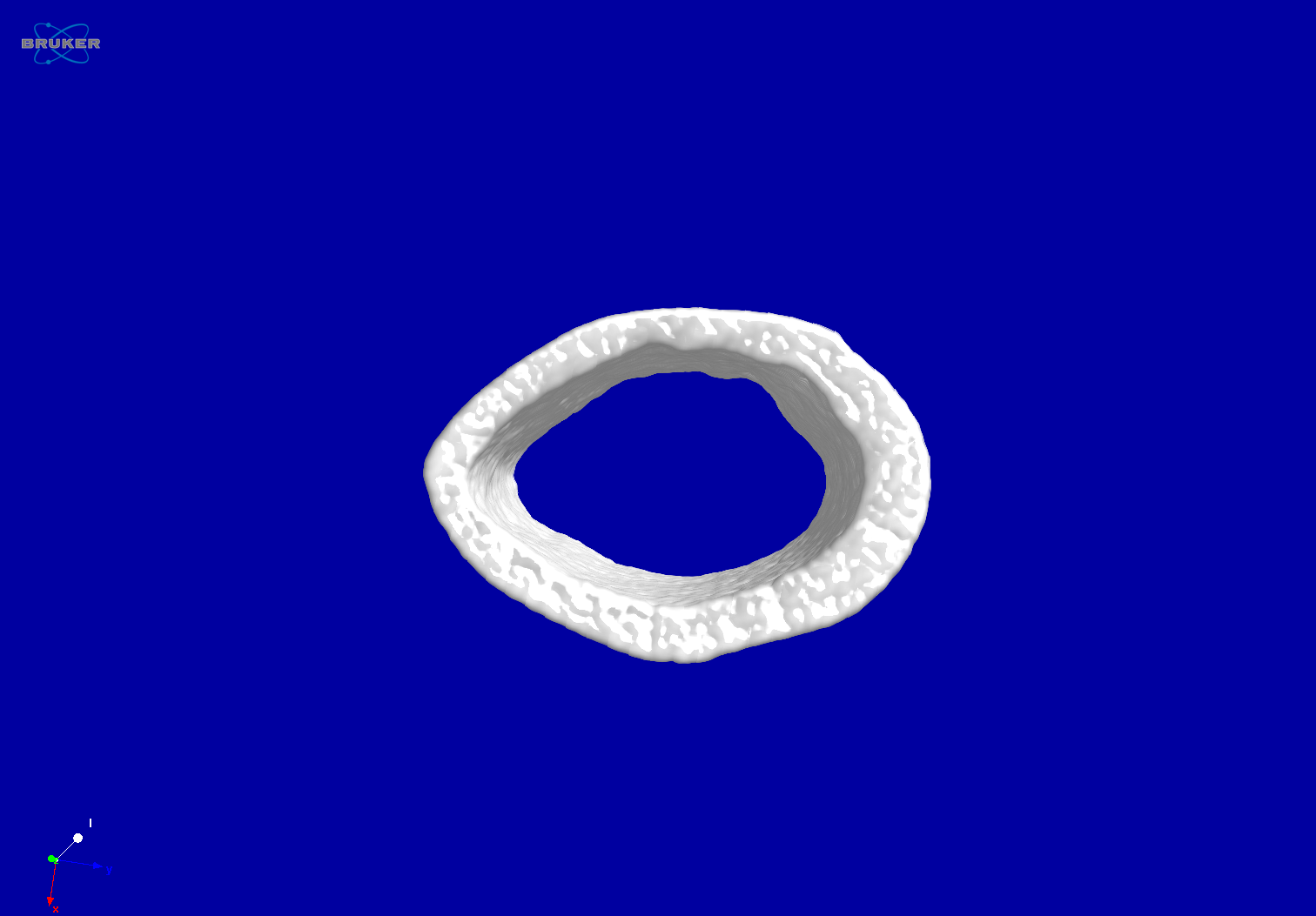

Supplement: Supplementary file 9 [file DataSheet5.ZIP › Data-figure6/vehicle-2.bmp]

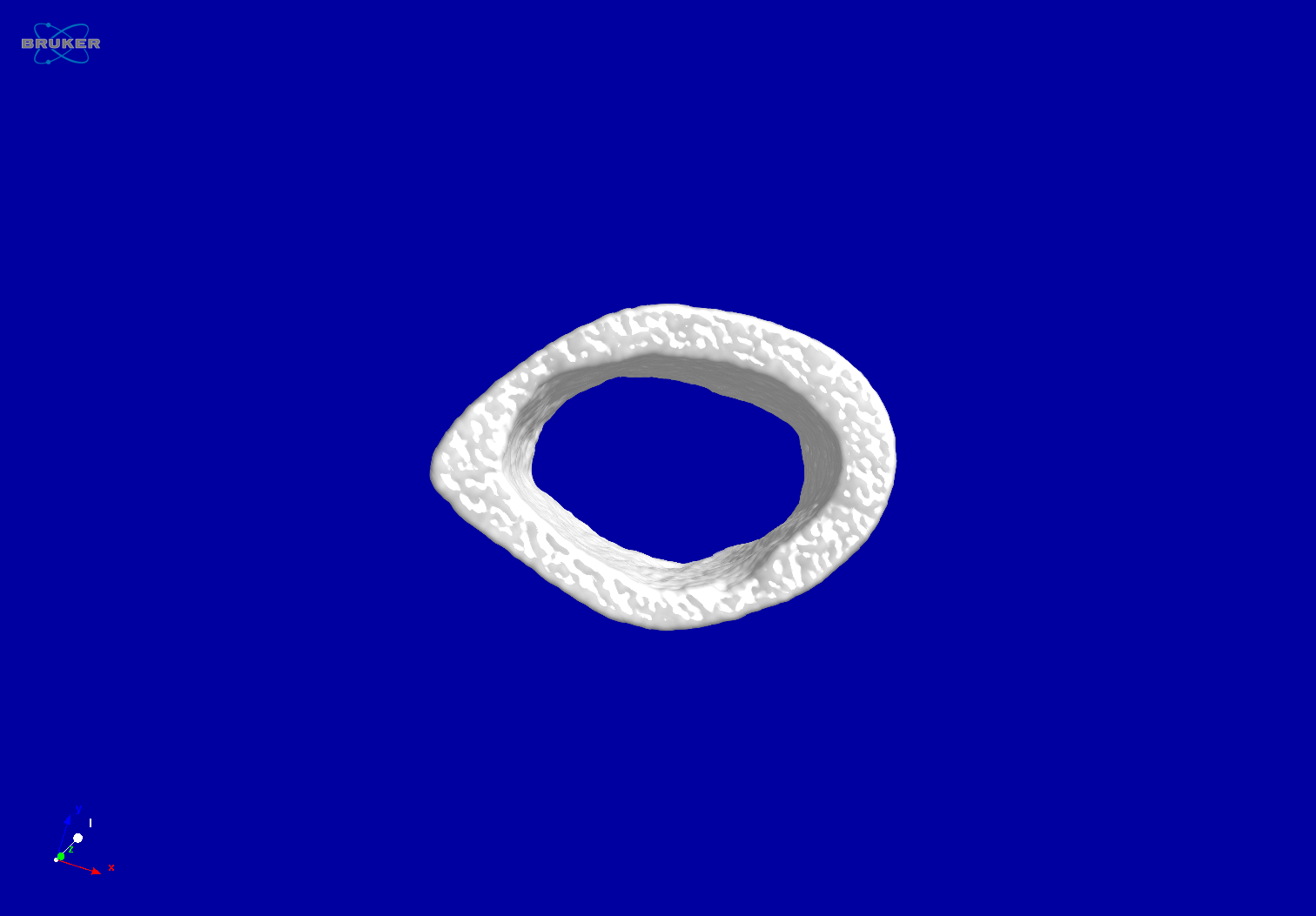

Supplement: Supplementary file 9 [file DataSheet5.ZIP › Data-figure6/mogrol-2.bmp]

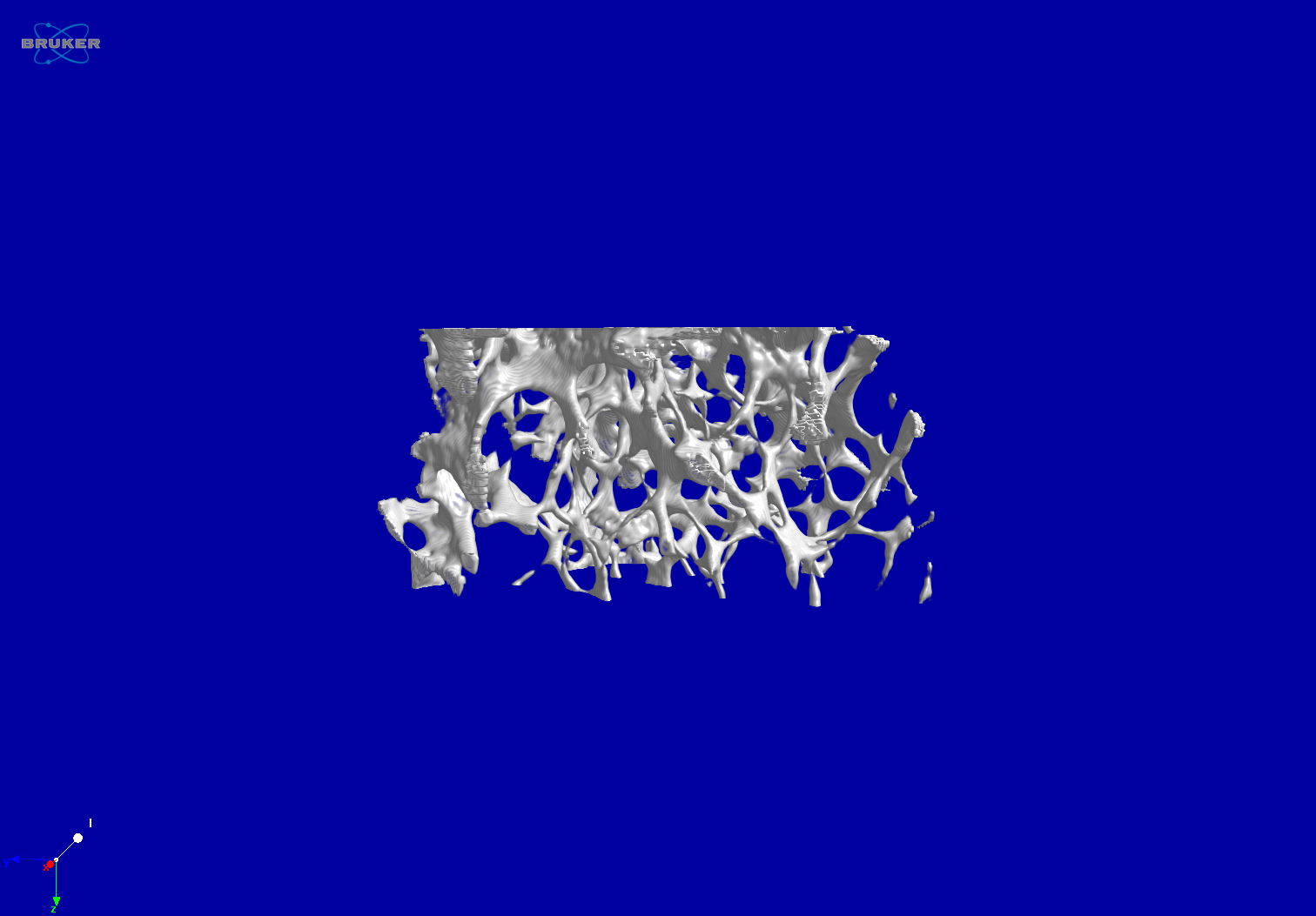

Supplement: Supplementary file 9 [file DataSheet5.ZIP › Data-figure6/sham.bmp]

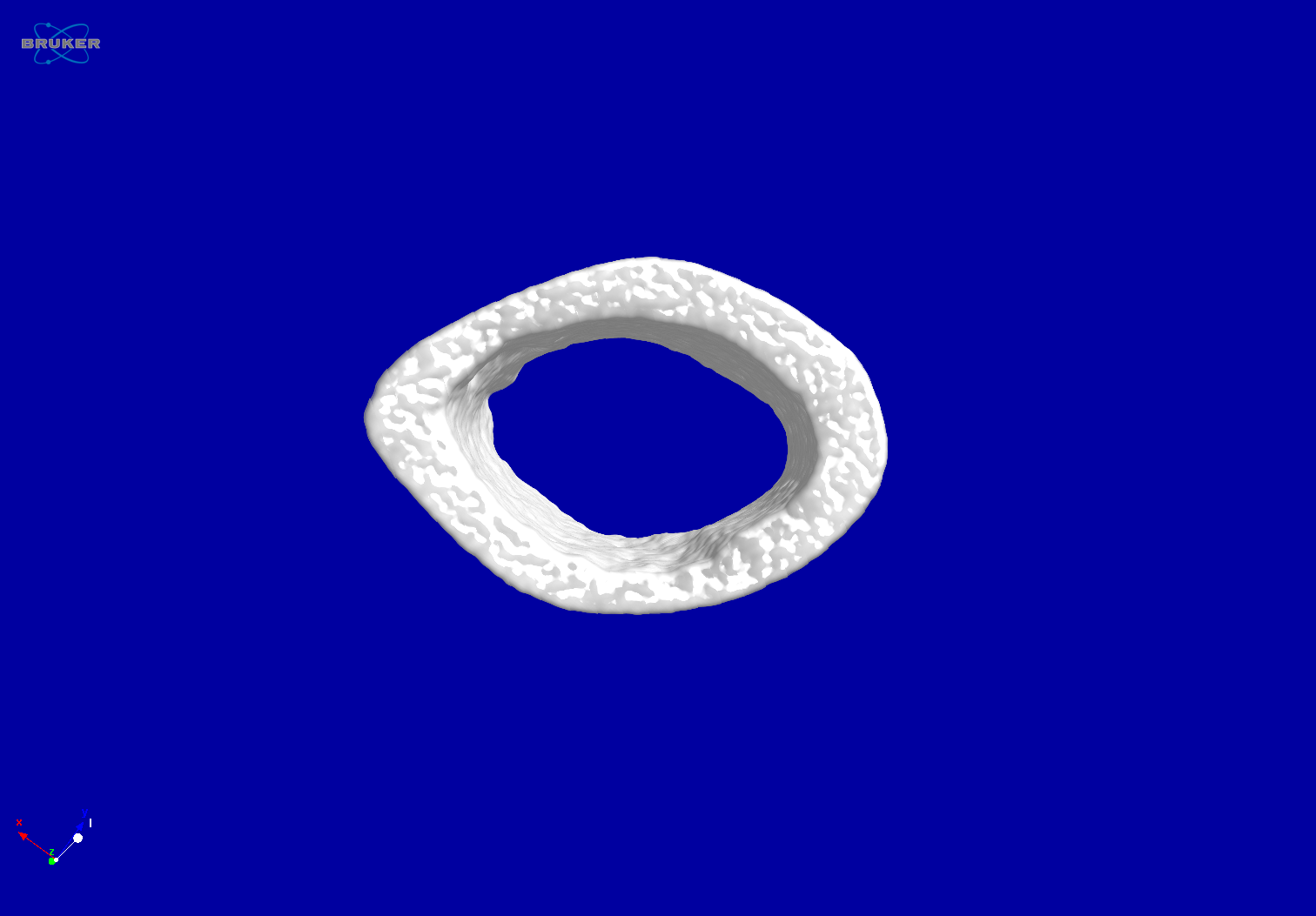

Supplement: Supplementary file 9 [file DataSheet5.ZIP › Data-figure6/sham-2.bmp]

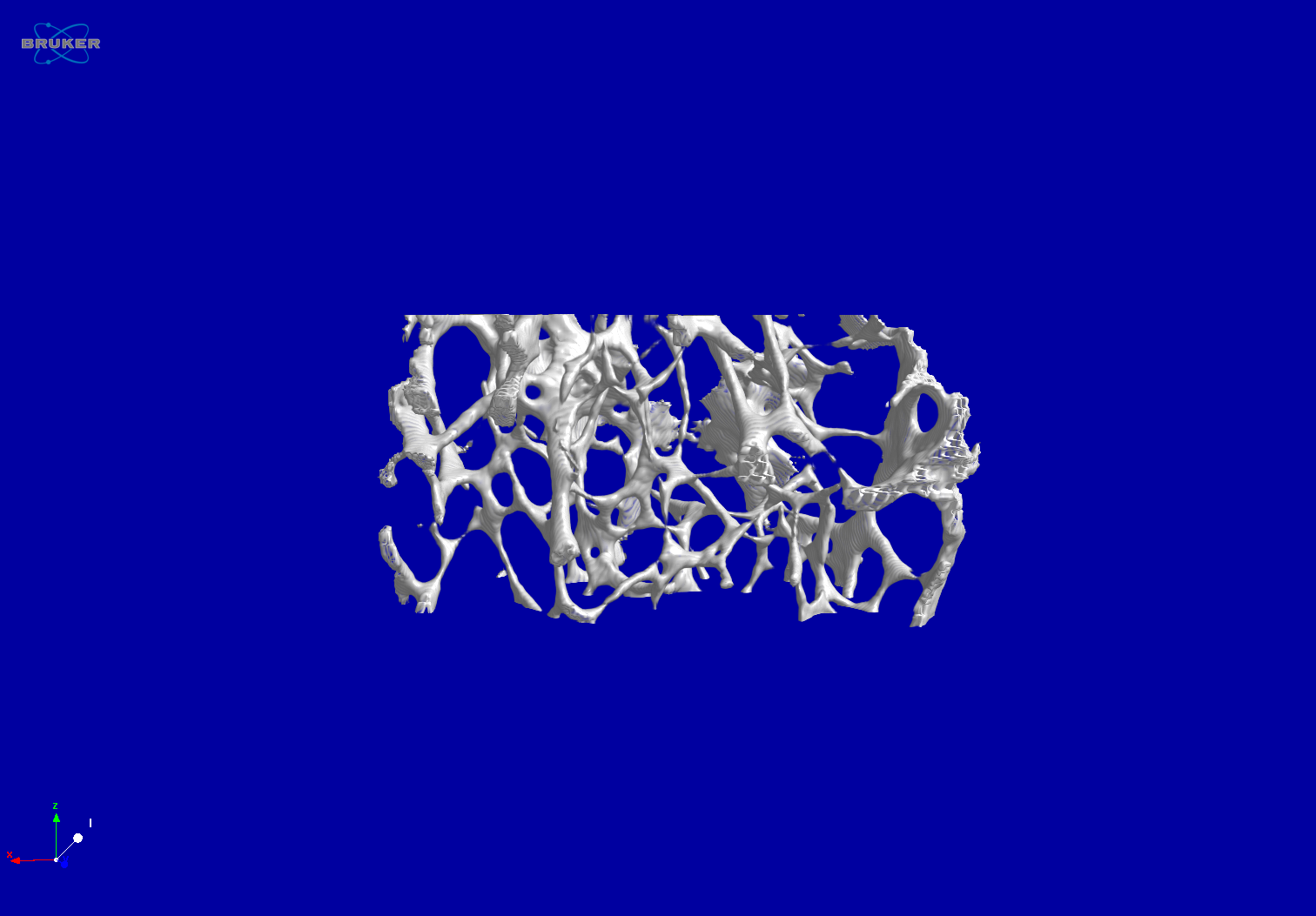

Supplement: Supplementary file 9 [file DataSheet5.ZIP › Data-figure6/mogrol.bmp]

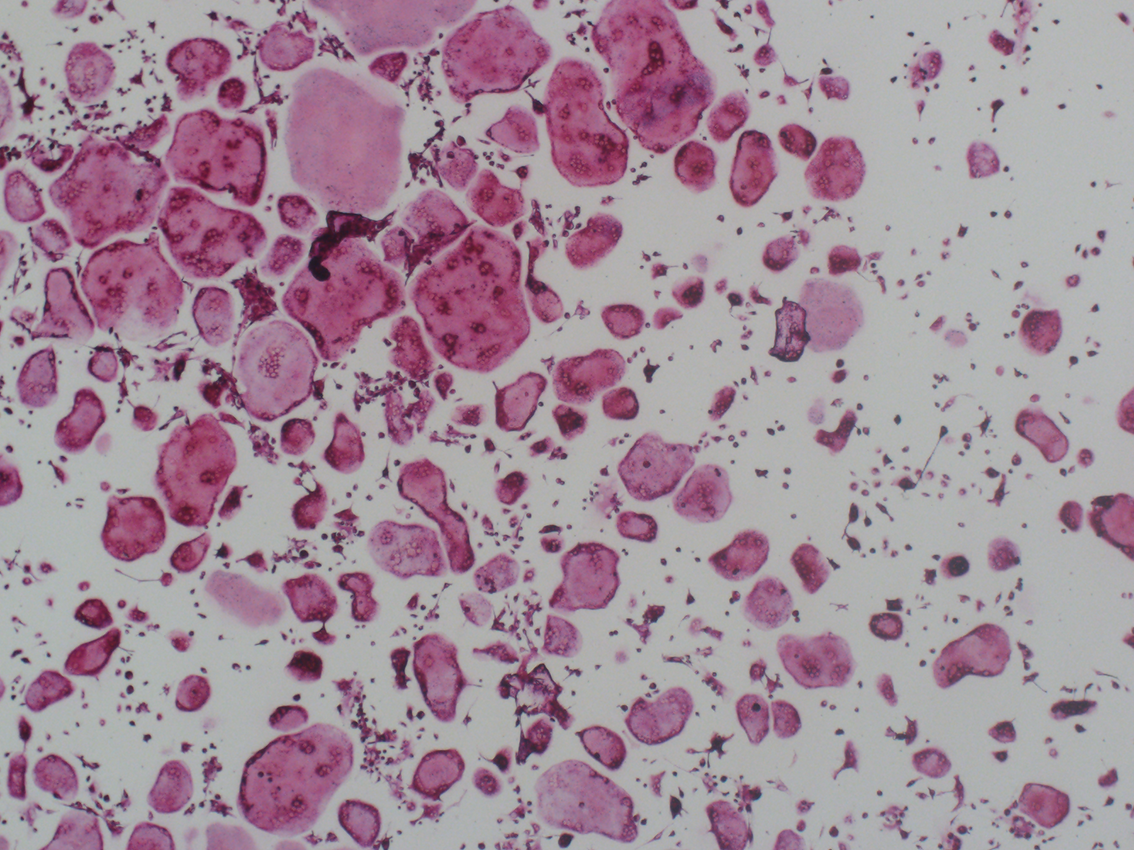

Supplement: Supplementary file 10 [file DataSheet7.ZIP › figure 2 data 2/time/RANKL-1.tif]

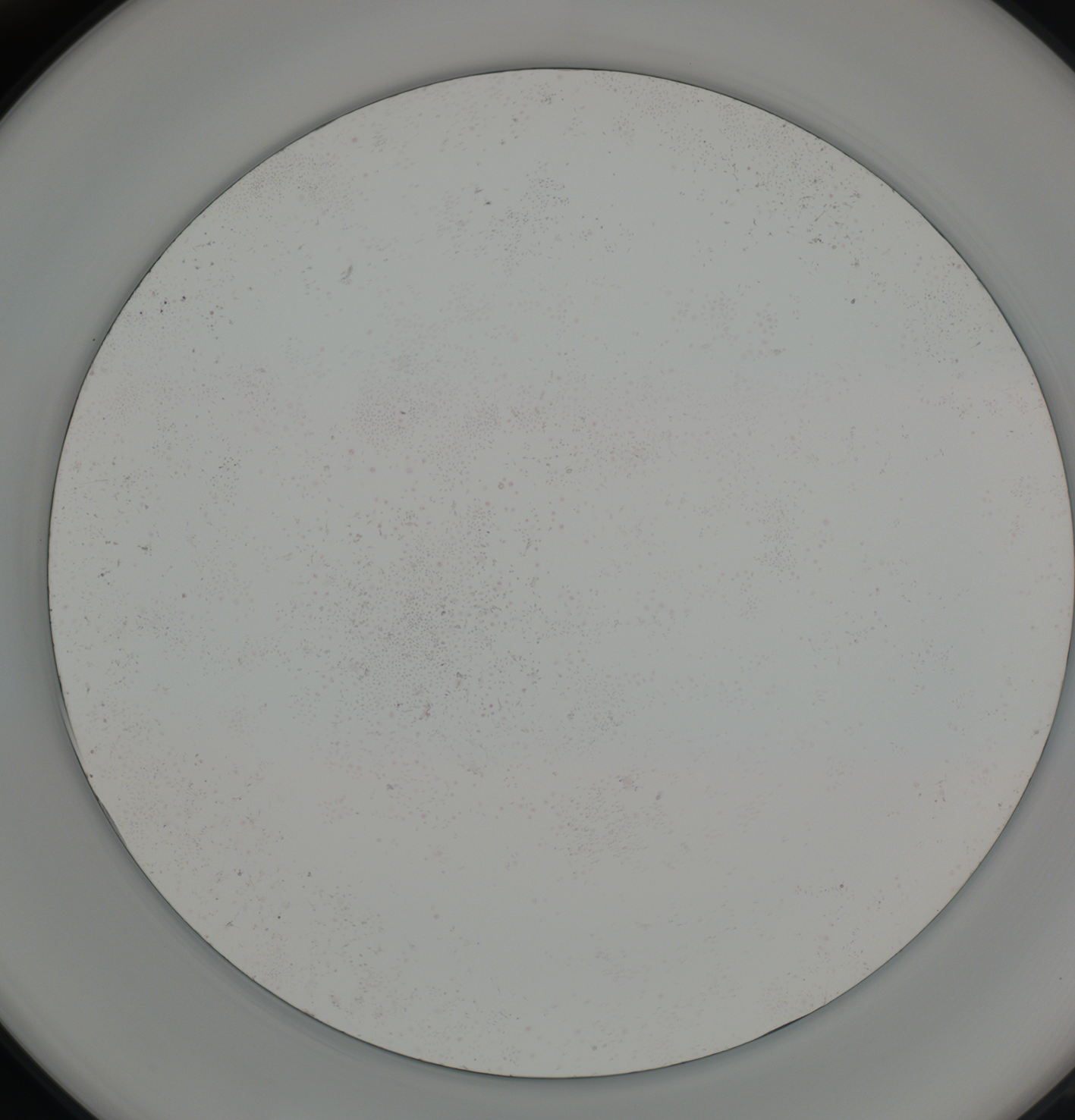

Supplement: Supplementary file 10 [file DataSheet7.ZIP › figure 2 data 2/time/-.tif]

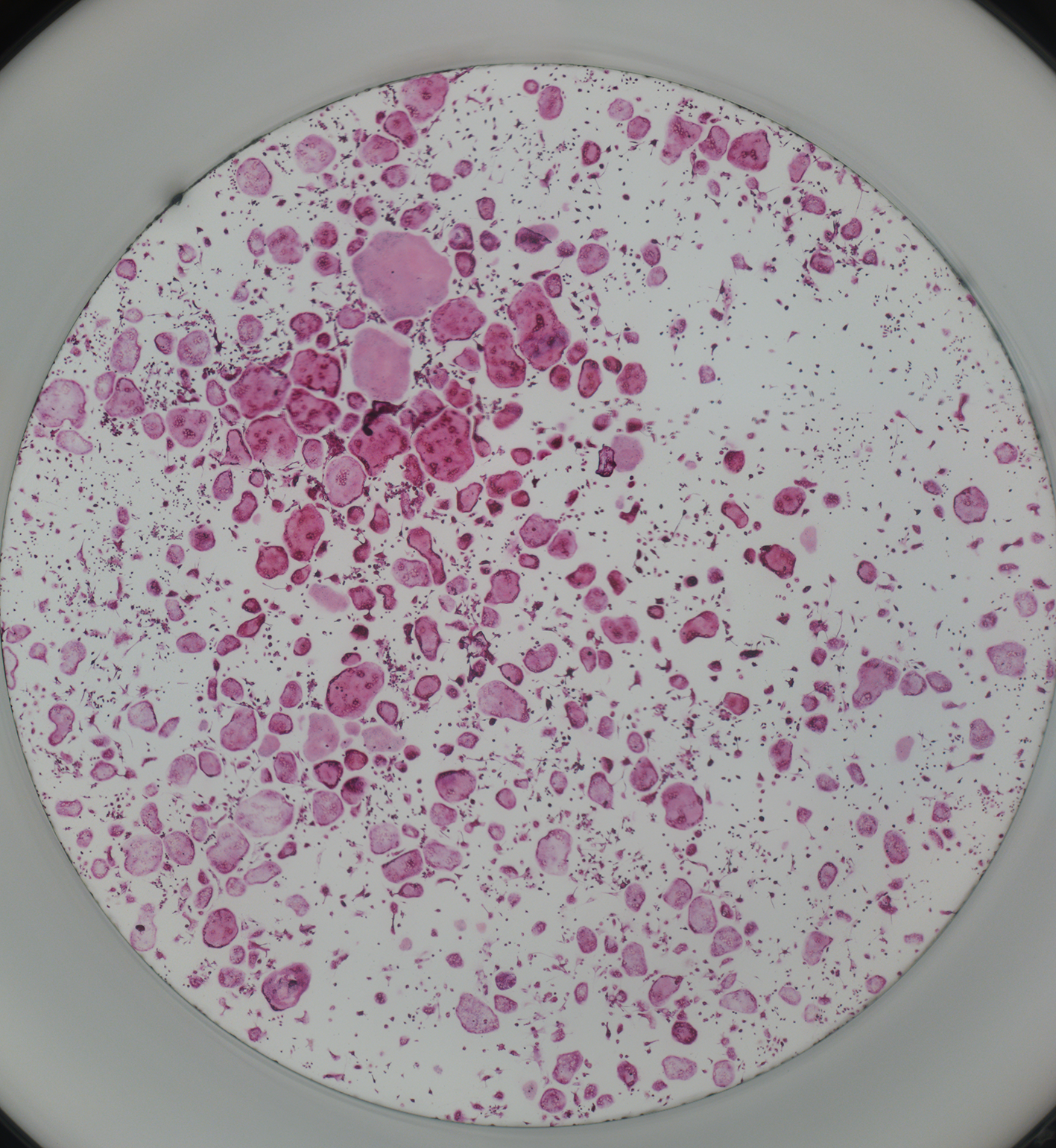

Supplement: Supplementary file 10 [file DataSheet7.ZIP › figure 2 data 2/time/RANKL.tif]

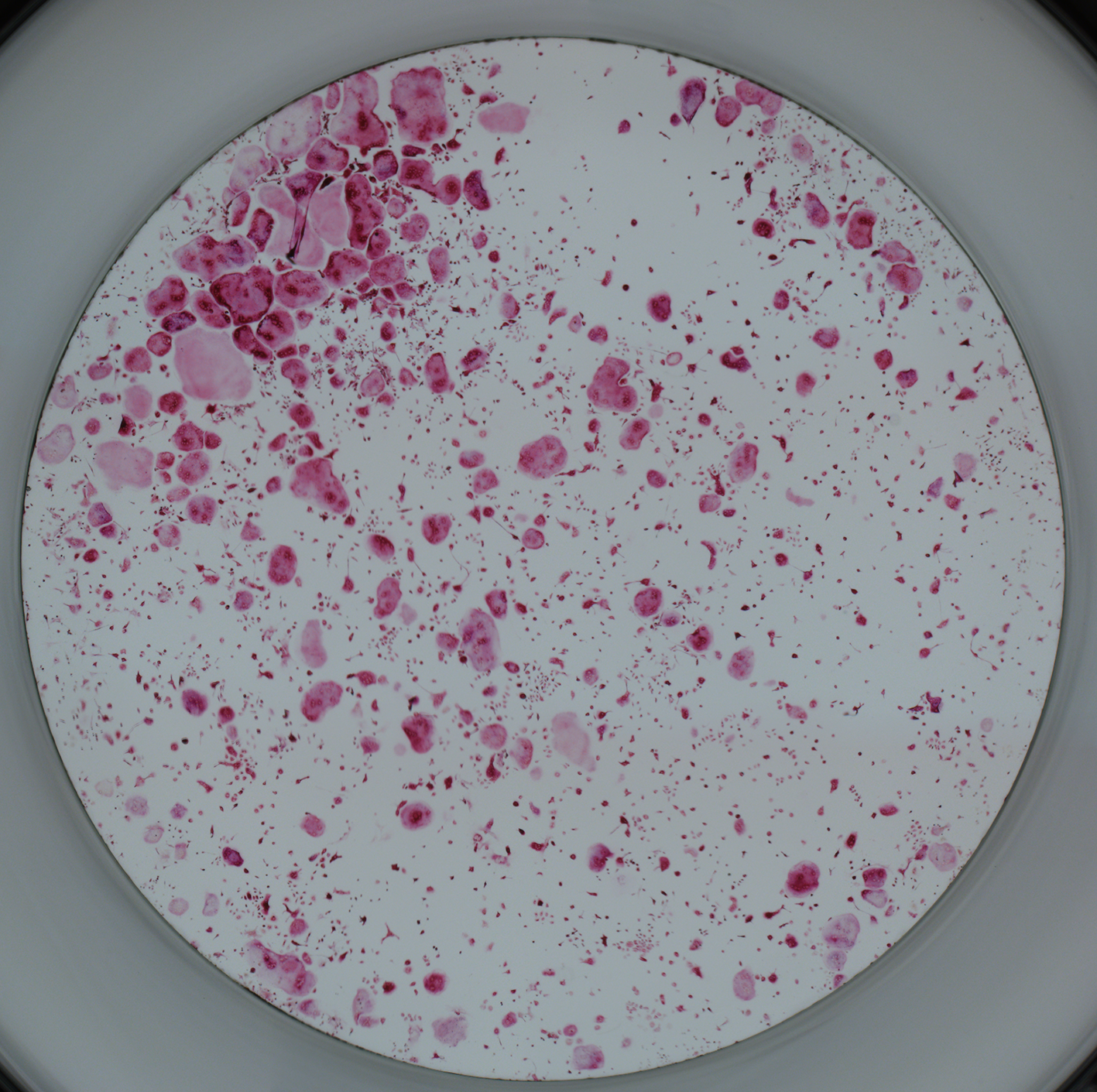

Supplement: Supplementary file 10 [file DataSheet7.ZIP › figure 2 data 2/time/D3-1.tif]

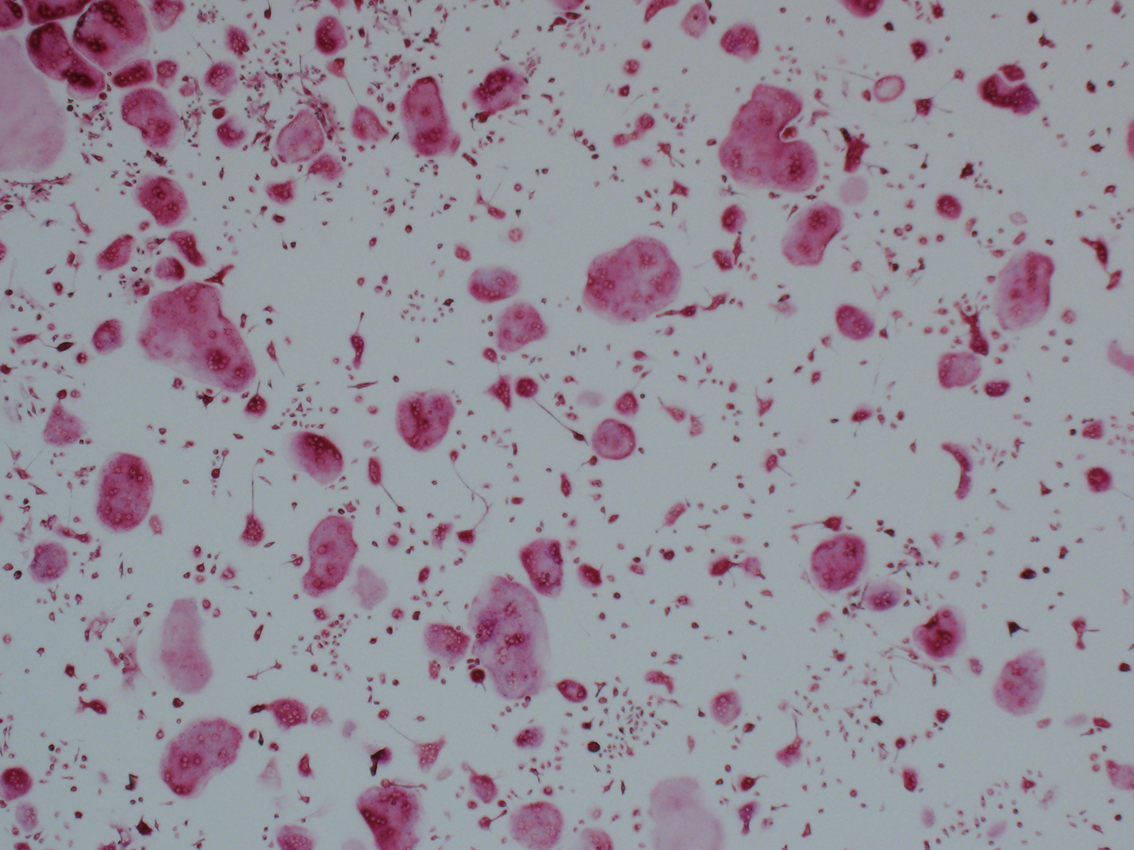

Supplement: Supplementary file 10 [file DataSheet7.ZIP › figure 2 data 2/time/D3-1ΓÇÖ.tif]

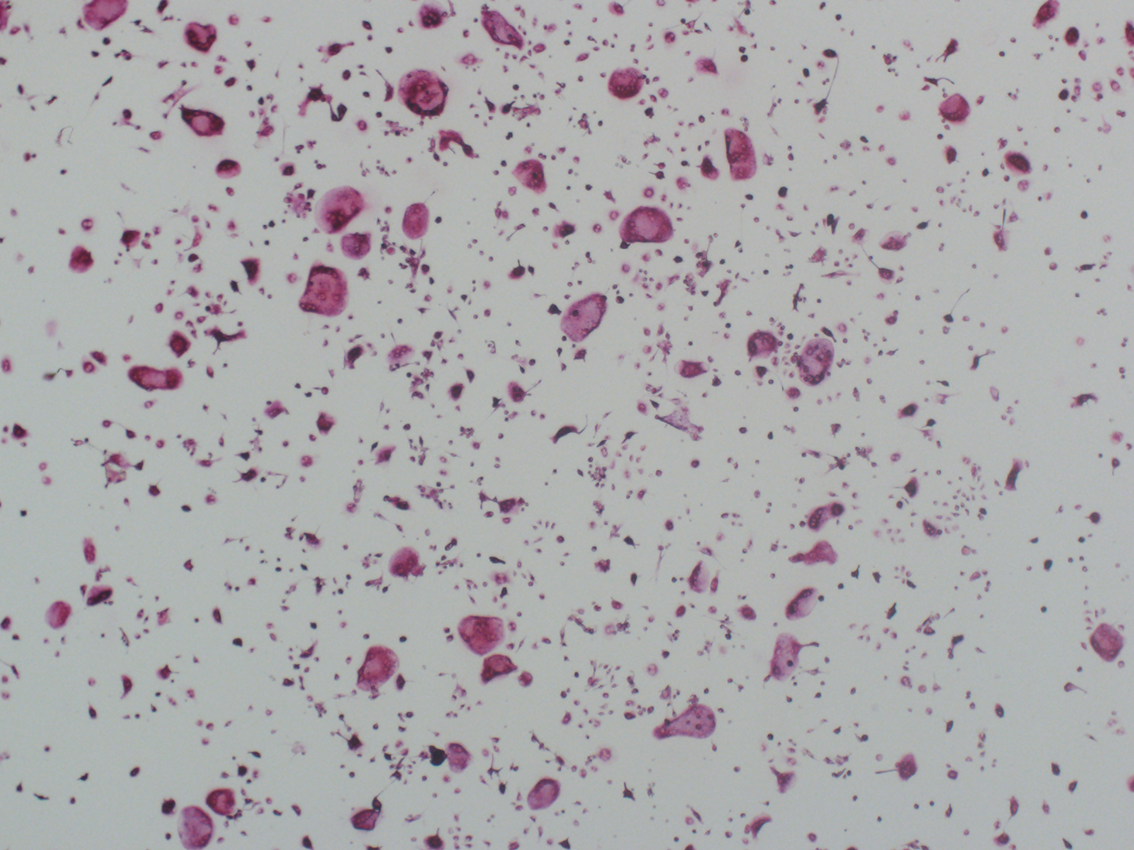

Supplement: Supplementary file 10 [file DataSheet7.ZIP › figure 2 data 2/time/D1-1.tif]

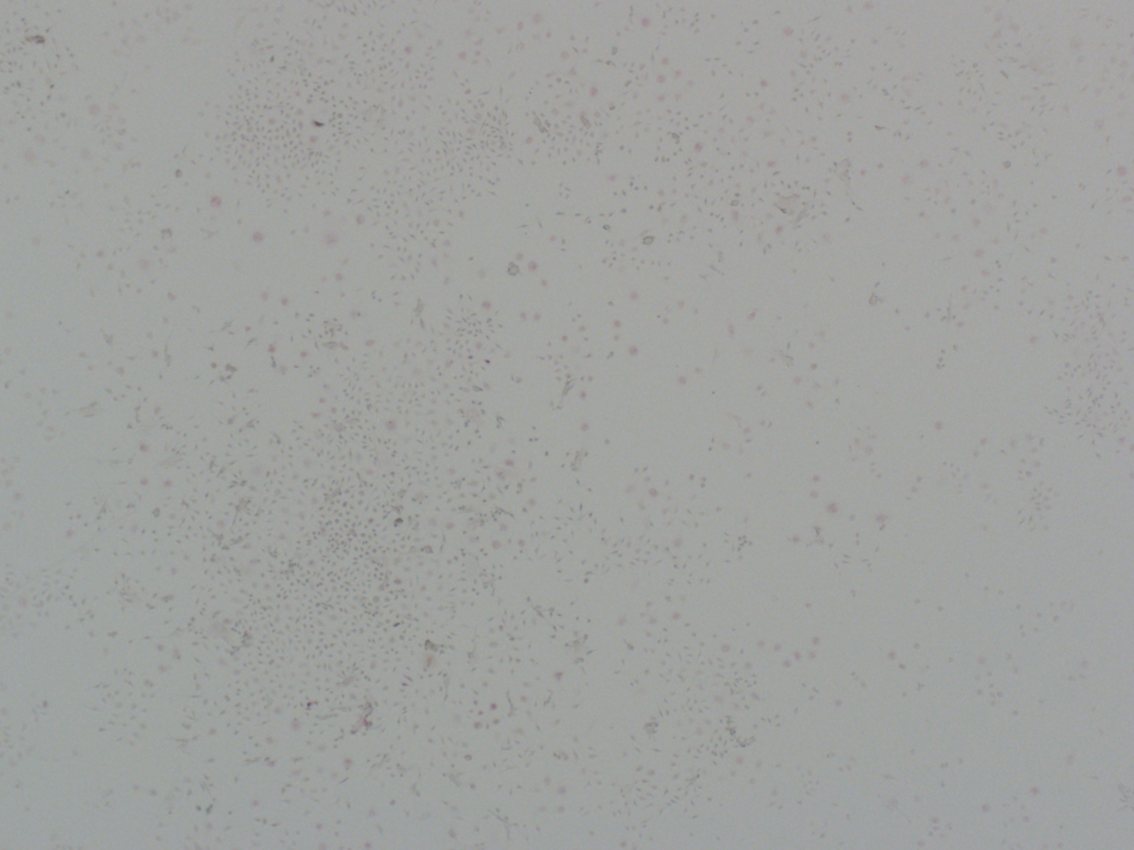

Supplement: Supplementary file 10 [file DataSheet7.ZIP › figure 2 data 2/time/-1.tif]

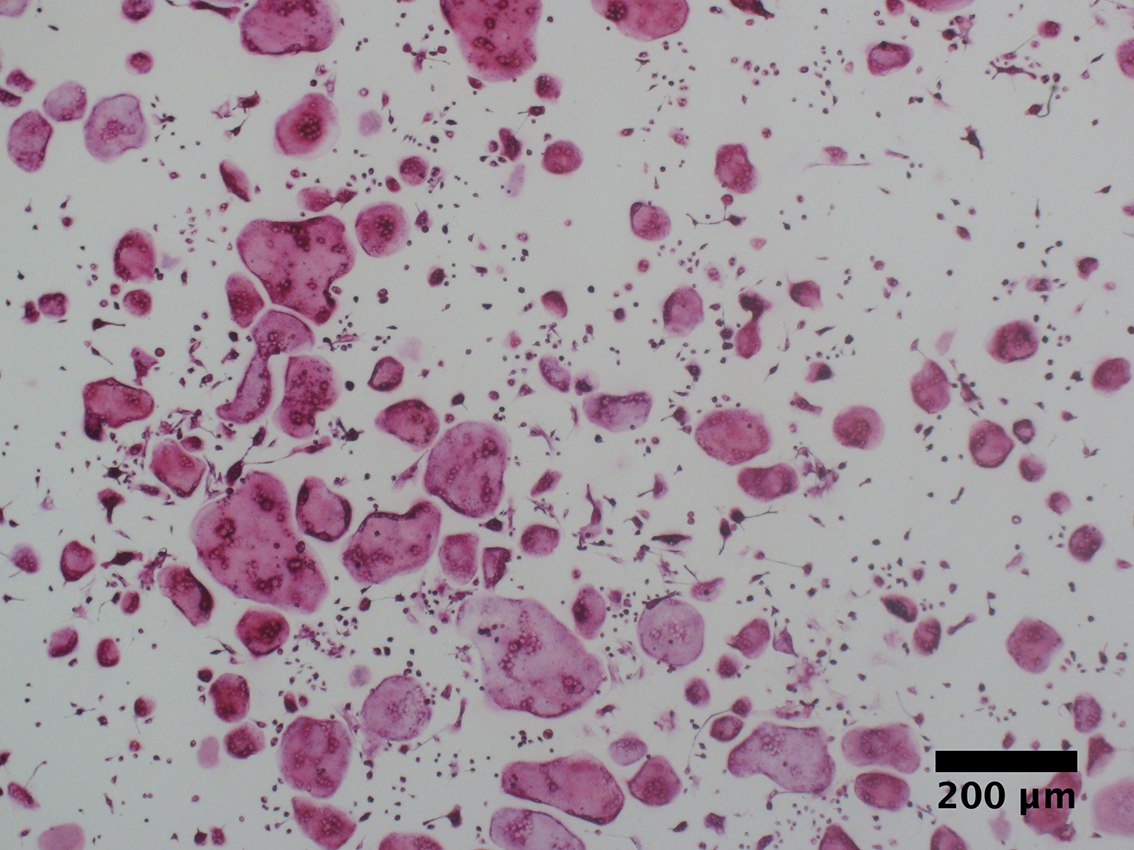

Supplement: Supplementary file 10 [file DataSheet7.ZIP › figure 2 data 2/time/D5-1.tif]

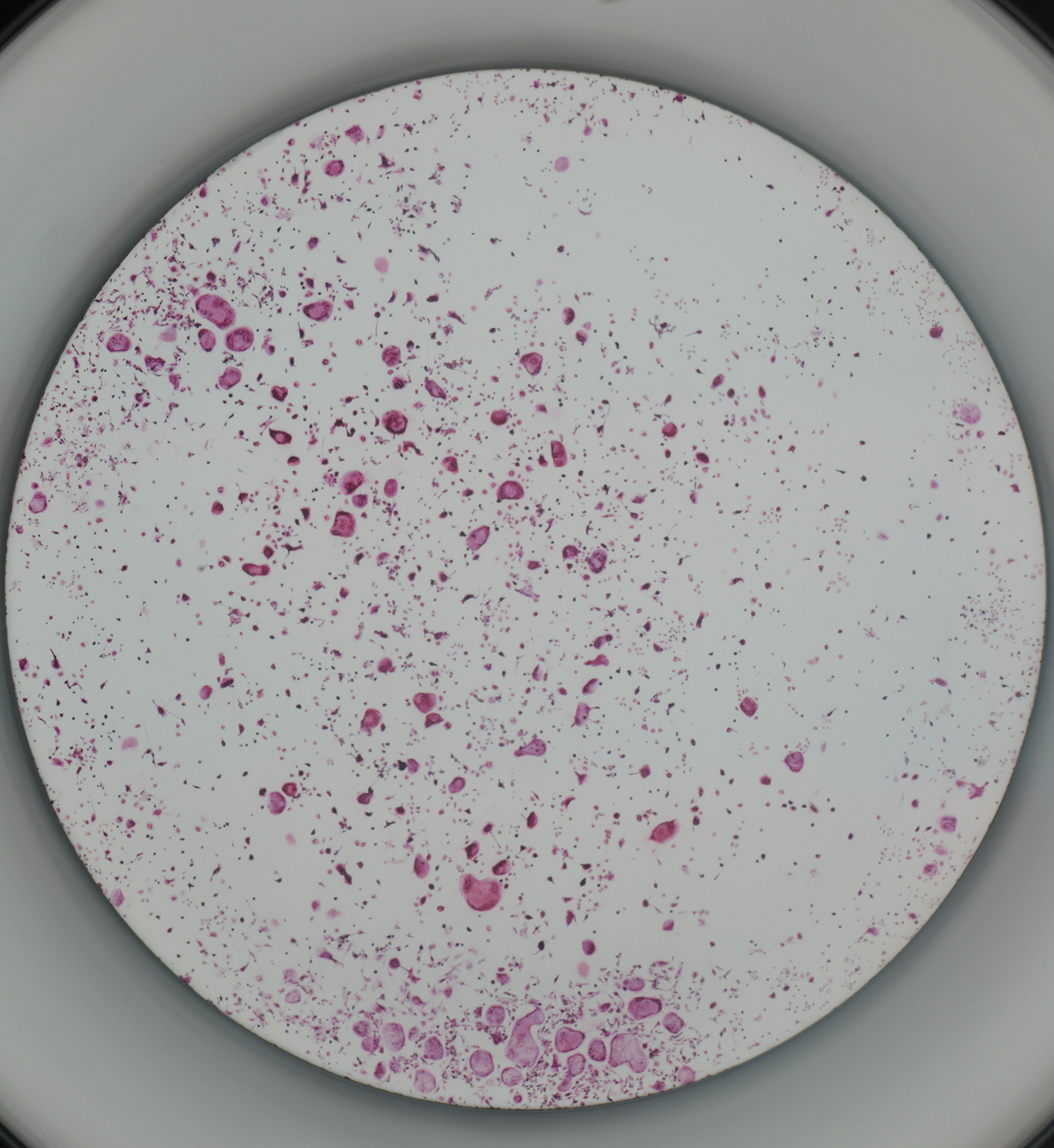

Supplement: Supplementary file 10 [file DataSheet7.ZIP › figure 2 data 2/time/D1.tif]

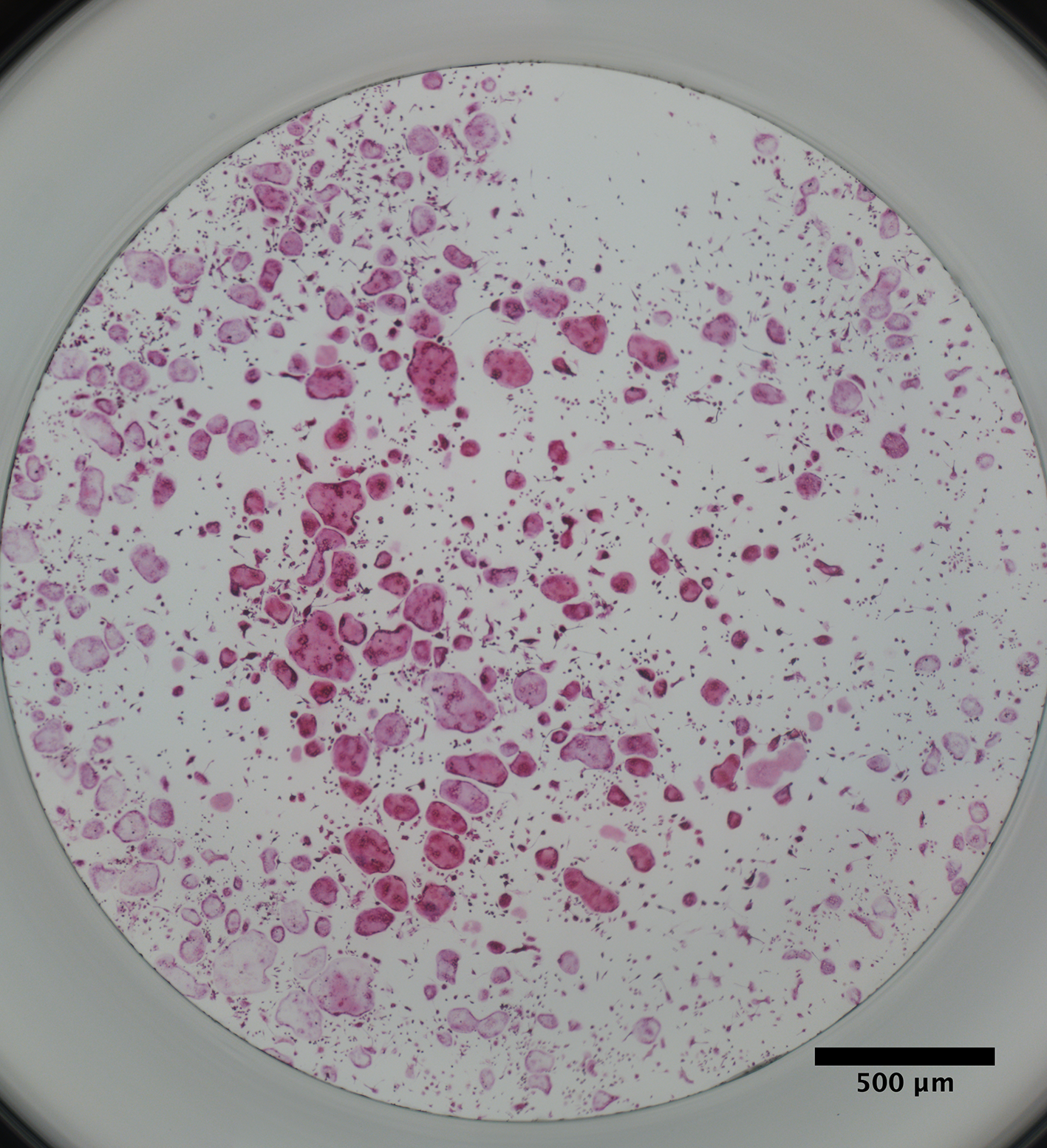

Supplement: Supplementary file 10 [file DataSheet7.ZIP › figure 2 data 2/time/D5.tif]
