# Supplementary material for: Mogrol Attenuates Osteoclast Formation and Bone Resorption by Inhibiting the TRAF6/MAPK/NF-κB Signaling Pathway In vitro and Protects Against Osteoporosis in Postmenopausal Mice
Source: Front Pharmacol. 2022 Mar 9;13:803880. doi: 10.3389/fphar.2022.803880 (PMC9038946; doi:10.3389/fphar.2022.803880)
Supplement: Supplementary file 11 [file Image1.pdf]

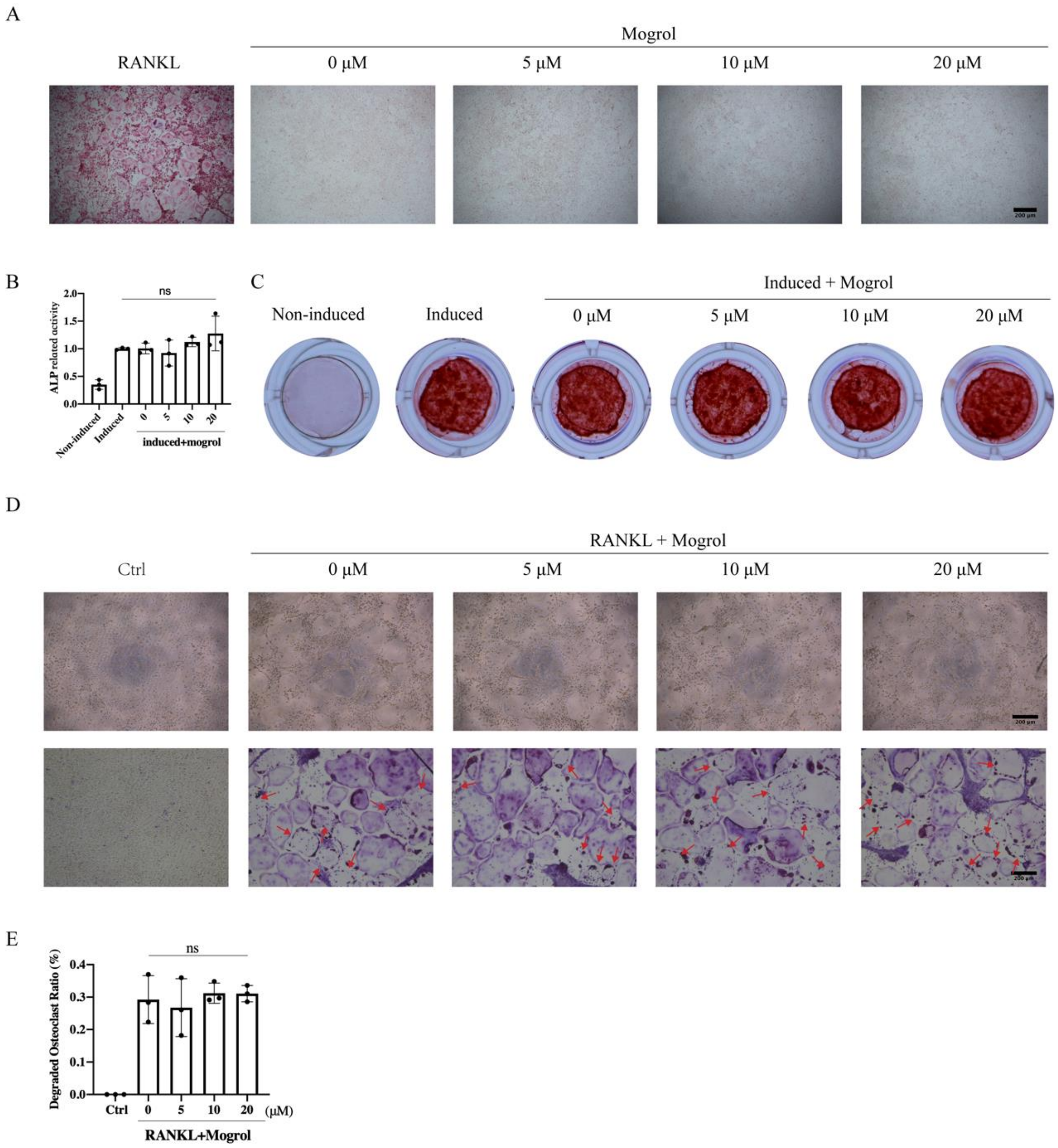

A. BMMs were cultured with complete α-MEM in the presence of different concentrations of mogrol (0, 5, 10, 20 μM) without stimulation of RANKL, while BMMs in the control group were stimulated with 30 ng/ml RANKL. After 5 days, TRAP staining was performed. The results demonstrated that mogrol treatment alone did not affect the differentiation of BMMs into mature osteoclasts.

B-C. Bone marrow stem cells (BMSCs) were flushed from the hind legs of 6-week-old C57BL/6J mice and cultured with complete α-MEM. After 5 days, the medium was replaced. The BMSCs were digested and seeded in 96-well plates at a density of 2×10^4 cells/well for subsequent experiments. The BMSCs were then cultured in α-MEM supplemented with 5 mM β-glycerol phosphate and 50 μg/mL ascorbic acid to induce differentiation into osteoblasts and cultured with different concentrations of mogrol, cells in 0 μM mogrol group were treated with 0.1% DMSO; cells in the blank group were not induced to differentiate. The medium was replaced every 3 days.

B. The alkaline phosphatase assay (ALP) was performed on the 7th day of differentiation. Before

ALP activity was measure, an Alamar blue assay was conducted according to the manufacturer's instructions. The cells were incubated with a solution containing 0.5 M Na_2CO_3, 0.5 M $NaHCO_3$, 1 mM $MgCl_2$ and 1 mg/ml phosphatase substrate (Sigma–Aldrich, St. Louis, MO, USA) at 37 °C for 20 min. Then, the plate was scanned with a reader (Biotek, USA) at 450 nm.

C. Alizarin Red S staining was performed on the 14th day of differentiation to study the effect of mogrol on the mineralization of osteoblasts. After fixation with 4% PFA, the cells were stained with Alizarin Red S dye (40 mM, pH 4.2, Sigma–Aldrich) for 10 min. There were no significant differences in staining between the mogrol-treated group and the induced group. These findings indicated that mogrol did not affect bone matrix synthesis or mineralization of osteoblasts.

D-E. To observe the effect of mogrol on mature osteoclasts, BMMs were cultured with complete α-MEM without (control) or 30 ng/ml RANKL for 5 days. After they formed mature osteoclasts, the cells were treated with different concentrations of mogrol (0, 5, 10, 20 μM). TRAP staining was conducted to observe the degradation of osteoclasts at approximately 36 hours. D. Representative images of mature osteoclasts and TRAP staining. E. Quantitative analysis of degraded osteoclasts. The results demonstrated that mogrol had no effect on mature osteoclast degradation. The data are shown as the mean ± SD and were analyzed using ANOVA followed by Dunnett's post hoc test. Scale bar= 200 μm.
